# A Rare Low‐Spin Co^IV^ Bis(β‐silyldiamide) with High Thermal Stability: Steric Enforcement of a Doublet Configuration†

**DOI:** 10.1002/anie.202001518

**Published:** 2020-06-08

**Authors:** David Zanders, Goran Bačić, Dominique Leckie, Oluwadamilola Odegbesan, Jeremy Rawson, Jason D. Masuda, Anjana Devi, Seán T. Barry

**Affiliations:** ^1^ Inorganic Materials Chemistry Faculty of Chemistry and Biochemistry Ruhr University Bochum Universitätsstraße 150 44801 Bochum Germany; ^2^ Department of Chemistry Carleton University 1125 Colonel By Drive Ottawa Ontario K1S 5B6 Canada; ^3^ Department of Chemistry and Biochemistry University of Windsor 401 Sunset Avenue Windsor Ontario N9B 3P4 Canada; ^4^ Department of Chemistry Saint Mary's University 923 Robie Street Halifax Nova Scotia B3H 3C3 Canada

**Keywords:** atomic layer deposition, cobalt, coupled-cluster calculations, density functional calculations, EPR spectroscopy

## Abstract

Attempted preparation of a chelated Co^II^ β‐silylamide resulted in the unprecedented disproportionation to Co^0^ and a spirocyclic cobalt(IV) bis(β‐silyldiamide): [Co[(N^t^Bu)_2_SiMe_2_]_2_] (**1**). Compound **1** exhibited a room‐temperature magnetic moment of 1.8 B.M. and a solid‐state axial EPR spectrum diagnostic of a rare S=1/2 configuration for tetrahedral Co^IV^. Ab initio semicanonical coupled‐cluster calculations (DLPNO‐CCSD(T)) revealed the doublet state was clearly preferred (−27 kcal mol^−1^) over higher spin configurations only for the bulky tert‐butyl‐substituted analogue. Unlike other Co^IV^ complexes, **1** had remarkable thermal stability, and was demonstrated to form a stable self‐limiting monolayer in preliminary atomic layer deposition (ALD) surface saturation experiments. The ease of synthesis and high stability make **1** an attractive starting point to investigate otherwise inaccessible Co^IV^ intermediates and for synthesizing new materials.

## Graphical abstract

**The role of spatial arrangement**: Owing to ligand rigidity and spatial demand, the stabilization of a rare *S*=1/2 spin ground state for tetracoordinate Co^IV^ becomes possible, yielding a complex with high thermal stability and potential for applications relying on atomic layer deposition.

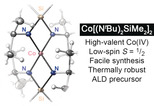

Cobalt complexes are effective and cost‐efficient alternatives to Ir‐ and Rh‐based catalysts for a number of important C−H bond transformations,^1^ alkynylations,^2^ alkylations, and arylations.^3^ Recently, high‐valent Co^IV^ species have also been shown to affect C−H bond activation.^4^ High‐valence Co‐containing materials like the electrode material Li_2−*x*_CoO_2−*δ*_ are also essential as the cathode material of Li‐ion batteries,^5^ and others such as CoS_x_ have been predicted to become key materials in future green energy photocatalysts.^6^ The facile synthesis of robust Co^IV^ complexes has remained a challenge, so their exploitation in the field of catalysis as active species and in materials science as precursors for material synthesis has remained limited. Chemical vapor phase (CVD) techniques such as atomic layer deposition (ALD) have become one of the most preferable approaches for the fabrication of exotic, emerging materials.^7^ So it stands to reason to evaluate Co^IV^ complexes not only in light of their electronic nature but also with respect to their physico‐chemical properties which might qualify them as CVD/ALD precursors.

To date, most Co^IV^ complexes have been prepared by 1 e^−^ oxidation of Co^III^ complexes with Br_2_, Fe(ClO_4_)_3_, or by cyclic voltammetry. The starting Co^III^ complexes typically comprise sterically demanding tetraamido or pyrrolato macrocycles, and the resulting Co^IV^ species are commonly unstable above room temperature. In cases where unambiguous Co^IV^ species could be isolated, geometries are typically limited to square planar and square pyramidal.^8^ Macrocyclic Co^IV^ oxo‐congeners such as the one introduced by Wang et al. are typically more robust but still suffer from this geometrical restriction.^9^

For d^5^ ions, the high‐spin *S*=5/2 configuration exhibits the maximum exchange energy for transition metals; thus, examples of low‐spin d^5^ are rare. This is especially the case for tetrahedral geometries where the ligand field splitting is typically small in relation to octahedral fields. However, for Co^IV^, the increased oxidation state affords larger ligand fields and so a finer energetic balance is expected between high‐ and low‐spin electronic configurations. Interesting examples are Deng's trigonal‐planar, N‐heterocyclic carbene stabilized Co^IV^ bis‐imide^10^ and Groysman's tricoordinate, diarylcarbene‐stabilized Co^IV^ bis‐alkoxide.^4^ While computational studies showed that the assignment of the spectroscopic oxidation state for the metal centers was ambiguous, their magnetic properties supported a *S*=1/2 ground state in each case. More recently, a squashed‐tetrahedral Co^IV^ tetrakis(ketimide) Co(N=C^t^Bu_2_)_4_ was reported, but it exhibited a quartet ground state (*S*=3/2 ).^11^ Co^IV^ tetrakis(1‐norbonyl), Co(nor)_4_, has remained the only known unambiguous example of a tetracoordinate low‐spin Co^IV^ complex for over 40 years.^12^, ^13^

β‐Silylamines are excellent ligands to stabilize unusual coordination numbers and oxidation states for elements across the periodic table.^14^ The good thermal stability and high volatility of the well‐known three‐coordinate Co^II^ complex {Co[N(SiMe_3_)_2_]_2_}_2_ prompted us to explore other Co β‐silylamides as potential precursors for vapor phase deposition of Co‐containing materials.

Here, we report the serendipitous preparation of 1,3,5,7‐tetra‐*tert*‐butyl‐2,2,6,6‐tetramethyl‐1,3,5,7‐tetraaza‐2,6‐disila‐4λ^4^‐cobaltaspiro[3.3]heptane, {Co[(N^t^Bu)_2_SiMe_2_]_2_} (**1**), the second, unambiguous Co^IV^ complex with a low‐spin *S*=1/2 ground state configuration. We have implemented both experiment and theory to understand its unusual electronic nature. Finally, compound **1** has electronic properties that are complemented by physico‐chemical features that set it apart from all prior reported Co^IV^ complexes and make it especially appealing for application in ALD.

Treatment of suspended CoCl_2_(TMEDA) (TMEDA=tetramethylethylenediamine) with one equivalent of (LiN^t^Bu)_2_SiMe_2_ in pentane led to a rapid change in color from ultramarine to dark brown–black. Dark gray particles were observed to precipitate from solution alongside LiCl following the disproportionation of the presumed Co^II^ monokis(diamide) intermediate to form **1** and elemental Co^0^. Workup of the reaction solution afforded **1** as a dark‐brown, almost black crystalline solid that was purified either by recrystallization from pentane at −49 °C or vacuum sublimation (85 °C/10 mTorr). The synthesis scaled well and satisfactory yields (60 % from the ligand) were obtained from 10 mmol batches with ease.

Single‐crystal X‐ray crystallography revealed **1** was a four‐coordinate inorganic spirocycle of *D*_2*d*_ symmetry (Figure 1). With a Yang geometry index value of *τ*_4_=0.75 (Figure 2),^15^ it lies between the two other known four‐coordinate homoleptic Co^IV^ complexes: Hayton's Co(N=C^*t*^Bu_2_)_4_ (*τ*_4_=0.59)^11^ and Co(nor)_4_ (*τ*_4_=0.94).^13^ The average Co−N bond length of 1.84 Å was only slightly longer than in Co(N=C^*t*^Bu_2_)_4_ (1.80 Å), and notably shorter than the Co−C bond in Co(nor)_4_ (1.92 Å). Coordination around each nitrogen was planar, typical of silylamides due to electrostatic repulsion of the polarized Si−N bonds. The ligand planes were arranged almost orthogonally in **1** (*φ*=86.9°), contrasting Hayton's complex which was twisted toward *D*_4*h*_ symmetry (*φ=*57.1°). Both shared the same Co−N coordination sphere, but the increased steric bulk and geometric rigidity of the β‐silyldiamide locked the ligands on **1** into an orthogonal arrangement. This being the case, we expected significant differences in the electronic structure of **1** and Co(N=C^*t*^Bu_2_)_4_.

**Figure 1:**
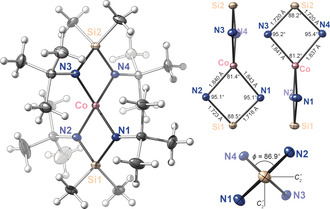
Left: Solid‐state structure of **1** (ellipsoids at 50 % probability).^27^ Top right: Key bond lengths and angles of the two rings with the principle axis oriented vertically; bottom right: ligand plane angle *φ* and the *C*_2_ *′* axes when looking down the principle axis.

**Figure 2:**
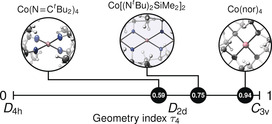
Yang's geometry index *τ*_4_ for known tetracoordinate Co^IV^ complexes.

Room‐temperature magnetic susceptibility measurements of **1** (Gouy method) revealed an effective magnetic moment of *μ*_eff_=(1.8±0.1) B.M. in the solid state, suggesting **1** adopts an unusual low‐spin doublet (*S*=1/2 ) ground state configuration. This was supported by solid‐state EPR spectra which displayed axial spectra consistent with a doublet state (*g_z_=*2.6, *g_x_=g_y_=*2.0, Figure 3). Variable‐temperature EPR studies showed no significant dependence on temperature from 125–300 K (Figure S12). The *g*‐tensor is similar to that of a low‐spin four‐coordinate d^5^ Fe^III^ complex reported by Peters which adopted *C*_3*v*_ symmetry (*g_z_=*2.9, *g_x_=*2.0, *g_y_=*2.0).^16^ The doublet ground state was also supported by density functional theory (DFT) calculations, as we calculated *g_z_=*2.4, *g_x_=g_y_=*2.0 for the doublet state and *g_z_=g_x_=g_y_=*2.0 for the quartet and sextet states.

**Figure 3:**
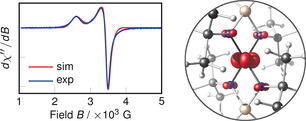
Left: Experimental (blue) and simulated (red) solid‐state X‐band EPR spectra of **1**. Right: Spin density of **1** (def2‐QZVPP//OPBE‐D3(BJ)/def2‐SVP, isosurface value=0.02), highlighting the metal‐based character of the unpaired electron.

We turned to DFT and ab initio coupled‐cluster theory to understand the electronic structure of **1**. Accurate prediction of spin states with DFT is an ongoing challenge because the exact exchange‐correlation functional is still unknown. While mixing a portion of exact Hartree–Fock (HF) exchange into hybrid functionals generally improves performance for closed‐shell molecules, these methods are biased toward high‐spin transition metal configurations.^17^ Nevertheless, all of the pure functionals (BLYP, OLYP, PBE, OPBE) and one hybrid functional (PW6B95) we employed accurately reproduced the experimental structure of **1** when a doublet ground state was considered. The quartet and sextet ground state structures did not: the quartet deviated by exhibiting a smaller ligand interplane angle (*φ*≈72°), and the sextet had elongated Co−N bonds (ca. 1.94 Å). The overall geometric consistency between functionals within different spin states suggested the structures were reasonable, and we proceeded with ab initio calculations for higher accuracy.

Unambiguous assignment of the doublet ground state to **1** was achieved with ab initio semicanonical coupled‐cluster calculations of our best DFT‐optimized structures (DLPNO‐CCSD(T)/def2‐TZVPP//PW6B95/def2‐SVP). This recent method provides comparable performance to canonical CCSD(T), the “gold standard” of quantum chemistry, but with computational cost comparable to DFT.^18^ The doublet was calculated to be 28.1 kcal mol^−1^ and 27.1 kcal mol^−1^ more stable than the quartet and sextet, respectively. Our prior DFT calculations had substantial errors of +22 kcal mol^−1^ and +24 kcal mol^−1^ for the pure and hybrid functionals, respectively. We hope that these benchmarked results of a difficult case may act as reference values in the future.

Only three Co^IV^ complexes with unambiguous doublet ground states have been reported before: Co(nor)_4_, Deng's Co(=Ndipp)_2_(NHC) (dipp=2,6‐diisopropylphenyl, NHC=N‐heterocyclic carbene), and Groysman's Co(OR)_2_(=CPh_2_). But their apparent oxidation states are their only similarity to **1**. Each belongs to a different symmetry point group (*T*_d_ and *C*_2*v*_, respectively); their bonds are distinct from the Co−N bonds in **1**; Co(=Ndipp)_2_(NHC) and Co(OR)_2_(=CPh_2_) are heteroleptic and three coordinate; and the oxidation states in Co(=Ndipp)_2_(NHC) and Co(OR)_2_(=CPh_2_) were tentatively assigned alongside possible Co^III^ resonant forms for which no precedence was found in **1**. Collectively, these dissimilarities made them less useful in explaining the doublet state of **1**. Despite their different ground states, consideration of Hayton's Co(N=C^t^Bu_2_)_4_ was instructive as it shared the same Co–N coordination sphere as **1**.

We constructed a qualitative molecular orbital (MO) diagram of the doublet and quartet configurations of **1** using DFT (Figure 4). Although both states had molecular *D*_2*d*_ symmetry, the molecular orbitals were mostly of lower symmetry in the *D*_2_ point subgroup. Exceptions to this were observed for unpaired electrons: the doublet SOMO (SO=singly occupied) retained full *D*_2*d*_ symmetry, but the quartet SOMOs were of even lower *C*_1_ symmetry. The doublet's MOs were predictable from its symmetry, with the orthogonal ligand orientation stabilizing σ‐ and π‐bonds involving the *b*_2_(*d_xz_*) and *b*_3_(*d_yz_*), and *b*_1_(d${}_{x{}^{2}-y{}^{2}}$ ) DOMOs (DO=doubly occupied), respectively. The corresponding π*‐antibonding *b*_1_(d${}_{x{}^{2}-y{}^{2}}$ ) SOMO showed π‐backbonding in doublet **1**, which was also observed by Mulliken spin‐population analysis (0.52 *e* on Co and 0.52 *e* across four nitrogens). Despite their differences, we found an important parallel between the quartet state of **1** and Hayton's Co(N=C^t^Bu_2_)_4_. The twisted geometry and quartet ground state of Co(N=C^t^Bu_2_)_4_ was due to π‐backbonding of the unpaired electrons to the N=C π‐bonds on the ligands, which was maximized with *D*_2*d*_ symmetry and *S*=3/2 .^11^ Clear π‐backbonding to the N p‐orbital was observed in the three highly distorted SOMOs of quartet **1**, with more than one unpaired electron residing on the nitrogens (1.79 *e* on Co and 1.21 *e* across four nitrogens). Unfortunately, we could not reliably deconvolute the energetic contributions of these π‐systems by energy decomposition analysis (EDA) because of the poor performance of DFT for **1**.

**Figure 4:**
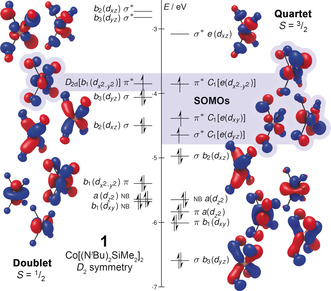
Molecular orbital diagram of **1** in the doublet (left) and quartet (right) spin state from unrestricted natural orbitals (UNOs) obtained with DFT (def2‐QZVPP//OPBE‐D3(BJ)def2‐SVP). DOMOs and LUMOs have *D*_2_ symmetry, while SOMO point groups are explicitly listed. Irreducible representations, participating Co d‐orbitals, and bond type are listed for each. Hydrogen, carbon, and silicon atoms have been omitted for clarity.

Steric bulk clearly played an important role in stabilizing the doublet ground state of **1**, so we optimized the geometries and computed the doublet–quartet energy gaps for less bulky N‐substituents in the same way as for **1** to quantify its effect (Figure 5). All the successfully optimized doublet and quartet geometries had features consistent with those in **1**, and their DFT energy gaps also did not agree with the ab initio results. From the reliable ab initio calculations, we found that reducing the bulk from just *tert*‐butyl to isopropyl made the quartet the most stable electronic configuration by a large margin (Δ*E*=+25 kcal mol^−1^). The SOMOs and spin populations of the quartet states were essentially independent of steric bulk, suggesting that the ligand plane angle *φ* was dependent on the N‐substituent more than on the extent of π‐backbonding. Additionally, the geometric effect of the π‐backbonding was clearly visible in the spin density plots of **1**. These results together demonstrate how the *tert*‐butyl substituent's bulk was necessary to destabilize the otherwise preferred quartet ground state and it enforced the doublet ground state by orthogonal ligand arrangement. Analogues of **1** with less steric bulk are expected to have electronic structures similar to Co(N=C^t^Bu_2_)_4_.

**Figure 5:**
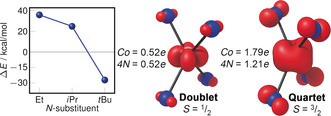
Doublet–quartet energy gaps (DLPNO‐CCSD(T)/def2‐TZVPP//PW6B95‐D3(BJ)/def2‐SVP) for different N‐substituted analogues of **1** (left). Spin density (isosurface at 0.02 a.u.) and Mulliken spin populations of **1** in the doublet (middle) and quartet (right) ground states (def2‐QZVPP//OPBE‐D3(BJ)/def2‐SVP). Hydrogen, carbon, and silicon atoms have been omitted for clarity.

We tested the robustness of **1** to examine its suitability for vapor phase deposition. As prior introduced, ALD is especially good for depositing metastable phases and exotic materials because of its self‐limiting chemical mechanisms and mild operating conditions.^19^ To our knowledge, there have been no reports of either ALD or CVD of Co^IV^‐containing materials because of the lack of Co^IV^ precursors. None of the few previously reported Co^IV^ complexes displayed sufficient thermal stability or volatility. Co(nor)_4_ decomposes at 100 °C and cannot be sublimed, Hayton's Co(N=C^t^Bu_2_)_4_ decomposes in solution at ambient temperature, Deng's Co(=Ndipp)_2_(NHC) undergoes reductive C−H bond activation at 50 °C, while Groysman's Co(OR)_2_(=CPh_2_) is likely nonvolatile due to its high molecular weight and numerous phenyl groups.

We quantitatively evaluated the suitability of **1** as a precursor with respect to its thermal stability and volatility by thermogravimetric analysis (TGA) and differential thermal calorimetry (DSC). When a 10.1 mg sample was heated with a linearly increasing temperature ramp, a clean single‐step mass loss was observed between 150–230 °C leaving behind a small residual mass of 3.2 %, indicating that most of the sample evaporated during the experiment (Figure 6 a). TGA can also be used to efficiently estimate vapor pressure by employing the Langmuir equation, and the temperature at which **1** displays 1 torr of vapor pressure was estimated with a Clausius–Clapeyron model to be (150.4±0.1) °C (Figure 6 b).^20^ DSC revealed the melting point to be 143 °C and the onset of decomposition (defined as 5 % of the maximum of the first exothermic event)^21^ to be 197 °C. The “thermal range” between the 1 torr vapor pressure temperature and the decomposition temperature is often a practical and reliable benchmark of a compound's practicality as an ALD precursor. With a thermal range spanning roughly 47 °C, compound **1** represents a promising precursor candidate for ALD.

**Figure 6:**
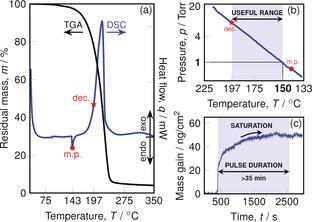
a) TGA of a 10.1 mg sample (black) and DSC (blue) of 0.3 mg of **1**. b) Vapor pressure of **1** and its useful range between the 1 torr and decomposition temperatures. c) Exposure of an alumina‐coated quartz crystal microbalance (QCM) to **1** demonstrating self‐limited adsorption by the saturated mass gain (ng cm^−2^). Experimental details are provided in the Supporting Information for clarity.

To contextualize the performance of **1,** we subjected two recently reported Co^II^ precursors to thermal analysis: namely CoCl_2_(TMEDA)^22^ and Co(DAD)_2_ 23 (DAD=*tert*‐butyldiazadienyl) (see the Supporting Inforamtion). We applied our recently developed “Figure of merit” *σ* that takes key parameters such as thermal range, vapor pressure, and extent of decomposition during TGA into account to quantitatively compare precursors (Table S2).^21^, ^24^ A positive Figure of merit indicates the suitability of a precursor while negative indicates a poor candidate. With *σ*=35, complex **1** ranks between [Co(DAD)_2_] (*σ*=64) and [CoCl_2_(TMEDA)] (*σ*=−2) where the weak performance of the latter originates from its low volatility. Therefore, **1** was not only competitive with successfully applied Co^II^ precursors but, to the best of our knowledge, represents the only known potential Co^IV^ precursor candidate.

The most important characteristic of an ALD precursor is the ability to form a stable self‐limiting monolayer on a surface.^25^ After all reactive surface sites have undergone a reaction with precursor molecules, no further adsorption should occur, allowing conformal and uniform atomic layer deposition to take place during the next self‐limiting reaction.^26^ Employing a quartz crystal microbalance (QCM) in our home‐built ALD tool, we investigated the potential for **1** to fulfill this basic yet critical requirement. The precursor was heated to 135 °C for delivery and the QCM crystal was heated to 150 °C for saturation experiments. Compound **1** demonstrated self‐limiting adsorption on alumina with a mass gain of (49.2±0.3) ng cm^−2^ that was stable over roughly 35 minutes (Figure 6 c). This encouraging initial result motivated us to begin developing ALD processes with **1** in ongoing research.

In summary, a thermally stable, volatile, homoleptic, spirocyclic Co^IV^ bis(β‐silyldiamide) was synthesized in a facile one‐step salt‐metathesis exploiting Co^II^ disproportionation. Experiment and theory confirmed that it exhibits a rare low‐spin d^5^ doublet configuration enforced by the steric bulk and geometric rigidity of its ligands. The remarkable stability of **1** makes it a promising precursor for vapor deposition, and its ability to form a stable self‐limiting monolayer makes it a promising ALD precursor. Beyond this, **1** represents a simple, inexpensive, and accessible starting material for further high‐valent Co^IV^ chemistry to be explored.

## Conflict of interest

The authors declare no conflict of interest.

## Supporting information

As a service to our authors and readers, this journal provides supporting information supplied by the authors. Such materials are peer reviewed and may be re‐organized for online delivery, but are not copy‐edited or typeset. Technical support issues arising from supporting information (other than missing files) should be addressed to the authors.

Supplementary
