# Supplementary material for: A Rare Low‐Spin CoIV Bis(β‐silyldiamide) with High Thermal Stability: Steric Enforcement of a Doublet Configuration
Source: Angew Chem Int Ed Engl. 2020 Jun 8;59(33):14138–42. doi: 10.1002/anie.202001518 (PMC7496428; doi:10.1002/anie.202001518)
Supplement: Supplementary file 1 — Supplementary [file ANIE-59-14138-s001.pdf]

Angewandte Chemie
Eine Zeitschrift der Gesellschaft Deutscher Chemiker

Supporting Information

A Rare Low-Spin Co^{IV} Bis(β-silyldiamide) with High Thermal Stability: Steric Enforcement of a Doublet Configuration**

David Zanders[+], Goran Bačić[+], Dominique Leckie, Oluwadamilola Odegbesan, Jeremy Rawson, Jason D. Masuda, Anjana Devi, and Seán T. Barry**

anie_202001518_sm_miscellaneous_information.pdf

Table of Contents **Page**

1. General methods and procedures 2
2. Synthesis of metal complex Co[(N^{t}Bu)$_2$SiMe$_2$]$_2$ 1 2
3. Thermogravimetric analysis and differential scanning calorimetry of 1 4
4. X-ray crystallography details 4
5. ALD saturation experiments 5
6. Thermal analyses of [CoCl$_2$(TMEDA)] and [Co(DAD)$_2$] and figure of merit 7
7. NMR spectra 11
8. IR spectra 12
9. UV/VIS spectra 13
10. EI-MS data 14
11. EPR Studies 15
12. Computational details 16
13. References 55

1. General methods and procedures

All manipulations were performed under air-free conditions using standard Schlenk techniques or in a N_2-filled (99.998% purity) MBraun Labmaster 130 drybox $CoCl_2$ (anhydrous, 99.9 %), N,N,N′,N′-tetramethylethylenediamine (TMEDA, 99.5 %), dichlorodimethylsilane (98.5 %), ^{n}BuLi (2.0 M in hexanes) and *tert*-butylamine (99.5 %) were purchased from Millipore-Sigma and used as received. Toluene, hexanes, pentane, diethyl ether and THF were ACS reagent-grade, purified by an MBraun Solvent Purifier System, and stored over 4 Å molecular sieves for more than a day before use. Deuterated benzene (C_6D_6) was purchased from Millipore-Sigma and degassed by freeze-pump procedure prior to use. [(N(H)^{t}Bu)$_2$SiMe$_2$] and [(N(Li)^{t}Bu)$_2$SiMe$_2$] were prepared following known literature procedures.[1] [$CoCl_2$(TMEDA)] was synthesized according to a literature described procedure.[2] For a benchmark study on the thermal properties of the title compound **1**, the reference compounds [$CoCl_2$(TMEDA)][3] and [Co(DAD)$_2$][4] (DAD = tert-butyl-diazadienyl) were synthesized following the procedures described in the literature, strictly.

^{1}H Nuclear Magnetic Resonance (NMR) spectra were recorded on a Bruker Avance II 300 MHz instrument. Electron impact mass spectrometry (EI-MS) was performed with a Kratos Concept - Magnetic sector Electron impact mass spectrometer in high resolution mode. Elemental analysis was conducted with a vario micro cube elemental analysis tool (Elementar Analysensysteme) in CHNS analysis mode. Magnetic susceptibility measurements were conducted on a Johnson Mattheys Mark I balance. UV/VIS measurements were obtained on a Varian Cary 3e UV-Visible spectrophotometer. IR spectroscopic measurements were performed on an Agilent Cary 630 FTIR instrument equipped with a diamond attenuated total reflectance unit. EPR spectra were recorded on a Bruker EMXplus X-band EPR spectrometer. Powder X-ray diffractometry (PXRD) of byproduct from the synthesis of spirane **1**, presumed to contain metallic Co, was carried out on a Rigaku Ultima IV diffractometer [X-ray source Cu Kα (λ = 1.54184 Å), 40 kV, 30 mA]. A 100 mg sample of the byproduct was washed with 10 ml of THF for 5 times in a glove box and subsequently dried to remove LiCl. A grey-black powder remained which was carefully filled in a glass capillary. The capillary was sealed with grease, flame sealed and subsequently mounted on a rotatory sample stage. Experiments were conducted in Bragg-Brentano geometry in a 2θ range of (20 - 60)°.

2. Synthesis of metal complex Co[(N^{t}Bu)$_2$SiMe$_2$]$_2$ 1

[$CoCl_2$(TMEDA)] (2.95 g, 12 mmol) was suspended in 50 ml of pentane and stirred for 30 minutes. To this, a solution of [(N(Li)^{t}Bu)$_2$SiMe$_2$] (2.57 g, 12 mmol in 40 ml of pentane) was added *via* syringe within 10 minutes at room temperature (RT). Upon proceeding addition, decolorization of the [$CoCl_2$(TMEDA)] suspension from blue to dark brown-black was observed. The reaction solution was stirred for 16 h at RT. Next to the formation of LiCl by salt metathesis, the formation and sticking of fine disperse particles on the reaction flask walls was observed. The reaction solution was separated from precipitates by filtration and volatiles removed in vacuum. Afterwards, the crude product was further dried in vacuum for 2 h. Purification of the crude product was attempted by 1) crystallization and 2) by sublimation: Purification route 1): Owing to its silazane ligation, compound **1** possesses excellent solubility in commonly used polar and non-polar solvents such as hexanes, pentane, toluene, diethyl ether and THF which aggravates a facile workup by crystallization. Yet, crystallization from a minimal

amount of pentane proved to be repeatably successful presupposed the crystallization ansatz was stored in a freezer at -50 °C for 96 h. Depending on size and thickness, 1.08 g of brown to black crystals were isolated. (Ligand based yield: 40.4 %). While purification by crystallization is possible, it is time consuming and purification according to route 2 as described below is recommended. Purification route 2): The crude product was finely ground and filled in a Schlenk flask with attached sublimation finger. Applying a vacuum of 10 mtorr, sublimation was conducted at 85 °C. Using this method, 1.73 g of $[Co[(N^tBu)_2SiMe_2]_2]$ = [1,3,5,7-tetratertbutyl-2,2,6,6-tetramethyl-1,3,5,7-tetraaza-2,6-disila-4λ^4-cobaltaspiro-[3.3]heptane] were isolated as black crystalline solid in an improved yield of 64.8 % (ligand based).

^{1}H NMR (300 MHz, d^6-benzene): δ (ppm) = -0.32 (s, 12H, (Si(C**H**$_3$)$_2$)), 17.77 (s, 36H, N-C(C**H**$_3$)$_3$). **EA** calcd (%): C 52.25, H 10.52, N 12.19; found (%): C 52.34, H 10.60, N 12.11. **HRMS (EI)** for the molecular ion peak $[M]^+$: Found, m/z = 459.2739, calcd. 459.2750, dev. -0.54%. **EI-MS** (70 eV) m/z (%) [fragment]: 459.3 (46.5 %) $[M]^+$, 444.3 (80.3 %) $[M - CH_3]^+$, 429.2 (36.6 %) $[M - 2x\ CH_3]^+$, 414.2 (100 %) $[M - 3x\ CH_3]^+$, 200.1 (20.8 %) $[L]^+$, 199.6 (67.7 %) $[M - L - 4x - CH_3]^+$, 114.1 (17.9 %) $[L - NC(CH_3)_3 - CH_3]^+$, With: M^+ = Molecular ion peak, L^+ = Diamido ligand peak. **IR** in ATR mode: ν (cm^{-1}) = 2958.6 (m), 2921.3 (m), 2890.4 (m), 2856.6 (m), 1453.3 (m), 1378.7 (w), 1350.3 (m), 1241.3 (m), 1225.8 (m), 1193.6 (s), 1088.4 (s), 1040.8 (w), 1015.6 (w), 963.9 (w), 911.6 (w), 825.8 (s), 756.5 (s), 685.4 (s), 663.1 (s). **UV/VIS** (Toluene): λ_{max} (nm) = 320.1. **Magnetic moment:** μ_{eff} (μB) = 1.8 ± 0.1.

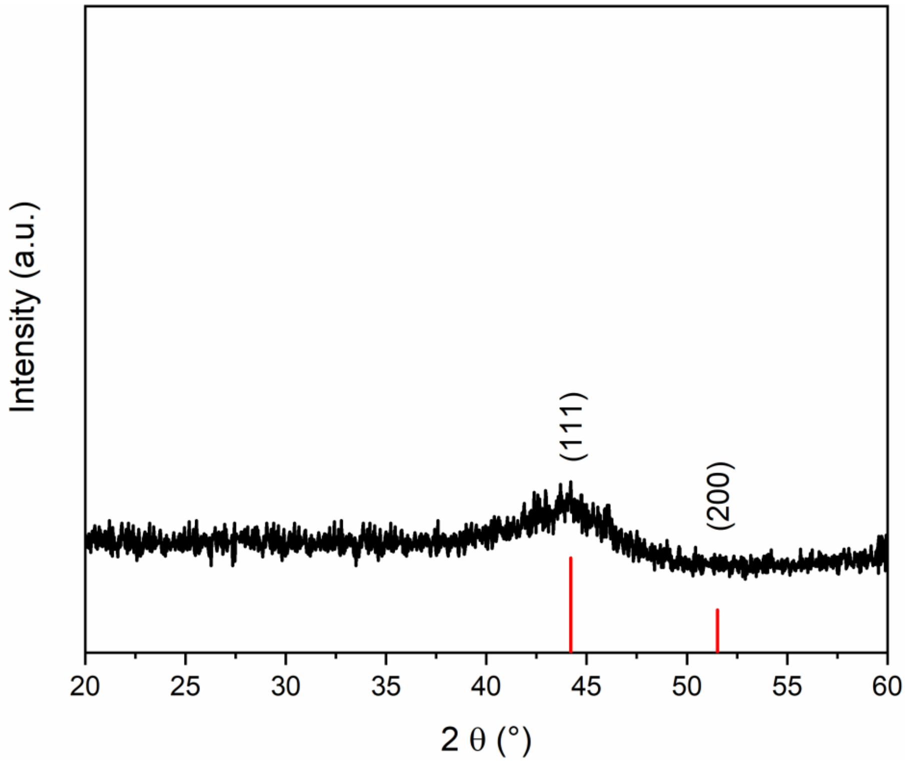

Figure S 1: PXRD of Co particles which precipitated in the course of the synthesis of spirane **1**. As indicated by a broad reflection with a maximum intensity at 2θ = 44.0 ° which can be assigned to the (111) reflection of Co metal in its cubic phase,[5] the precipitate is poorly nanocrystalline.

3. Thermogravimetric analysis and differential scanning calorimetry of 1

TGA was performed on Pt pans with a TA Instruments Q50 housed in an MBraun Labmaster 130 dry-box filled with nitrogen gas (99.998 % purity). Pt pans were cleaned by sequential ultrasonication in dilute nitric acid (~3 N), water, then 2-propanol. They were then heated until red hot by a propane torch flame in air to remove any remaining impurities. Ramp experiments were performed under a flow of ultrapure nitrogen (99.999% purity, 6 sccm) at 10 °C min^{-1} to a maximum temperature of 500°C. The Langmuir vapor pressure equation for compound **1** was derived from TGA data (10 mg run) using a previously reported method,[6] employing *bis*(2,2,6,6-tetramethyl-3,5-heptanedionato)copper(II) as the calibrant.[7] The temperature at which a compound provides a vapor pressure of 1 Torr is referred to as T_V.
DSC experiments were performed with a TA Instruments Q10 instrument. DSC samples with mass loadings of **1** of (0.30 ± 0.03) mg were sealed in aluminum pans, inside a glovebox prior to analysis. Unless otherwise stated all samples were heated to 400 ºC with a ramp rate of 10 ºC min^{-1}, using nitrogen (99.998 % purity) as the purge gas. The onset of decomposition (T_D) was defined as a 5% increase in exothermic heat flow from the beginning of the exothermic event. DSC experiments for **1** with approximately identical mass loadings were repeated three times to ensure the validity of the recorded data.

4. X-ray crystallography details

Crystals of compound **1** suitable for Single Crystal X-ray Diffraction (SC-XRD) analysis were grown from a cooled toluene solution of prior sublimed material kept at - 50 °C in a freezer.
The crystal chosen was attached to the tip of a MicroLoop with Paratone-N oil. Measurements were made on a Bruker D8 VENTURE diffractometer equipped with a PHOTON III CMOS detector using monochromated Mo Kα radiation (λ = 0.71073 Å) from an Incoatec micro-focus sealed tube at 100 K.[8] The initial orientation and unit cell were indexed using a least-squares analysis of the reflections collected from a complete 360° phi-scan with 1° per frame. For data collection, a strategy was calculated to maximize data completeness and multiplicity, in a reasonable amount of time, and then implemented using the Bruker Apex 3 software suite.[8] The crystal to detector distance was set to 4 cm. Data collection, unit cell refinement, data processing and multi-scan absorption correction were applied using the APEX3 software package. The structures were solved using SHELXT[9] and all non-hydrogen atoms were refined anisotropically with SHELXL[10] using a combination of shelXle[11] and OLEX2[12] graphical user interfaces. Unless otherwise noted, all hydrogen atom positions were idealized and ride on the atom to which they were attached. The final refinement included anisotropic displacement factors on all non- hydrogen atoms. Figures were generated in Diamond (Version 3.2k).[13] CSD entry 1977041 contains the supplementary crystallographic data for complex **1**. These data can be obtained free of charge from the Cambridge Crystallographic Database Centre via https://summary.ccdc.cam.ac.uk/structure-summary-form.

Table S 1: Selected crystallographic and data collection parameters for **1**.

**1,3,5,7-tetra-tertbutyl-2,2,6,6-tetramethyl-1,3,5,7-tetraaza-2,6-disila-4λ⁴-cobalta(IV)spiro-[3.3]heptane**	
**Formula**	$C_{20}H_{48}CoN_4Si_2$
**M (g mol^{-1})**	459.73
**Temperature (K)**	100
**Wavelength (Å)**	0.71073
**Crystal system**	Tetragonal
**Space group**	$I\ 4_1/a$
a **(Å)**	18.1088(3)
b **(Å)**	18.1088(3)
c **(Å)**	32.6789(7)
V **(Å^3)**	10716.3(4)
Z	16
**ρ_{calc} (g cm^{-3})**	1.140
**μ (mm^{-1})**	0.742
***F*(000)**	4016.0
**Crystal size (mm^3)**	0.35 × 0.33 × 0.25
**$2\theta_{min}$ - $2\theta_{max}$**	2.96 - 33.12
**Completeness to 2θ**	0.997
**Reflections: total/independent**	10188/9185
**GOF of F^2**	1.084
**R_1, wR_2 [$I < 2\sigma(I)$]**	0.0231, 0.0592
**R_1, wR_2 (all data)**	0.0285, 0.0635

5. ALD saturation experiments

All ALD saturation experiments were conducted on a home-built low-pressure hot-walled ALD instrument equipped with a quartz crystal microbalance (QCM) (COLNATEC, EON-LT) with an integrated temperature reader. The QCM crystal (PhillipTech, 6 MHz, 14 mm) was mounted on a QCM stage located on a holder that was inserted into the horizontal flow reactor chamber 2 hours prior to an experiment run to allow equilibration of temperature and frequency of the crystal. Experiments were conducted at a temperature of 150 °C. For each run 1 g of compound **1** was loaded into a stainless steel bubbler and heated to 135 °C. During the exposure tests a nitrogen (99.998 %) carrier gas flow of 50 sccm was directed through the bubbler system to ease precursor transport. In initial runs, the bare QCM crystal surface. i.e. an Au surface, was exposed to vapor of **1**. As no significant frequency change of the microbalance indicating adsorption occurred even 5 minutes after the beginning of the pulse, we decided to alternate the QCM surface seeking to create a more adsorption-friendly environment for **1**. We decided to coat the QCM crystals with a thin layer of Al_2O_3 employing the universally applicable trimethyl aluminum (TMA) and H_2O process for all subsequent experiments. For the surface pre-treatment, the metal-organic precursor and the co-reactant were fed into the reactor system from stainless steel cartridges by simple vapor draw owing to their high volatility. The pulse sequence was TMA: 0.1 s - N_2 purge (50 sccm): 10 s - H_2O: 0.1 s - N_2 purge (50 sccm): 10 s and 100

cycles were applied typically if not noted differently. After the deposition of Al_2O_3 layers the QCM crystal was allowed to equilibrate for 30 minutes before a saturation experiment with **1** was started. Saturation experiments were then conducted with long exposure times of up to 35 minutes to demonstrate the self-limiting character of adsorption. The adsorbed mass per cm^2 (Δm) was derived from the Sauerbrey-equation,[14] which can be rewritten as:

$$\Delta m = \frac{\Delta f}{S} \quad (\mathbf{1})$$

with Δf being the frequency shift and S being the sensitivity factor of the employed crystal with the quartz density $\rho_Q = 2.64\ g\ cm^{-3}$,the surface area of the quartz crystal A and the shear wave velocity of quartz (AT-cut) $v_Q = 3.34 \times 10^{-5}\ cm\ s^{-1}$

$$S = \frac{2 * f_0^2 * A}{\rho_Q * v_Q} \quad (\mathbf{2})$$

6. Thermal analyses of [$CoCl_2$(TMEDA)] and [Co(DAD)$_2$] and figure of merit

TGA and DSC experiments on [$CoCl_2$(TMEDA)] and [Co(DAD)$_2$] were performed on the same instruments and the identical methodology priorly described in section 3. Figures S 2 - S 4 illustrate the volatilization and decomposition behavior of [$CoCl_2$(TMEDA)] and figures S 5 - S 7 the ones of [Co(DAD)$_2$].

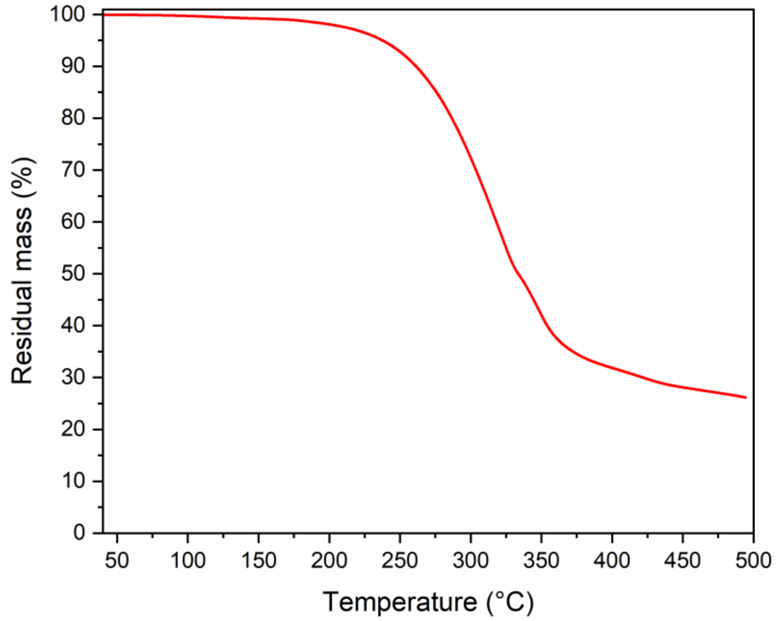

Figure S 2: Thermogravimetric analysis of a 10.3 mg sample of [$CoCl_2$(TMEDA)]. The red curve indicates the weight loss with a residual mass of around 26.2 %.

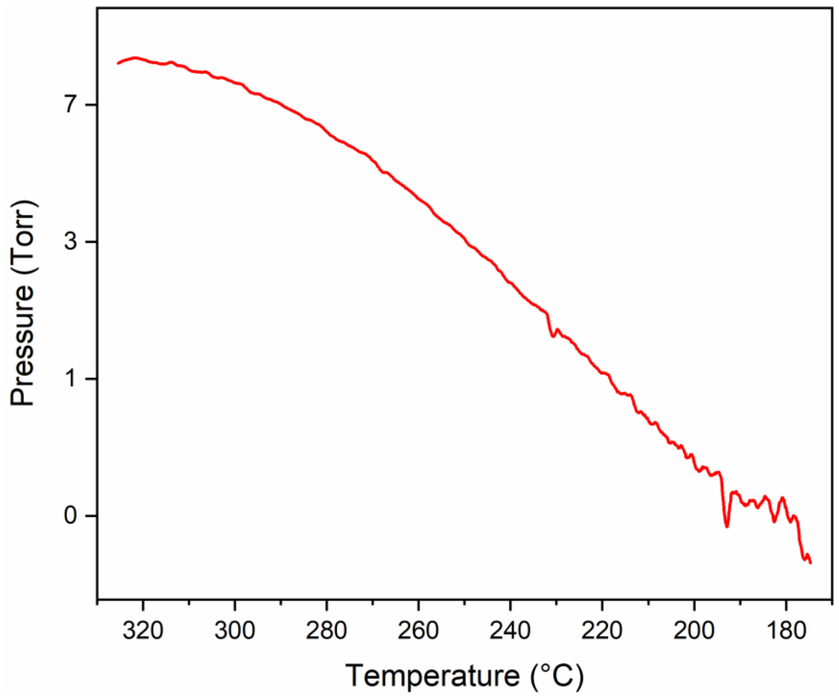

Figure S 3: Vapor pressure - temperature correlation for [$CoCl_2$(TMEDA)] modeled according to the Langmuir equation estimated from TGA (Figure S 2). T_V = (215.9 ± 0.6) °C.

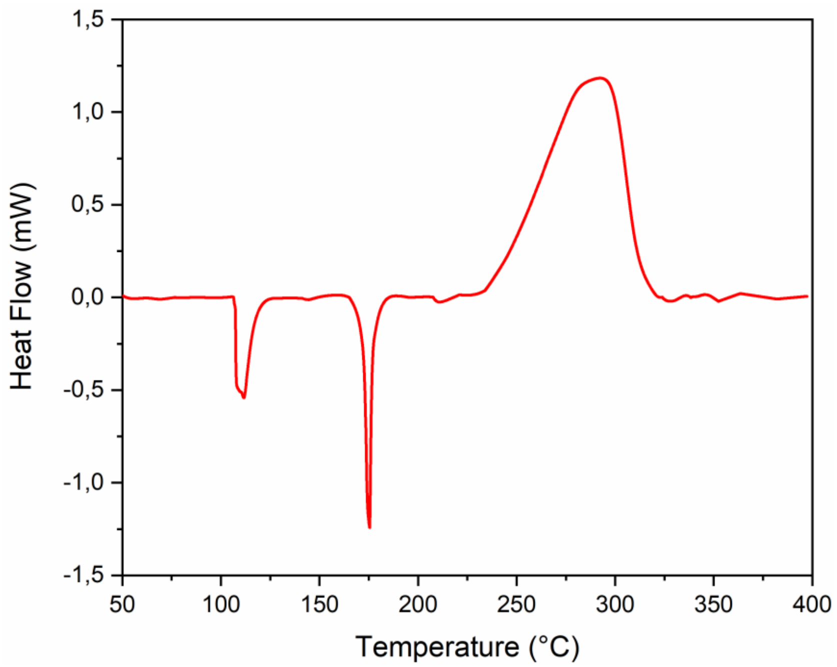

Figure S 4: Differential scanning calorimetry plot of [$CoCl_2$(TMEDA)] with a mass loading of 0.325 mg. The onset of decomposition was determined to be T_D = 235.2 °C and the melting point indicated by an endothermal drop of the heat flow at around 174 °C.

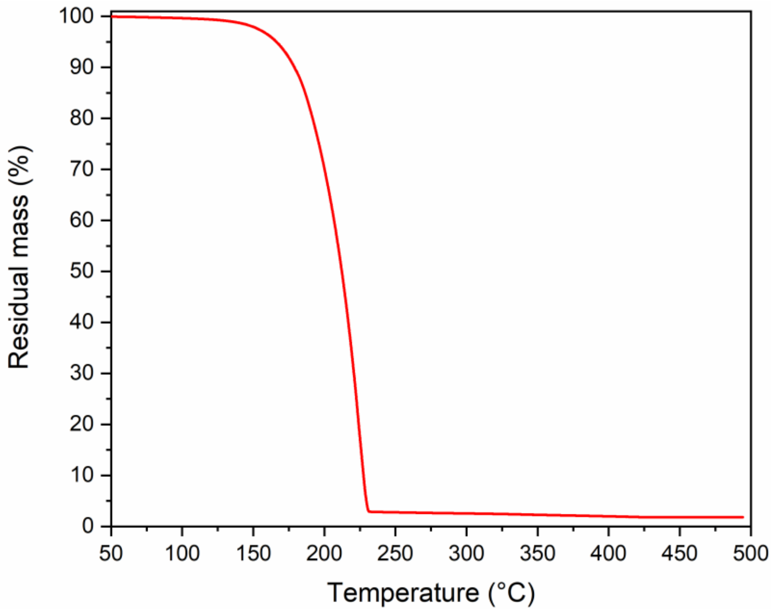

Figure S 5: Thermogravimetric analysis of a 11.0 mg sample of [Co(DAD)$_2$]. The red curve indicates the weight loss with a residual mass of around 1.8 %.

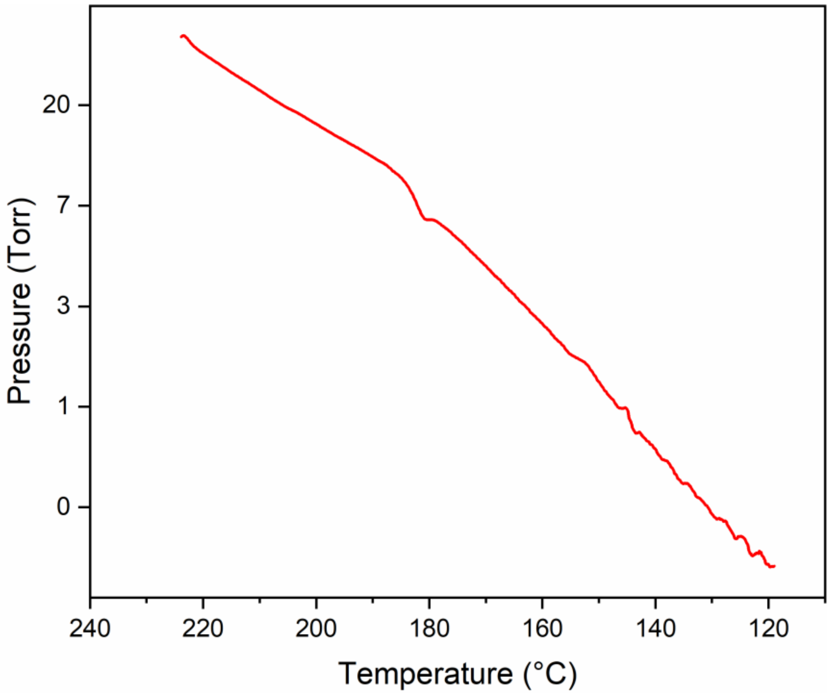

Figure S 6: Vapor pressure - temperature correlation for [Co(DAD)$_2$] modeled according to the Langmuir equation estimated from TGA (Figure S 5). T_V = (145.8 ± 0.3) °C.

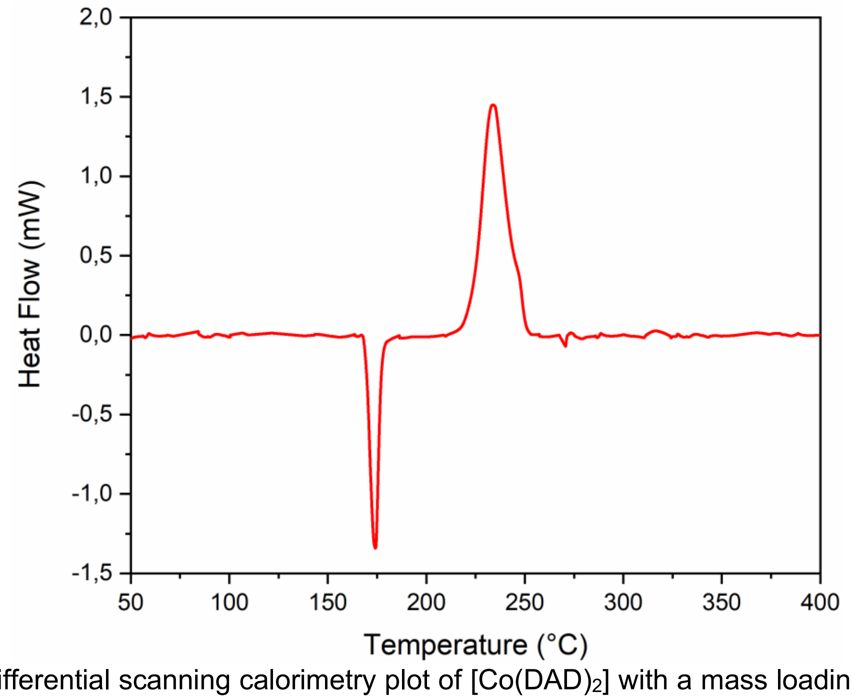

Figure S 7: Differential scanning calorimetry plot of [Co(DAD)$_2$] with a mass loading of 0.347 mg. The onset of decomposition was determined to be T_D = 218.7 °C and the melting point indicated by an endothermal drop of the heat flow at around 175 °C.

Acquired TGA and DSC data and analyses can be rationalized using the figure of merit, σ, whose concept our group applied for the evaluation of a family of molybdenum and gold precursors recently.[15] The figure of merit equation is presented below (equation 3). The first term represents the thermal range between the onset of decomposition (T_D) and the temperature at which a compound provides a vapor pressure of 1 Torr (T_V). In cases where the onset of decomposition is at a lower temperature than the 1 Torr temperature, a poor longer-term thermal stability at temperatures necessary for a compound to be evaporated sufficiently for ALD is indicated. The second term

compares the residual mass in TGA to the weight percent on cobalt in the compound. The hereby calculated σ values are a statement on the usability of a precursor candidate in ALD that makes allowance for thermal stability especially. They are expressed in arbitrary units. Thus, justice is done to the fact that precursors are typically kept at around their 1 Torr vapor pressure temperature for time spans of several hours to several days, depending on scale and application of an ALD process. The figure of merit calculations for **1** (equation 4), [$CoCl_2$(TMEDA)] (equation 5) and [Co(DAD)$_2$] (equation 6) as well as table 2 summarizing the underlying thermal data are given below.

$$\sigma = (T_D - T_V) \cdot \left(1 - \frac{\%m_{res}}{\%m_{Co}}\right) \quad (\mathbf{3})$$

$$\sigma_{[\mathbf{1}]} = (197.0 - 150.4) \cdot \left(1 - \frac{3.2}{12.8}\right) = 35.0 \quad (\mathbf{4})$$

$$\sigma_{[CoCl2(TMEDA)]} = (235.2 - 215.9) \cdot \left(1 - \frac{26.2}{24.0}\right) = -1.8 \quad (\mathbf{5})$$

$$\sigma_{[Co(DAD)2]} = (218.7 - 145.8) \cdot \left(1 - \frac{1.8}{14.9}\right) = 64.1 \quad (\mathbf{6})$$

Table S 2: Thermal data for the figure of merit calculation of **1**, [$CoCl_2$(TMEDA)], [Co(DAD)$_2$]

Precursor	T_D (°C)	T_V (°C)	ΔT (°C)	Residual mass (%)	σ value
**[1]**	197.0	150.4	46.6	3.2	35.0
**[$CoCl_2$(TMEDA)]**	235.2	215.9	19.3	26.2	-1.8
**[Co(DAD)$_2$]**	218.7	145.8	72.9	1.8	64.1

7. NMR spectra

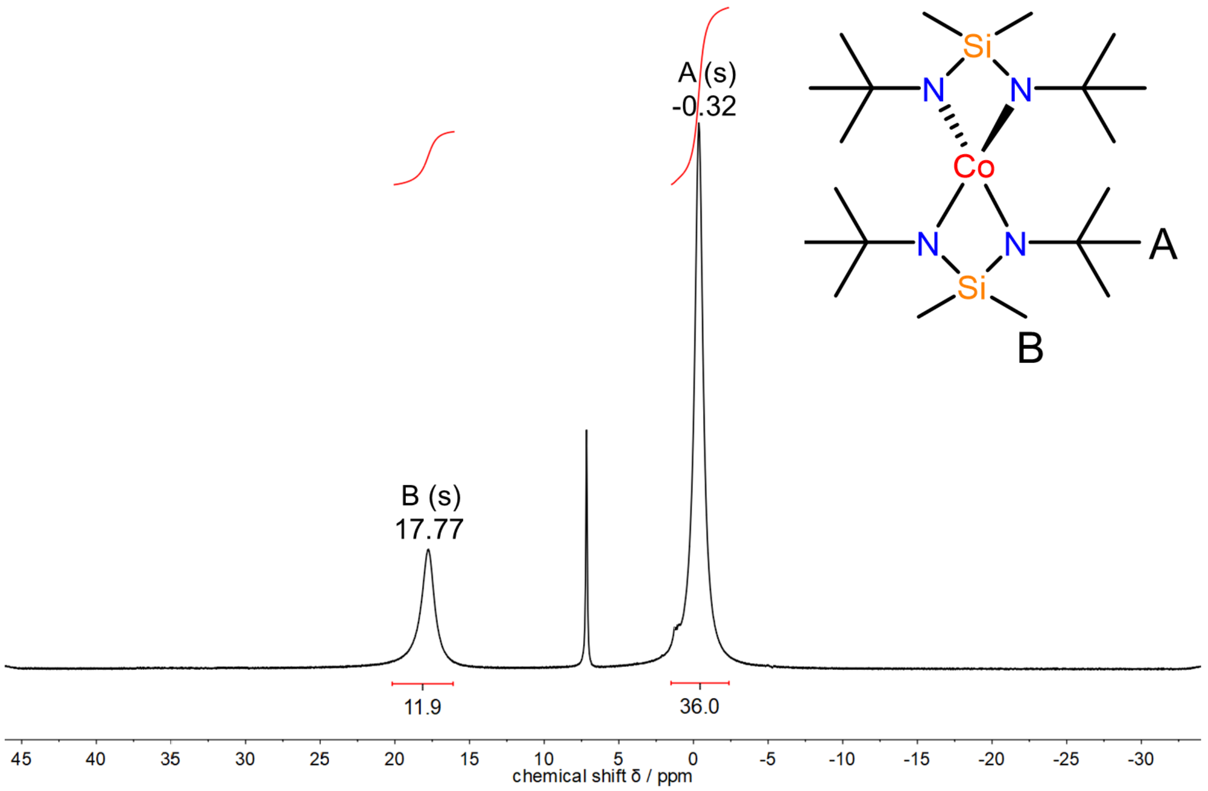

Figure S 8: ^{1}H-NMR of [Co[(N^{t}Bu)$_2$SiMe$_2$]$_2$] **1** in d^{6}-benzene. A first broad singlet (s) with an integral of 36 can be observed at δ = -0.32 ppm correlating to the CH_3 protons of the tertbutyl moieties attached to the amido nitrogen atoms. Another singlet (s) appears in the high-field region at δ = 17.77 ppm representing the CH_3 protons of the methyl groups of the silazane backbone silyl moiety possessing an integral of 12.

8. IR spectra

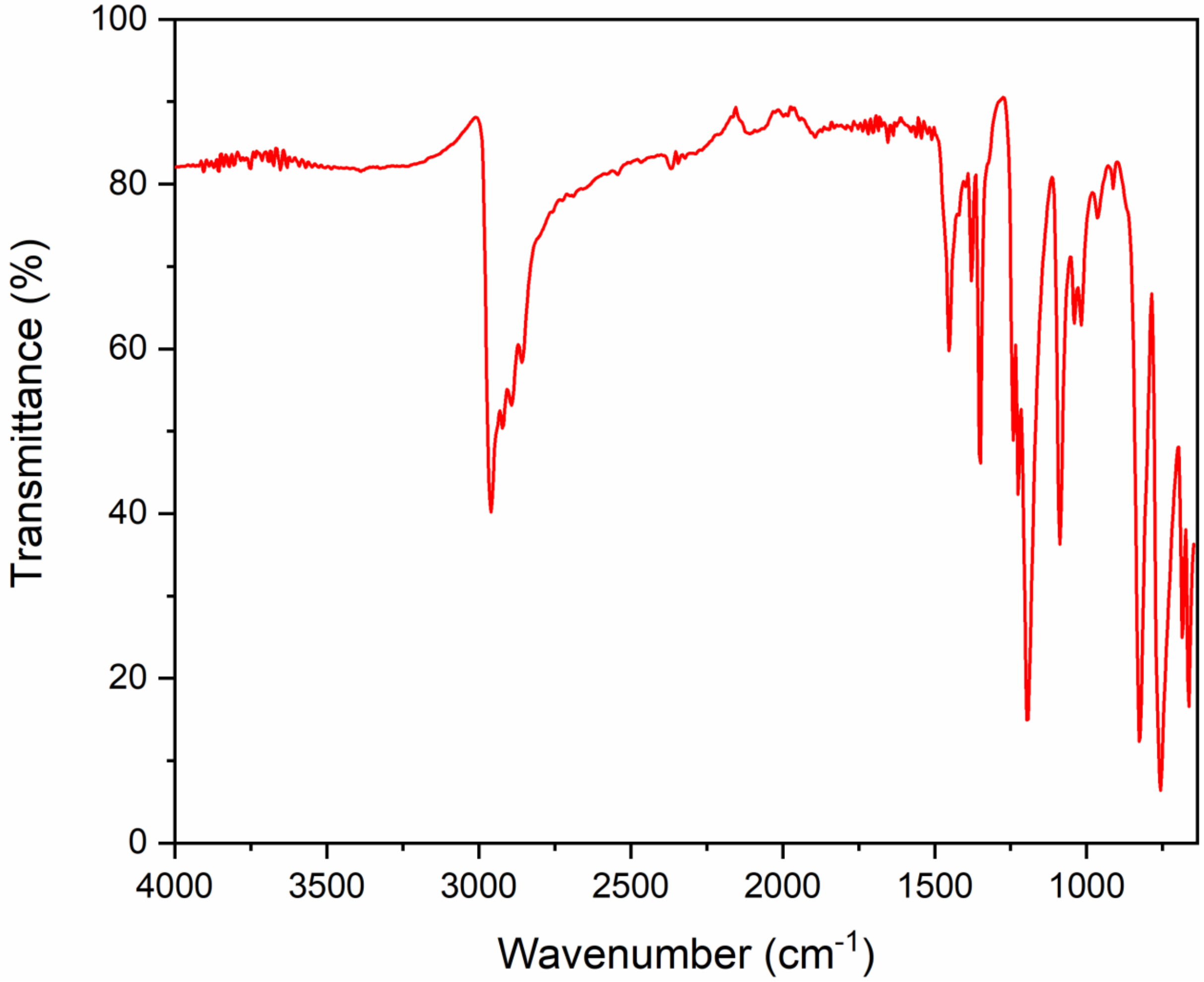

Figure S 9: IR spectrum of **1** revealing a series of signals in the region of (2960 - 2860) cm^{-1} corresponding to CH_3 stretching vibrations. At lower wavenumbers (1454 - 1349) cm^{-1} signals assignable to C-CH_3 bending vibrations and Si-CH_3 bending vibrations (1197 cm^{-1} and higher wavenumber shoulder) can be observed. A pronounced signal at 1088 cm^{-1} indicates N-C stretching vibrations.

9. UV/VIS spectra

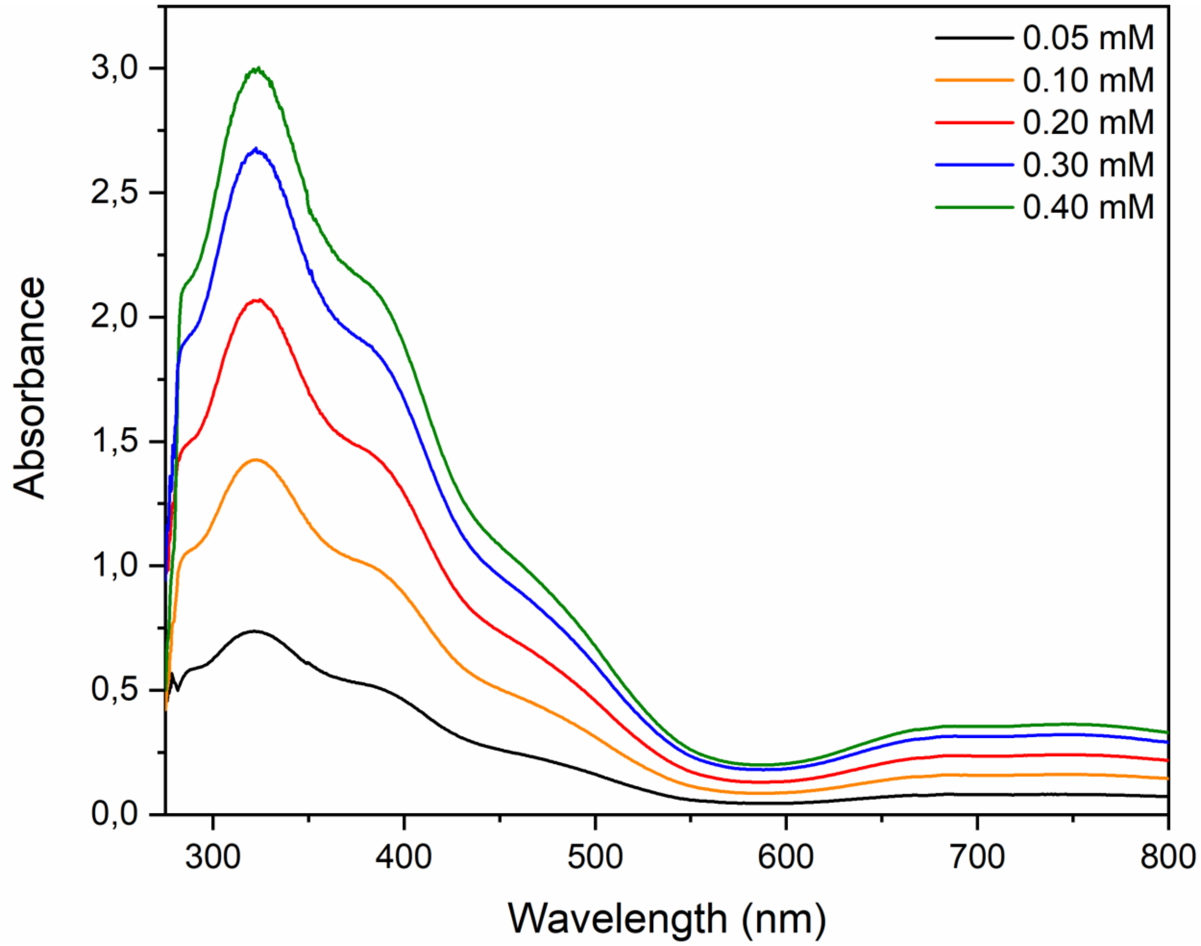

Figure S 10: UV/VIS spectra of solutions of compound **1** in concentrations ranging from 0.05 mM - 0.40 mM demonstrating pronounced absorbance in the UV region with absorbance maxima around 320 nm (31.250 cm^{-1}). While this feature can be assigned to charge transfer (CT) between ligand and metal center clearly, a higher wavelength feature around 400 nm (25.000 cm^{-1}) might originate from CT as well. The shoulder at roughly 475 nm (21,000 cm^{-1}) is indicative of a forbidden *d-d* transition. It has to be noted that the Lambert Beer law loses its validity for concentrations exceeding 0.10 mM.

10. EI-MS data

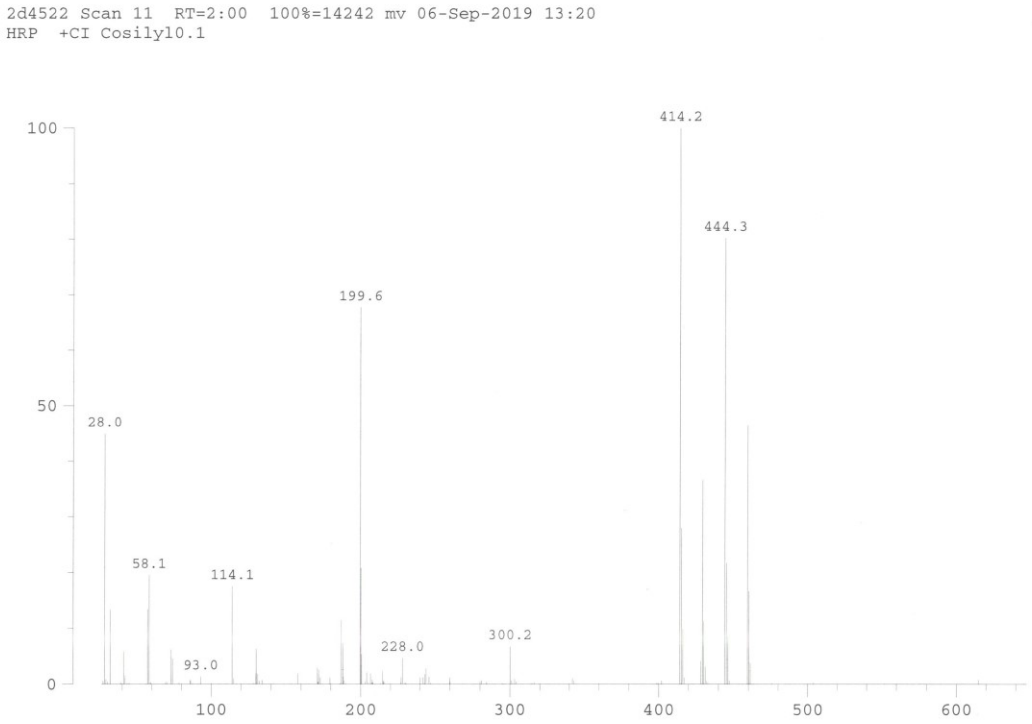

Figure S 11: Original print-out of the EI-MS measurement conducted on a sample of **1** revealing a signal at 459.27 m/z to be the molecular ion peak M^+.

11. EPR Studies

Solid state EPR spectra on polycrystalline samples of **1** were recorded on a Bruker EMXplus X-band EPR spectrometer equipped with a liquid nitrogen flow cryostat between 125 and 300 K. EPR spectra showed no significant temperature dependence in terms of features over this temperature range, although there was the anticipated enhancement in signal intensity at lower temperatures due to the improved population difference between states, Fig. S 7. A simulation of the room temperature data used PIP through the PIP4WIN GUI (see main manuscript).

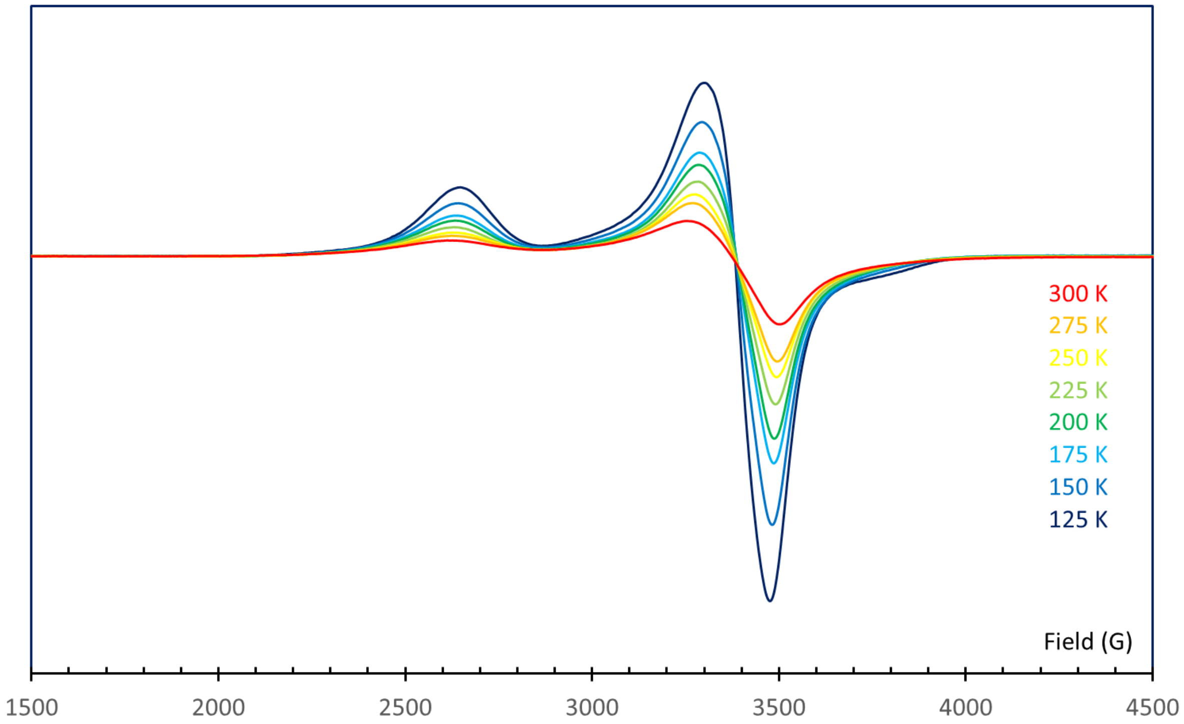

Figure S 12: Variable temperature solid state X-band EPR spectrum of **1** from 125 - 300 K.

12. Computational details

Initial density functional theory (DFT) calculations for 1 and [Co(nor)$_4$] were performed with Gaussian 16 Revision C.01 (G16).[16] DFT was used to optimize the geometries of 1 in its doublet, quartet, and sextet spin states. Initial geometries were taken from the solid-state structures (XRD, vide supra, and [17], respectively). Then, we optimized the structures using the open-shell unrestricted Perdew-Burke-Ernzerhof (PBE) exchange and correlation functional[18] (*uPBEPBE* keyword in G16), Ahlrichs's double-zeta quality split valence basis set def2-SVP on all atoms,[19] neutral charge, and ultrafine integral grid. These were then used as starting geometries and optimized with the APFD (only 1),[20] B97-D3(BJ),[21] PW6B95-D3(BJ) hybrid,[22] and wB97X-D2[23] range-separated functionals using the same prior other settings. All geometries were confirmed to be minima on the potential energy surface (PES) by ensuring they had no imaginary frequency modes. Finally, we performed single point energy calculations using Ahlrichs's quadruple-zeta with polarization basis set def2-QZVPP to approach the basis set limit and minimize any basis set superposition error (BSSE).

EPR g-tensor calculations were performed with ORCA v4.2.1[24] using open-shell unrestricted DFT for the doublet, quartet, and sextet spin states of **1** (def2-QZVPP//PW6B95-D3(BJ)/def2-SVP) using geometries taken from the previous G16 calculations (vide supra). Since there were no heavy atoms, only the isotropic and dipolar parts of the hyperfine coupling (HFC, *aiso* and *adip* keywords) were considered for all nuclei in the g-tensor calculations. Additionally, a benchmark calculation for the doublet state including spin-orbit coupling (*aorb* keyword) was performed which gave identical g-tensor values as the calculations without it.

The poor ability of DFT to predict the spin-state of **1** prompted us to explore other functionals and more accurate post-HF methods with ORCA v4.2.1. We explored the effect of the added fraction of Hartree-Fock (HF) exchange in hybrid functionals by employing their corresponding "pure" density functionals that omit it. Thus, complex **1** in the doublet and quartet ground state was reoptimized with the def2-SVP basis set and the following "pure" generalized-gradient approximation (GGA) functionals: PBE exchange and correlation,[18] Becke exchange[25] with Lee-Yang-Parr correlation (BLYP),[26] and Handy-Cohen optimized exchange (OPTX) with LYP (OLYP),[27] and with PBE correlation (OPBE)[27]. The resolution of identity for Coulomb integrals and COSX numerical integration for HF exchange (RIJCOSX) approximation[28] with a grid size of *GridX7* was used to speed up the UHF step. Convergence criteria were set to *VeryTightSCF* and *VeryTightOpt* with *Grid5* and *FinalGrid7* integral grid sizes. Numerical frequency calculations were performed to confirm they were minima on the PES and to avoid a known bug in ORCA involving some functionals, RIJCOSX, and analytical Hessians. Optimizations were followed by single-point calculations with a large basis set (def2-QZVPP) for improved accuracy. The pure functionals all correctly identified the doublet state as the preferred configuration, and the consistently excellent performance of the OPBE functional in the literature[29] and its simple implementation in ORCA encouraged us to trust its results for molecular orbital analysis. Unrestricted natural orbitals (UNOs) were generated with the *UNO* keyword in ORCA.

Less sterically encumbered *N*-substituted analogues of **1** were also investigated with ORCA v4.2.1. The structures were optimized using unrestricted DFT (def2-QZVPP//PW6B95-D3(BJ)/def2-SVP) with the RIJCOSX approximation with the substituents R = CH_3, CH_2CH_3, and $CH(CH_3)_2$. Starting geometries were obtained by modifying the structure of **1** with Avogadro v1.4 by replacing CH_3 moieties with H on the *tert*-butyl groups and then reoptimizing as with **1** above. Default convergence

criteria for the post-HF calculations were used except for the doublet $CH(CH_3)_2$-substituted analogue which required a lower tolerance on the residual for the DLPNO-CCSD step to converge (*STol* was set to 10^{-5}, ORCA default is 10^{-6}).

Domain based local pair natural orbital coupled cluster calculations with singles, doubles, and perturbative triples [DLPNO-CCSD(T)][30] were performed with ORCA v4.2.1 using an unrestricted Hartree-Fock (UHF) reference wavefunction (*VeryTightSCF* convergence) and the def2-TZVPP basis set. This method was applied using geometries obtained from DFT (PW6B95-D3(BJ)/def2-SVP). The reference wavefunctions were first calculated using a double-ζ (def2-SVP) basis set then used as a starting point for the triple-ζ reference calculations. Quasi-restricted orbitals (QROs) were used to eliminate spin contamination.[31] The def2-TZVPP basis set was used on all atoms for the final DLPNO-CCSD(T) calculations. Default convergence criteria for the post-HF calculations were used. Wavefunction analysis was performed with Multiwfn v3.7[32] running on one of the author's personal computer using wavefunction data from G16 and ORCA v4.2.1. It was used to calculate the spin density of **1** and $Co(nor)_4$; perform Mayer bond order (MBO),[33] Bader's quantum topology of atoms-in-molecules (QTAIM),[34] and Johnson's non-covalent interaction (NCI) analysis;[35] and generate cube files for interpretation and visualization.

All Gaussian and ORCA calculations were performed on the Cedar cluster maintained by Compute Canada and housed at Simon Fraser University.

Supplemental Computational Results & Discussion

The relatively short Co-Si distance in **1** (2.63 Å) caught our attention as it raised the question of whether any kind of Co—Si bond exists. Naturally, the answer depends on which of the definition of "bond" is chosen. Mayer bond order (MBO) is a popular and powerful tool to investigate bonding, but for **1** it indicated a Co—Si bond of order ~0.6 and also predicted unintuitive Co—C bonds to the *tert*-butyl moieties on the order of ~0.3. As expected, neither the canonical nor localized molecular orbitals showed any bonding interactions between Co and Si nor C, which suggested the "bonds" do not exist. Additionally, we could find no evidence of hyperconjuction between Si—C bonds and the nitrogens or metal center, nor any Si 3*d* orbital involvement whatsoever. Nothing resembling the hyperconjugation of the *β*-Si effect in organic systems could be found in **1**, and the remarkable stability of this complex appeared to be coincidental with the *β*-position of the Si. The average MBO for the four Co—N bonds was around ~1.0, which accurately reflects the single covalent bonds expected between the metal center and the mono-anionic *σ*-bonding N atoms. The difference in Co—N bonding in **1** and Deng's carbene-stabilized Co(IV) bis imide complex is illustrated.[36] While the average Co—N bond order by MBO analysis was ~1.4 in latter and made an accurate determination of the physical oxidation state at the Co center not straightforward. This comes with little surprise as transition metal imido complexes have been shown to possess diverse M—N bonding modes with varying bond orders originating from σ- and π-bonding contributions.[37] Contrasting this, there is little ambiguity on the +IV oxidation state of the Co center in **1** that is solely surrounded by strongly sigma-donating N species. The erroneous assignment of bonds by MBO are likely due to its use of the overlap matrix *S* and the proximity of the Co, Si and C atoms. Indeed, there was a positive correlation between the number of basis functions N and the calculated MBOs of Co with C and Si (Figure S 13). Bader's atoms-in-molecules (AIM) theory provides a clear, physically justified definition of the chemical bond as a saddlepoint in the topology of the electron density. There were no (+3, −1) bond-critical points (BCPs) between Co and Si nor C, nor was there any significant charge accumulation between

them. This was further supported by Laplacian bond order (LBO) calculations. In conclusion, there were no Co—Si nor Co—C bonds in **1**.

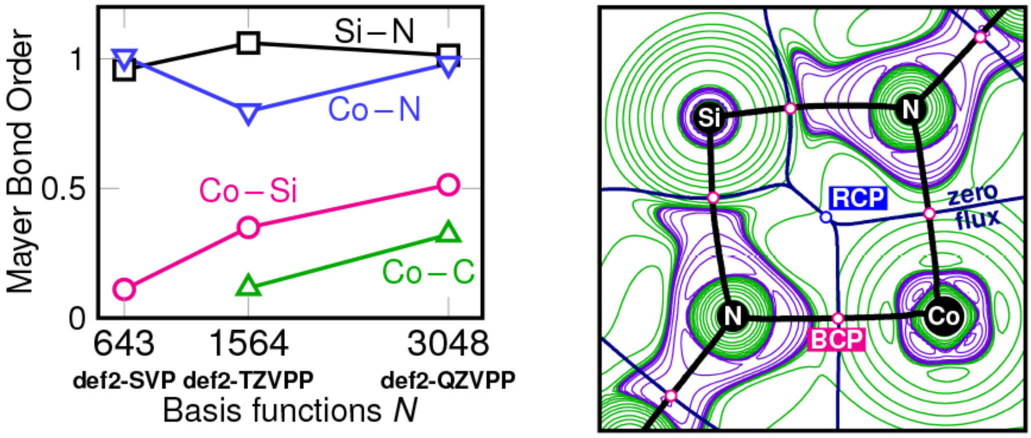

Figure S 13: Mayer bond orders (MBOs) as a function of number of basis functions N (left), and contour map of $\nabla^2\rho$ showing bond critical points (BCPs), the ring critical point (RCP), and the interatomic basins within one heterocycle of **1** (right).

Dispersion interactions were recently shown to significantly stabilize [Co(nor)$_4$],[38] and we suspected they played a role in the structure of **1**. Johnson et al. described a method to visualize non-covalent interactions (NCIs) in real space, showing the attractive and repulsive interactions occurring within and between molecules.[35] NCI analysis of [Co(nor)$_4$] and **1** (Figure S 8) could not identify any obvious non-covalent interaction responsible for their low-spin states, and appeal to their electronic structure was required.

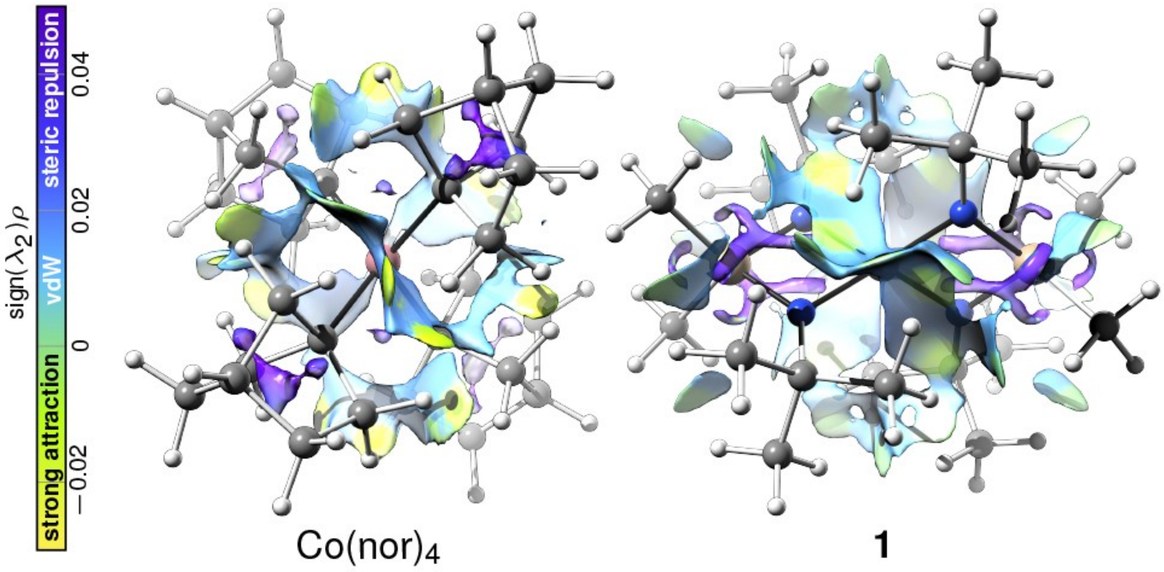

Figure S 14: Non-covalent interactions (NCIs) at work in [Co(nor)4] (left) and 1 (right). The surfaces are at an isovalue of 0.5 a.u. and colored by the value of sign(λ2)ρ from –0.03 (yellow/green, attractive), through ca. zero (cyan, van der Waals interaction) to 0.05 a.u. (indigo/purple, steric repulsion).

Table S 3. Self-consistent field energies E_{SCF} in Hartrees of complex **1**.

	**Functional** Type % HF Exchange	Basis set	E_{SCF} / Hartree $S = {}^1/_2$	$S = {}^3/_2$	$S = {}^5/_2$
Gaussian 16 Rev. C.01	**APFD** Hybrid GGA 23%	def2-SVP	-2969.819569	-2969.811802	-2969.819569
		def2-TZVPP	-2971.312415	--*	-2971.301786
		def2-QZVPP	-2971.419752	-2971.411599	-2971.408979
	**B97-D3(BJ)** Hybrid GGA 19.43%	def2-SVP	-2970.866129	-2970.861605	-2970.847398
		def2-TZVPP	-2972.381326	--	-2972.361194
		def2-QZVPP	-2972.488610	-2972.483324	†
	**PW6B95-D3(BJ)** Hybrid Meta GGA 28%	def2-SVP	-2973.433168	-2973.427461	-2973.436711
		def2-TZVPP	-2974.968253	--	-2974.969190
		def2-QZVPP	-2975.079672	-2975.073297	-2975.080484
	**ωB97X-D2** Range-separated Hybrid GGA 22% Short, 100% Long	def2-SVP	-2970.619211	-2970.614014	-2970.619955
		def2-TZVPP	-2972.122184	--	-2972.086479
		def2-QZVPP	-2972.232707	-2972.226774	-2972.228145
ORCA v4.2.1	**BLYP-D3(BJ)** Pure GGA	def2-SVP	-2970.528265	-2970.517006	—‡
		def2-QZVPP	-2972.236084	-2972.224022	—
	**PBE-D3(BJ)** Pure GGA	def2-SVP	-2969.077889	-2969.067777	—
		def2-QZVPP	-2970.710574	-2970.699537	—
	**OLYP-D3(BJ)** Pure GGA	def2-SVP	-2971.16159	-2971.154074	—
		def2-QZVPP	-2972.756551	-2972.748251	—
	**OPBE-D3(BJ)** Pure GGA	def2-SVP	-2971.11986	-2971.112070	—
		def2-QZVPP	-2972.669443	-2972.660646	—
	**DLPNO-CCSD(T)¶** Post-HF	def2-TZVPP	-2968.506176	-2968.461349	-2968.462967

* Basis set convergence issues were noticed for other calculations with triple-ζ valence quality basis sets, so energies were only calculated at quadruple-ζ quality for S = 3/2 spin states.
† The SCF step failed to converge even after thousands of cycles and was abandoned because of the poor performance of DFT overall to describe **1**.
‡ The sextet state was not expected over the doublet or quartet, and we encountered difficulties in optimizing the structures for some functionals. For this reason, we abandoned applying pure functionals to the sextet state.
¶ Benchmark values using domain-based local pair natural orbital coupled cluster with singles, doubles, and perturbative triples using a UHF/def2-TZVPP//PW6B95/def2-SVP reference wavefunction.

Table S 4. Doublet-quartet and doublet-sextet gaps ΔE of complex **1** and the relative error $\sigma(\Delta E)$ in kcal mol^{-1} of each functional compared to the benchmark values.

	**Functional**<br>Type<br>% HF Exchange	Basis set	Doublet-Quartet<br>ΔE / kcal mol^{-1}	 $\sigma(\Delta E)$ / kcal mol^{-1}	Doublet-Sextet<br>ΔE / kcal mol^{-1}	 $\sigma(\Delta E)$ / kcal mol^{-1}
Gaussian 16 Rev. C.01	**APFD**<br>Hybrid GGA<br>23%	def2-SVP	-4.87	23.26	0.00	27.11
		def2-TZVPP	—	—	-6.67	20.44
		def2-QZVPP	-5.12	23.01	-6.76	20.35
	**B97-D3(BJ)**<br>Hybrid GGA<br>19.43%	def2-SVP	-2.84	25.29	-11.75	15.36
		def2-TZVPP	—	—	-12.63	14.48
		def2-QZVPP	-3.32	24.81	—	—
	**PW6B95-D3(BJ)**<br>Hybrid Meta GGA<br>28%	def2-SVP	-3.58	24.55	2.22	29.34
		def2-TZVPP	—	—	0.59	27.70
		def2-QZVPP	-4.00	24.13	0.51	27.62
	**ωB97X-D2**<br>Range-separated Hybrid GGA<br>22% Short, 100% Long	def2-SVP	-3.26	24.87	0.47	27.58
		def2-TZVPP	—	—	-22.40	4.71
		def2-QZVPP	-3.72	24.41	-2.86	24.25
ORCA v4.2.1	**BLYP-D3(BJ)**<br>Pure GGA	def2-SVP	-7.07	21.06	—	—
		def2-QZVPP	-7.57	20.56	—	—
	**PBE-D3(BJ)**<br>Pure GGA	def2-SVP	-6.35	21.78	—	—
		def2-QZVPP	-6.93	21.20	—	—
	**OLYP-D3(BJ)**<br>Pure GGA	def2-SVP	-4.72	23.41	—	—
		def2-QZVPP	-5.21	22.92	—	—
	**OPBE-D3(BJ)**<br>Pure GGA	def2-SVP	-4.89	23.24	—	—
		def2-QZVPP	-5.52	22.61	—	—
	**DLPNO-CCSD(T)¶**<br>Post-HF	def2-TZVPP	-28.13	—	-27.11	—

* Basis set convergence issues were noticed for other calculations with triple-ζ valence quality basis sets, so energies were only calculated at quadruple-ζ quality for S = 3/2 spin states.
† The SCF step failed to converge even after thousands of cycles and was abandoned because of the poor performance of DFT overall to describe **1**.
‡ The sextet state was not expected over the doublet or quartet, and we encountered difficulties in optimizing the structures for some functionals. For this reason, we abandoned applying pure functionals to the sextet state.
¶ Benchmark values using domain-based local pair natural orbital coupled cluster with singles, doubles, and perturbative triples using a UHF/def2-TZVPP//PW6B95/def2-SVP reference wavefunction.

Table S 5: Mulliken population analysis of **1** in the doublet *S* = 1/2 state (def2-QZVPP//OPBE-D3(BJ)/def2-SVP). Total charge = 0.0000000, total spin = 1.0000000.

Index	Element	Charge	Spin	Index	Element	Charge	Spin
0	Co	-0.540484	0.515118	38	H	0.008758	-0.000374
1	Si	0.529322	-0.01558	39	H	0.026565	-0.001144
2	Si	0.529528	-0.015553	40	H	0.039357	-0.001925
3	N	-0.034688	0.130765	41	H	0.062091	0.000731
4	N	-0.034633	0.130693	42	H	0.041171	0.000815
5	N	-0.034603	0.130778	43	H	0.070021	0.000534
6	N	-0.034534	0.130743	44	H	0.045855	-0.000002
7	C	-0.357326	0.007676	45	H	0.050845	-0.0013
8	C	-0.357498	0.007667	46	H	0.062058	0.000718
9	C	-0.449562	-0.001106	47	H	0.026554	-0.001144
10	C	-0.273838	0.003881	48	H	0.050826	-0.001311
11	C	0.753193	-0.013753	49	H	0.050813	-0.001302
12	C	-0.310085	0.004578	50	H	0.026543	-0.001152
13	C	-0.273828	0.00387	51	H	0.045006	-0.000094
14	C	0.753428	-0.01379	52	H	0.056524	-0.000949
15	C	-0.310062	0.00457	53	H	0.045019	-0.00009
16	C	-0.449654	-0.001137	54	H	0.05652	-0.000953
17	C	-0.310021	0.004569	55	H	0.062022	0.000718
18	C	-0.449734	-0.001109	56	H	0.041167	0.000798
19	C	0.753336	-0.013754	57	H	0.045857	-0.000002
20	C	-0.273778	0.003872	58	H	0.062098	0.00073
21	C	-0.449614	-0.001138	59	H	0.045847	-0.000001
22	C	-0.357497	0.007665	60	H	0.070018	0.000531
23	C	-0.273893	0.003832	61	H	0.00877	-0.000373
24	C	0.753371	-0.013736	62	H	0.039354	-0.001924
25	C	-0.309974	0.004571	63	H	0.05084	-0.001306
26	C	-0.357554	0.007691	64	H	0.070056	0.00053
27	H	0.041696	0.001944	65	H	0.05652	-0.00095
28	H	0.03933	-0.001928	66	H	0.054798	-0.001191
29	H	0.054789	-0.001187	67	H	0.041736	0.001956
30	H	0.041724	0.001945	68	H	0.045003	-0.000093
31	H	0.045017	-0.000093	69	H	0.026555	-0.001148
32	H	0.008764	-0.000374	70	H	0.00881	-0.000368
33	H	0.056519	-0.000949	71	H	0.041146	0.000806
34	H	0.054799	-0.001189	72	H	0.054786	-0.001189
35	H	0.070037	0.000535	73	H	0.039346	-0.001924
36	H	0.041153	0.000806	74	H	0.041744	0.001951
37	H	0.045856	-0.000002				

Table S 6: Mulliken population analysis of **1** in the quartet *S* = 3/2 state (def2-QZVPP//OPBE-D3(BJ)/def2-SVP). Total charge = 0.0000000, total spin = 3.0000000.

Index	Element	Charge	Spin	Index	Element	Charge	Spin
0	Co	-0.267865	1.794899	38	H	0.015989	-0.000836
1	Si	0.491994	-0.017969	39	H	0.024563	0.000264
2	Si	0.492515	-0.017874	40	H	0.03694	-0.003913
3	N	-0.049325	0.301587	41	H	0.062233	-0.001317
4	N	-0.049207	0.302047	42	H	0.042995	-0.000191
5	N	-0.049028	0.301411	43	H	0.07695	-0.000384
6	N	-0.049047	0.301887	44	H	0.050699	0.001908
7	C	-0.335947	0.018978	45	H	0.070119	-0.000168
8	C	-0.336015	0.018995	46	H	0.062208	-0.001322
9	C	-0.448435	0.00914	47	H	0.024557	0.000281
10	C	-0.316047	0.01147	48	H	0.070106	-0.000157
11	C	0.719499	-0.025242	49	H	0.07012	-0.000155
12	C	-0.317726	0.001068	50	H	0.024561	0.000269
13	C	-0.31612	0.011543	51	H	0.039858	-0.001815
14	C	0.720386	-0.025108	52	H	0.053786	-0.003176
15	C	-0.317607	0.001052	53	H	0.039854	-0.001818
16	C	-0.448504	0.009139	54	H	0.053864	-0.003175
17	C	-0.31756	0.001071	55	H	0.062255	-0.00131
18	C	-0.448569	0.009119	56	H	0.042973	-0.000186
19	C	0.720378	-0.025086	57	H	0.050692	0.001914
20	C	-0.316139	0.01156	58	H	0.062203	-0.001312
21	C	-0.448469	0.00914	59	H	0.050667	0.001899
22	C	-0.335976	0.018966	60	H	0.076931	-0.000359
23	C	-0.316104	0.011422	61	H	0.015959	-0.000817
24	C	0.720437	-0.024939	62	H	0.036916	-0.003915
25	C	-0.317751	0.001054	63	H	0.070144	-0.000152
26	C	-0.336023	0.019007	64	H	0.076891	-0.000363
27	H	0.034907	0.004709	65	H	0.053825	-0.00318
28	H	0.036919	-0.00392	66	H	0.059108	-0.002229
29	H	0.059058	-0.002239	67	H	0.034906	0.00473
30	H	0.034907	0.004723	68	H	0.039871	-0.001821
31	H	0.03989	-0.001816	69	H	0.024583	0.000282
32	H	0.015939	-0.000808	70	H	0.015919	-0.000792
33	H	0.053837	-0.00318	71	H	0.042972	-0.000182
34	H	0.059036	-0.002231	72	H	0.059116	-0.002223
35	H	0.076871	-0.000385	73	H	0.036922	-0.00391
36	H	0.043038	-0.000202	74	H	0.034898	0.004751
37	H	0.050696	0.001897				

Table S 7: Cartesian geometry of **1** in the doublet *S* = 1/2 state (APFD/def2-SVP).

Element	*x*	*y*	*z*	Element	*x*	*y*	*z*
Co	0.000000	0.000005	0.000000	H	-0.341351	2.818375	2.981771
Si	2.643858	-0.000001	-0.000353	H	3.157238	1.456738	2.779206
Si	-2.643858	0.000001	0.000356	H	-2.781437	3.154308	-0.912220
N	1.373754	0.849005	0.822256	H	0.598217	1.080617	-2.638964
N	-1.373754	0.849032	-0.822227	H	-1.996888	2.311129	-3.822242
N	1.373523	-0.848995	-0.822612	H	-0.840473	2.267370	1.353964
N	-1.373523	-0.849025	0.822581	H	4.419839	-0.441461	1.693714
C	1.730946	3.224981	1.234070	H	1.777694	0.575080	3.477012
C	-1.730932	3.225026	-1.233947	H	-0.598234	1.080537	2.638981
C	3.742916	1.064170	-1.087666	H	-3.157248	1.456850	-2.779141
C	0.221570	2.025737	-2.227233	H	1.776712	-0.575122	-3.477474
C	1.242852	1.886570	1.811964	H	-1.777715	0.575210	-3.476992
C	2.091197	1.536577	3.044445	H	3.156415	-1.456890	-2.780121
C	-0.221573	2.025670	2.227293	H	4.372601	-1.727690	0.471394
C	-1.242853	1.886633	-1.811896	H	-3.142892	1.686128	1.766672
C	-2.091209	1.536693	-3.044385	H	-4.372473	1.727676	0.472608
C	3.743191	-1.064175	1.086677	H	3.143340	-1.686195	1.765767
C	2.090282	-1.536646	-3.045011	H	-0.598972	-1.080446	-2.638726
C	-3.742912	1.064136	1.087708	H	1.995648	-2.311042	-3.822870
C	1.242309	-1.886574	-1.812251	H	-4.419411	0.441402	1.694889
C	-0.222260	-2.025584	-2.227095	H	0.598993	-1.080564	2.638660
C	-3.743195	-1.064135	-1.086707	H	-4.419844	-0.441401	-1.693722
C	1.730493	-3.224998	-1.234459	H	-0.840905	-2.267169	-1.353550
C	0.222263	-2.025684	2.227004	H	-0.342346	-2.818328	-2.981481
C	-1.242308	-1.886643	1.812181	H	2.781098	-3.154334	-0.913045
C	-2.090266	-1.536751	3.044961	H	-1.776683	-0.575245	3.477455
C	-1.730512	-3.225041	1.234346	H	0.840895	-2.267250	1.353444
H	1.658111	4.031815	1.981025	H	-3.143346	-1.686133	-1.765820
H	2.781450	3.154264	0.912337	H	1.129571	-3.512978	-0.359897
H	1.129778	3.513031	0.359701	H	1.657382	-4.031853	-1.981363
H	-1.658086	4.031890	-1.980870	H	-4.372603	-1.727671	-0.471446
H	4.372476	1.727689	-0.472542	H	-3.156401	-1.456978	2.780085
H	0.341349	2.818473	-2.981678	H	0.342347	-2.818454	2.981363
H	3.142898	1.686186	-1.766611	H	-1.995630	-2.311177	3.822792
H	-1.129762	3.513033	-0.359566	H	-1.129593	-3.513003	0.359776
H	0.840480	2.267393	-1.353899	H	-2.781115	-3.154353	0.912935
H	1.996875	2.310984	3.822331	H	-1.657412	-4.031921	1.981225
H	4.419415	0.441455	-1.694866				

Table S 8: Cartesian geometry of 1 in the doublet *S* = 1/2 state (B97-D3(BJ)/def2-SVP).

Element	*x*	*y*	*z*	Element	*x*	*y*	*z*
Co	0.000000	0.000020	0.000010	H	0.137156	-2.884960	3.130655
Si	-2.670189	0.000027	-0.000364	H	-3.331973	-1.492347	2.678782
Si	2.670190	-0.000016	0.000366	H	2.831597	-3.174542	-0.826769
N	-1.402879	-0.859314	0.845189	H	-0.451008	-1.143400	-2.832285
N	1.402860	-0.859385	-0.845130	H	2.234569	-2.331303	-3.810306
N	-1.402637	0.859361	-0.845566	H	0.780727	-2.328817	1.551519
N	1.402655	0.859378	0.845534	H	-4.465029	0.469496	1.693912
C	-1.801231	-3.254424	1.213719	H	-2.002594	-0.588010	3.459396
C	1.801176	-3.254503	-1.213623	H	0.450970	-1.143322	2.832359
C	-3.784833	-1.085488	-1.077857	H	3.331932	-1.492478	-2.678737
C	-0.094199	-2.080203	-2.375647	H	-2.001482	0.587924	-3.459974
C	-1.343320	-1.915692	1.843356	H	2.002560	-0.588136	-3.459356
C	-2.283632	-1.560242	3.019781	H	-3.331135	1.492283	-2.679852
C	0.094152	-2.080129	2.375736	H	-4.414961	1.740729	0.445807
C	1.343280	-1.915776	-1.843284	H	3.186278	-1.722319	1.751234
C	2.283586	-1.560363	-3.019723	H	4.414827	-1.740709	0.447057
C	-3.785078	1.085574	1.076845	H	-3.186706	1.722438	1.750260
C	-2.282678	1.560173	-3.020496	H	0.451877	1.143313	-2.832130
C	3.784808	-1.085507	1.077910	H	-2.233420	2.331063	-3.811113
C	-1.342769	1.915687	-1.843770	H	4.464629	-0.469394	1.695086
C	0.094879	2.080133	-2.375675	H	-0.451838	1.143447	2.832096
C	3.785104	1.085461	-1.076886	H	4.465075	0.469345	-1.693893
C	-1.800913	3.254437	-1.214340	H	0.781171	2.328880	-1.551240
C	-0.094831	2.080242	2.375597	H	0.138114	2.884928	-3.130619
C	1.342810	1.915754	1.843687	H	-2.831461	3.174469	-0.827824
C	2.282726	1.560280	3.020420	H	2.001520	0.588055	3.459944
C	1.800965	3.254469	1.214192	H	-0.781126	2.328961	1.551155
H	-1.775340	-4.071909	1.957743	H	3.186753	1.722285	-1.750358
H	-2.831646	-3.174456	0.826851	H	-1.141463	3.527950	-0.373820
H	-1.141488	-3.527900	0.373417	H	-1.774777	4.071895	-1.958385
H	1.775263	-4.072003	-1.957630	H	4.414967	1.740653	-0.445867
H	-4.414917	-1.740597	-0.446972	H	3.331179	1.492361	2.679769
H	-0.137221	-2.885049	-3.130549	H	-0.138051	2.885072	3.130504
H	-3.186322	-1.722393	-1.751109	H	2.233487	2.331206	3.811004
H	1.141438	-3.527949	-0.373308	H	1.141507	3.527955	0.373669
H	-0.780770	-2.328864	-1.551419	H	2.831506	3.174471	0.827667
H	-2.234635	-2.331167	3.810380	H	1.774848	4.071960	1.958202
H	-4.464592	-0.469381	-1.695107				

Table S 9: Cartesian geometry of **1** in the doublet *S* = 1/2 state (PW6B95-D3(BJ)/def2-SVP).

Element	*x*	*y*	*z*	Element	*x*	*y*	*z*
Co	0.000038	0.000046	0.000081	H	0.167325	-2.807191	3.128238
Si	-2.663447	0.000117	-0.000010	H	-3.293564	-1.462316	2.664444
Si	2.663471	-0.000179	-0.000150	H	2.784459	-3.147732	-0.839305
N	-1.390976	-0.844787	0.835118	H	-0.458896	-1.079796	-2.793135
N	1.390788	-0.845212	-0.834841	H	2.200450	-2.278719	-3.798112
N	-1.390878	0.845008	-0.834997	H	0.787216	-2.280374	1.541928
N	1.391176	0.845035	0.834901	H	-4.453423	0.472339	1.669231
C	-1.765976	-3.220389	1.238610	H	-1.978245	-0.549769	3.431081
C	1.765433	-3.220955	-1.237677	H	0.458392	-1.078968	2.793713
C	-3.758620	-1.077775	-1.073112	H	3.293087	-1.463568	-2.664215
C	-0.109363	-2.024688	-2.362419	H	-1.978374	0.550209	-3.430989
C	-1.320574	-1.883449	1.842399	H	1.977828	-0.551040	-3.430975
C	-2.251738	-1.521931	3.004416	H	-3.293471	1.462975	-2.664238
C	0.108839	-2.023952	2.363199	H	-4.363678	1.754335	0.453961
C	1.320121	-1.884149	-1.841825	H	3.155753	-1.684517	1.758565
C	2.251213	-1.523117	-3.004050	H	4.363792	-1.754350	0.453991
C	-3.758622	1.078075	1.073019	H	-3.155612	1.684694	1.758512
C	-2.251650	1.522403	-3.004258	H	0.458425	1.079055	-2.793809
C	3.758722	-1.078005	1.072941	H	-2.200995	2.277803	-3.798519
C	-1.320359	1.883679	-1.842267	H	4.453518	-0.472143	1.669040
C	0.109053	2.024022	-2.363119	H	-0.458209	1.079741	2.793406
C	3.758586	1.077347	-1.073686	H	4.453340	0.471456	-1.669790
C	-1.765490	3.220656	-1.238363	H	0.787509	2.280193	-1.541833
C	-0.108644	2.024606	2.362649	H	0.167637	2.807378	-3.128029
C	1.320760	1.883961	1.841857	H	-2.784410	3.147552	-0.839696
C	2.251959	1.522863	3.003986	H	1.978444	0.550868	3.431016
C	1.766107	3.220727	1.237634	H	-0.787043	2.280852	1.541345
H	-1.749615	-4.019889	1.990538	H	3.155513	1.683784	-1.759287
H	-2.784970	-3.147181	0.840154	H	-1.100465	3.507928	-0.416325
H	-1.101164	-3.507748	0.416436	H	-1.749194	4.020165	-1.990282
H	1.748940	-4.020668	-1.989375	H	4.363678	1.753803	-0.454870
H	-4.363743	-1.754017	-0.454099	H	3.293765	1.463072	2.663977
H	-0.167993	-2.808128	-3.127240	H	-0.167080	2.808036	3.127498
H	-3.155602	-1.684412	-1.758582	H	2.201475	2.278527	3.798006
H	1.100639	-3.508006	-0.415382	H	1.101210	3.507816	0.415425
H	-0.787656	-2.280844	-1.540995	H	2.785065	3.147378	0.839098
H	-2.201189	-2.277302	3.798712	H	1.749814	4.020467	1.989306
H	-4.453363	-0.471998	-1.669351				

Table S 10: Cartesian geometry of **1** in the doublet *S* = 1/2 state (wB97X-D2/def2-SVP).

Element	*x*	*y*	*z*	Element	*x*	*y*	*z*
Co	0.000000	-0.000001	0.000000	H	-0.181153	2.834664	3.131300
Si	2.635120	-0.000005	-0.000370	H	3.280476	1.485348	2.662026
Si	-2.635120	-0.000005	0.000370	H	-2.763166	3.164364	-0.817811
N	1.370151	0.844752	0.836812	H	0.485386	1.102211	-2.809085
N	-1.370151	0.844781	-0.836782	H	-2.186973	2.319417	-3.789770
N	1.369915	-0.844752	-0.837205	H	-0.823244	2.305230	1.551377
N	-1.369916	-0.844782	0.837174	H	4.423494	-0.474364	1.678891
C	1.737389	3.233793	1.210273	H	1.960613	0.581612	3.445344
C	-1.737392	3.233836	-1.210155	H	-0.485382	1.102106	2.809132
C	3.738986	1.082542	-1.067010	H	-3.280473	1.485441	-2.661976
C	0.134002	2.044849	-2.366555	H	1.959597	-0.581589	-3.445912
C	1.296923	1.898190	1.834675	H	-1.960608	0.581737	-3.445325
C	2.235194	1.550693	3.002781	H	3.279703	-1.485319	-2.662999
C	-0.134001	2.044761	2.366633	H	4.360865	-1.741160	0.437297
C	-1.296923	1.898257	-1.834606	H	-3.140263	1.712666	1.740794
C	-2.235191	1.550802	-3.002727	H	-4.360749	1.741131	0.438552
C	3.739264	-1.082557	1.065977	H	3.140714	-1.712744	1.739853
C	2.234319	-1.550671	-3.003439	H	-0.486205	-1.102116	-2.808973
C	-3.738986	1.082504	1.067048	H	2.185870	-2.319252	-3.790501
C	1.296401	-1.898184	-1.835053	H	-4.423060	0.474288	1.680113
C	-0.134680	-2.044769	-2.366584	H	0.486210	-1.102220	2.808926
C	-3.739265	-1.082520	-1.066015	H	-4.423494	-0.474305	-1.678908
C	1.737069	-3.233786	-1.210792	H	-0.823675	-2.305250	-1.551123
C	0.134682	-2.044857	2.366505	H	-0.182052	-2.834669	-3.131241
C	-1.296401	-1.898251	1.834983	H	2.762958	-3.164320	-0.818746
C	-2.234316	-1.550779	3.003384	H	-1.959591	-0.581714	3.445893
C	-1.737072	-3.233830	1.210674	H	0.823673	-2.305312	1.551034
H	1.709536	4.047939	1.951731	H	-3.140715	-1.712685	-1.739912
H	2.763162	3.164336	0.817922	H	1.073759	-3.504344	-0.376449
H	1.073827	3.504336	0.376126	H	1.709005	-4.047927	-1.952247
H	-1.709537	4.048010	-1.951582	H	-4.360866	-1.741144	-0.437357
H	4.360748	1.741148	-0.438491	H	-3.279700	-1.485413	2.662949
H	0.181154	2.834779	-3.131195	H	0.182053	-2.834784	3.131135
H	3.140262	1.712726	-1.740735	H	-2.185866	-2.319390	3.790418
H	-1.073834	3.504348	-0.375995	H	-1.073765	-3.504356	0.376318
H	0.823242	2.305292	-1.551289	H	-2.762962	-3.164349	0.818635
H	2.186976	2.319279	3.789854	H	-1.709005	-4.047998	1.952099
H	4.423060	0.474347	-1.680095				

Table S 11: Cartesian geometry of **1** in the doublet *S* = 1/2 state (BLYP-D3(BJ)/def2-SVP).

Element	*x*	*y*	*z*	Element	*x*	*y*	*z*
Co	0.000001	0.000007	-0.000002	H	-0.149283	2.891212	3.141814
Si	2.675178	0.000005	-0.000372	H	3.334228	1.486621	2.700392
Si	-2.675176	0.000005	0.000372	H	-2.838888	3.185755	-0.83298
N	1.405935	0.862582	0.848412	H	0.464895	1.146092	-2.834364
N	-1.405934	0.862608	-0.848388	H	-2.234502	2.336229	-3.830863
N	1.405699	-0.862566	-0.84881	H	-0.788925	2.341171	1.553122
N	-1.405696	-0.86259	0.848784	H	4.469478	-0.4756	1.703614
C	1.805324	3.26708	1.221908	H	1.991777	0.589053	3.478579
C	-1.805323	3.267118	-1.221811	H	-0.464896	1.146008	2.834396
C	3.784579	1.090345	-1.084843	H	-3.334229	1.486701	-2.700347
C	0.103215	2.087066	-2.38113	H	1.99072	-0.589005	-3.479161
C	1.343335	1.923165	1.853221	H	-1.991778	0.589159	-3.478563
C	2.281541	1.562457	3.037838	H	3.333437	-1.486532	-2.701389
C	-0.103215	2.086994	2.381189	H	4.412425	-1.756174	0.455321
C	-1.343335	1.923221	-1.853164	H	-3.175334	1.72118	1.760166
C	-2.281542	1.562549	-3.037792	H	-4.412303	1.756166	0.456581
C	3.784864	-1.090341	1.083804	H	3.175802	-1.721229	1.759234
C	2.280649	-1.562401	-3.038512	H	-0.465744	-1.146016	-2.8342
C	-3.784575	1.090314	1.084878	H	2.233388	-2.336056	-3.831595
C	1.342817	-1.923141	-1.853608	H	-4.469025	0.475554	1.704849
C	-0.103892	-2.087003	-2.381131	H	0.465749	-1.146106	2.834174
C	-3.784863	-1.090307	-1.083835	H	-4.469478	-0.475548	-1.703626
C	1.805032	-3.267051	-1.222448	H	-0.789335	-2.341223	-1.552856
C	0.10389	-2.087075	2.381072	H	-0.150172	-2.891203	-3.141762
C	-1.342818	-1.923196	1.853552	H	2.838717	-3.18568	-0.833937
C	-2.280651	-1.562489	3.038465	H	-1.990723	-0.589104	3.47914
C	-1.805032	-3.267085	1.222352	H	0.789331	-2.341284	1.552792
H	1.780551	4.088048	1.96752	H	-3.175802	-1.721174	-1.759285
H	2.83889	3.185728	0.833074	H	1.14392	-3.542343	-0.378709
H	1.143943	3.542352	0.378374	H	1.780043	-4.088014	-1.968058
H	-1.780549	4.088108	-1.967398	H	-4.412424	-1.756158	-0.455371
H	4.412304	1.75618	-0.456525	H	-3.333439	-1.486611	2.701343
H	0.149283	2.891305	-3.141732	H	0.150163	-2.891304	3.141672
H	3.17534	1.721231	-1.760115	H	-2.23339	-2.336164	3.831526
H	-1.143941	3.542364	-0.378269	H	-1.143919	-3.542354	0.378606
H	0.788926	2.341218	-1.553055	H	-2.838716	-3.185701	0.833844
H	2.234499	2.336113	3.830933	H	-1.780044	-4.08807	1.967939
H	4.469033	0.475604	-1.70483				

Table S 12: Cartesian geometry of **1** in the doublet S = 1/2 state (PBE-D3(BJ)/def2-SVP).

Element	*x*	*y*	*z*	Element	*x*	*y*	*z*
Co	0.000001	0.000006	-0.000002	H	-0.147734	2.889784	3.129825
Si	2.669413	0.000003	-0.000369	H	3.324966	1.478778	2.686833
Si	-2.669411	0.000004	0.000369	H	-2.832445	3.172073	-0.830575
N	1.396751	0.860935	0.845058	H	0.460633	1.140035	-2.833238
N	-1.396751	0.86096	-0.845034	H	-2.225904	2.329736	-3.817874
N	1.396517	-0.860919	-0.845452	H	-0.787905	2.326662	1.542213
N	-1.396514	-0.860944	0.845427	H	4.467523	-0.470427	1.694367
C	1.799158	3.253743	1.22295	H	1.981418	0.581958	3.467397
C	-1.799156	3.25378	-1.222853	H	-0.460634	1.139951	2.833268
C	3.779471	1.086429	-1.079923	H	-3.324968	1.478859	-2.686787
C	0.100269	2.080564	-2.373832	H	1.980353	-0.581904	-3.467977
C	1.33747	1.915584	1.846924	H	-1.98142	0.582064	-3.467382
C	2.271359	1.555493	3.024543	H	3.324176	-1.478678	-2.687833
C	-0.10027	2.080493	2.373891	H	4.405587	-1.751725	0.447698
C	-1.33747	1.91564	-1.846868	H	-3.173992	1.717616	1.759293
C	-2.271361	1.555585	-3.024497	H	-4.40547	1.751713	0.448948
C	3.779749	-1.08643	1.078891	H	3.174454	-1.71767	1.758367
C	2.270467	-1.55543	-3.025217	H	-0.46149	-1.139962	-2.833062
C	-3.779466	1.086399	1.079957	H	2.22479	-2.329555	-3.818608
C	1.336955	-1.91556	-1.84731	H	-4.467079	0.470376	1.695592
C	-0.100945	-2.080506	-2.373829	H	0.461493	-1.140052	2.833037
C	-3.77975	-1.086396	-1.078923	H	-4.467525	-0.470375	-1.69438
C	1.798874	-3.253712	-1.223492	H	-0.788308	-2.326726	-1.541942
C	0.100944	-2.080578	2.373771	H	-0.148617	-2.889775	-3.129773
C	-1.336955	-1.915614	1.847254	H	2.832286	-3.171995	-0.831541
C	-2.270468	-1.555516	3.025169	H	-1.980356	-0.582002	3.467956
C	-1.798872	-3.253746	1.223397	H	0.788306	-2.326783	1.541878
H	1.778777	4.07202	1.972293	H	-3.174455	-1.717615	-1.758418
H	2.832447	3.172047	0.830672	H	1.1361	-3.536216	-0.38183
H	1.136111	3.536223	0.381496	H	1.778276	-4.071983	-1.972836
H	-1.778777	4.07208	-1.972171	H	-4.405587	-1.751709	-0.447748
H	4.405471	1.751727	-0.448892	H	-3.324177	-1.478755	2.687788
H	0.147733	2.889878	-3.129742	H	0.148611	-2.889877	3.129683
H	3.173998	1.717666	-1.759243	H	-2.224791	-2.329663	3.818539
H	-1.136108	3.536234	-0.381391	H	-1.136098	-3.536228	0.381729
H	0.787906	2.326707	-1.542147	H	-2.832283	-3.172016	0.831448
H	2.2259	2.329619	3.817945	H	-1.778276	-4.072039	1.972717
H	4.467087	0.470425	-1.695574				

Table S 13: Cartesian geometry of **1** in the doublet *S* = 1/2 state (OLYP-D3(BJ)/def2-SVP).

Element	*x*	*y*	*z*	Element	*x*	*y*	*z*
Co	0.000001	0.00001	-0.000002	H	-0.151704	2.85979	3.092174
Si	2.67629	0.000008	-0.000378	H	3.314936	1.488034	2.654781
Si	-2.676288	0.000007	0.000376	H	-2.814071	3.14734	-0.817298
N	1.403605	0.851899	0.836229	H	0.435767	1.112436	-2.809481
N	-1.403603	0.851923	-0.836206	H	-2.213298	2.316592	-3.786641
N	1.40337	-0.851877	-0.836633	H	-0.775162	2.271954	1.517505
N	-1.403366	-0.851903	0.836605	H	4.455124	-0.46171	1.688524
C	1.788311	3.231334	1.205552	H	1.993615	0.575283	3.42927
C	-1.788308	3.231371	-1.205453	H	-0.435769	1.112351	2.809509
C	3.777498	1.07848	-1.075987	H	-3.314938	1.488117	-2.654731
C	0.095695	2.04895	-2.349412	H	1.992611	-0.575254	-3.429849
C	1.337485	1.899259	1.829104	H	-1.99362	0.575391	-3.429255
C	2.270991	1.548324	2.998444	H	3.314172	-1.487984	-2.655744
C	-0.095696	2.048879	2.349469	H	4.401702	-1.736948	0.44705
C	-1.337485	1.899315	-1.829048	H	-3.169922	1.704704	1.745915
C	-2.270995	1.548418	-2.998396	H	-4.401588	1.736936	0.448328
C	3.777788	-1.078467	1.07493	H	3.170398	-1.704739	1.744979
C	2.270128	-1.54829	-2.999103	H	-0.436589	-1.112343	-2.80936
C	-3.777495	1.078445	1.076019	H	2.212209	-2.31644	-3.787355
C	1.336968	-1.899236	-1.82949	H	-4.454656	0.461667	1.689785
C	-0.096364	-2.048871	-2.349433	H	0.436598	-1.112435	2.809334
C	-3.777785	-1.078436	-1.074965	H	-4.45512	-0.46166	-1.688543
C	1.787989	-3.231309	-1.206073	H	-0.77558	-2.271966	-1.517269
C	0.096364	-2.048942	2.349373	H	-0.152581	-2.859775	-3.09213
C	-1.336968	-1.899291	1.829435	H	2.813867	-3.147283	-0.818221
C	-2.270125	-1.548372	2.999059	H	-1.992605	-0.575348	3.42983
C	-1.787995	-3.231342	1.205981	H	0.775576	-2.272028	1.517203
H	1.762897	4.041986	1.952317	H	-3.170395	-1.704689	-1.745031
H	2.814076	3.147315	0.817399	H	1.126174	-3.501298	-0.371495
H	1.126249	3.501313	0.371167	H	1.762364	-4.041959	-1.952833
H	-1.762897	4.042046	-1.952193	H	-4.401701	-1.736933	-0.447104
H	4.401587	1.736955	-0.448275	H	-3.31417	-1.488054	2.655705
H	0.151701	2.859885	-3.092091	H	0.152573	-2.859876	3.092038
H	3.169926	1.704757	-1.745866	H	-2.212205	-2.316543	3.787291
H	-1.126242	3.501325	-0.371063	H	-1.126184	-3.501309	0.371392
H	0.775163	2.271999	-1.517442	H	-2.813874	-3.1473	0.818137
H	2.213291	2.316473	3.786714	H	-1.762369	-4.042014	1.952717
H	4.454663	0.461722	-1.68977				

Table S 14: Cartesian geometry of **1** in the doublet *S* = 1/2 state (OPBE-D3(BJ)/def2-SVP).

Element	*x*	*y*	*z*	Element	*x*	*y*	*z*
Co	-0.000001	0.000025	-0.000001	H	-0.047505	3.109016	2.913765
Si	2.654118	-0.000006	-0.000394	H	3.447142	1.815346	2.296012
Si	-2.654119	0.000087	0.000389	H	-2.752208	3.320296	-0.422607
N	1.393748	1.005195	0.697707	H	0.211728	1.325038	-2.82972
N	-1.393713	1.005262	-0.697686	H	-2.399077	2.636532	-3.480618
N	1.393518	-1.005202	-0.698089	H	-0.743524	2.312499	1.46019
N	-1.393555	-1.005174	0.698055	H	4.477098	-0.275621	1.699064
C	1.76852	3.415998	0.904991	H	2.226762	0.87557	3.19788
C	-1.768399	3.416084	-0.904903	H	-0.211683	1.324971	2.829749
C	3.746166	0.933072	-1.203826	H	-3.447079	1.815529	-2.295967
C	-0.013672	2.232539	-2.250143	H	2.225763	-0.875779	-3.198544
C	1.398689	2.112694	1.617747	H	-2.226733	0.875736	-3.197862
C	2.430343	1.843166	2.716476	H	3.446411	-1.815495	-2.296977
C	0.01375	2.232479	2.250197	H	4.310056	-1.70787	0.656155
C	-1.398615	2.112787	-1.617695	H	-3.131417	1.427146	1.970672
C	-2.43028	1.843325	-2.716431	H	-4.309799	1.707985	0.657512
C	3.746538	-0.933089	1.202694	H	3.132087	-1.427108	1.969701
C	2.429482	-1.843337	-2.717122	H	-0.212588	-1.325126	-2.829604
C	-3.746131	0.93317	1.203848	H	2.398061	-2.636586	-3.481257
C	1.398165	-2.112765	-1.618051	H	-4.476553	0.275712	1.700434
C	0.013025	-2.23259	-2.250053	H	0.212536	-1.325215	2.829565
C	-3.746573	-0.932925	-1.202725	H	-4.477111	-0.275417	-1.699075
C	1.768203	-3.416014	-0.905303	H	-0.743997	-2.312547	-1.459797
C	-0.013106	-2.232654	2.249986	H	-0.048446	-3.109179	-2.913533
C	-1.398241	-2.112764	1.617985	H	2.752163	-3.320176	-0.423324
C	-2.429551	-1.843333	2.717062	H	-2.225801	-0.875797	3.198512
C	-1.76832	-3.41598	0.905199	H	0.743915	-2.312612	1.459728
H	1.809547	4.255083	1.618108	H	-3.132139	-1.426942	-1.969747
H	2.752325	3.320188	0.422692	H	1.031009	-3.655743	-0.129243
H	1.031083	3.655804	0.129188	H	1.808992	-4.25516	-1.618362
H	-1.809396	4.255191	-1.617996	H	-4.310116	-1.707702	-0.656208
H	4.309861	1.707851	-0.657467	H	-3.446478	-1.815445	2.296914
H	0.047614	3.109092	-2.913687	H	0.048335	-3.109265	2.913439
H	3.131471	1.427094	-1.970636	H	-2.398159	-2.636606	3.481173
H	-1.030953	3.655842	-0.129093	H	-1.031132	-3.655714	0.129136
H	0.743605	2.31251	-1.460135	H	-2.752275	-3.320095	0.423218
H	2.399167	2.636352	3.480685	H	-1.809143	-4.255145	1.618234
H	4.476564	0.275602	-1.700431				

Table S 15: Cartesian geometry of **1** in the quartet *S* = 3/2 state (APFD/def2-SVP).

Element	*x*	*y*	*z*	Element	*x*	*y*	*z*
Co	0.000000	0.000035	0.000001	H	-0.230673	2.966583	2.955133
Si	2.612819	0.000012	-0.000202	H	3.301708	1.739083	2.504913
Si	-2.612820	0.000027	0.000197	H	-2.721283	3.343126	-0.624149
N	1.363160	0.991562	0.718304	H	0.411717	1.204651	-2.701120
N	-1.363153	0.991573	-0.718302	H	-2.174965	2.538438	-3.624396
N	1.363018	-0.991542	-0.718455	H	-0.835183	2.321672	1.396898
N	-1.363027	-0.991531	0.718457	H	4.451909	-0.275733	1.684370
C	1.703466	3.402414	1.039955	H	2.017994	0.789956	3.297052
C	-1.703466	3.402425	-1.039966	H	-0.411671	1.204636	2.701165
C	3.709241	0.932876	-1.206166	H	-3.301674	1.739083	-2.504947
C	0.129743	2.153232	-2.219558	H	2.017401	-0.790174	-3.297370
C	1.299329	2.064648	1.677614	H	-2.017941	0.789959	-3.297061
C	2.254238	1.764666	2.844168	H	3.301175	-1.739407	-2.505458
C	-0.129715	2.153215	2.219589	H	4.264191	-1.720631	0.669440
C	-1.299311	2.064658	-1.677612	H	-3.107818	1.412157	1.991928
C	-2.254198	1.764670	-2.844183	H	-4.264092	1.720671	0.670096
C	3.709431	-0.932853	1.205586	H	3.108131	-1.412107	1.991458
C	2.253627	-1.764883	-2.844475	H	-0.412216	-1.204585	-2.700903
C	-3.709242	0.932898	1.206157	H	2.174139	-2.538693	-3.624622
C	1.298941	-2.064677	-1.677685	H	-4.451642	0.275779	1.685064
C	-0.130238	-2.153150	-2.219309	H	0.412176	-1.204568	2.700937
C	-3.709432	-0.932833	-1.205594	H	-4.451905	-0.275709	-1.684382
C	1.703048	-3.402430	-1.039983	H	-0.835526	-2.321461	-1.396426
C	0.130213	-2.153132	2.219333	H	-0.231454	-2.966576	-2.954751
C	-1.298958	-2.064666	1.677688	H	2.720940	-3.343212	-0.624340
C	-2.253661	-1.764877	2.844467	H	-2.017445	-0.790167	3.297365
C	-1.703051	-3.402418	1.039976	H	0.835515	-2.321433	1.396461
H	1.684807	4.218111	1.780374	H	-3.108132	-1.412090	-1.991463
H	2.721280	3.343122	0.624130	H	1.021849	-3.670733	-0.221081
H	1.022162	3.670867	0.221197	H	1.684169	-4.218190	-1.780326
H	-1.684809	4.218116	-1.780391	H	-4.264198	-1.720608	-0.669450
H	4.264097	1.720647	-0.670108	H	-3.301204	-1.739405	2.505435
H	0.230710	2.966595	-2.955105	H	0.231424	-2.966562	2.954772
H	3.107817	1.412138	-1.991935	H	-2.174180	-2.538686	3.624614
H	-1.022171	3.670891	-0.221205	H	-1.021848	-3.670710	0.221075
H	0.835192	2.321701	-1.396852	H	-2.720943	-3.343206	0.624329
H	2.175016	2.538436	3.624381	H	-1.684169	-4.218183	1.780314
H	4.451636	0.275753	-1.685076				

Table S 16: Cartesian geometry of **1** in the quartet *S* = 3/2 state (B97-D3(BJ)/def2-SVP).

Element	*x*	*y*	*z*	Element	*x*	*y*	*z*
Co	0.000000	-0.000030	0.000000	H	-0.008630	-3.134230	3.012810
Si	-2.643620	-0.000020	-0.000370	H	-3.488840	-1.824310	2.343670
Si	2.643620	-0.000030	0.000370	H	2.772400	-3.347760	-0.437290
N	-1.398040	-1.004410	0.736970	H	-0.217880	-1.357570	-2.886940
N	1.398040	-1.004440	-0.736940	H	2.450200	-2.668780	-3.523290
N	-1.397810	1.004380	-0.737310	H	0.743650	-2.408350	1.552650
N	1.397820	1.004400	0.737280	H	-4.483170	0.299980	1.705170
C	-1.789980	-3.442280	0.931490	H	-2.265620	-0.902710	3.269700
C	1.789980	-3.442310	-0.931390	H	0.217880	-1.357480	2.886990
C	-3.754140	-0.957170	-1.198360	H	3.488840	-1.824380	-2.343620
C	0.031360	-2.272560	-2.321950	H	-2.264780	0.902930	-3.270300
C	-1.418300	-2.129220	1.661140	H	2.265620	-0.902810	-3.269670
C	-2.469300	-1.863420	2.766550	H	-3.488120	1.824710	-2.344620
C	-0.031360	-2.272490	2.322020	H	-4.329290	1.722570	0.642040
C	1.418300	-2.129270	-1.661080	H	3.150070	-1.470860	1.965990
C	2.469290	-1.863500	-2.766500	H	4.329100	-1.722610	0.643290
C	-3.754490	0.957100	1.197320	H	-3.150640	1.470830	1.965080
C	-2.468440	1.863660	-2.767180	H	0.218740	1.357410	-2.886790
C	3.754140	-0.957130	1.198390	H	-2.448980	2.668930	-3.523960
C	-1.417750	2.129240	-1.661420	H	4.482670	-0.300010	1.706450
C	-0.030590	2.272420	-2.321850	H	-0.218740	1.357490	2.886750
C	3.754490	0.957060	-1.197350	H	4.483170	0.299920	-1.705180
C	-1.789450	3.442290	-0.931760	H	0.744190	2.408160	-1.552230
C	0.030590	2.272480	2.321790	H	-0.007560	3.134190	-3.012600
C	1.417750	2.129290	1.661360	H	-2.772010	3.347840	-0.437930
C	2.468440	1.863730	2.767120	H	2.264780	0.903020	3.270270
C	1.789450	3.442310	0.931660	H	-0.744180	2.408210	1.552160
H	-1.840390	-4.288640	1.640940	H	3.150640	1.470770	-1.965130
H	-2.772410	-3.347740	0.437380	H	-1.043810	3.685370	-0.158730
H	-1.044180	-3.685540	0.158690	H	-1.839540	4.288720	-1.641150
H	1.840380	-4.288690	-1.640820	H	4.329290	1.722550	-0.642090
H	-4.329110	-1.722630	-0.643240	H	3.488120	1.824780	2.344570
H	0.008630	-3.134320	-3.012720	H	0.007570	3.134280	3.012500
H	-3.150060	-1.470920	-1.965940	H	2.448980	2.669030	3.523890
H	1.044180	-3.685550	-0.158580	H	1.043810	3.685380	0.158620
H	-0.743650	-2.408390	-1.552580	H	2.772020	3.347850	0.437830
H	-2.450210	-2.668670	3.523370	H	1.839550	4.288760	1.641030
H	-4.482670	-0.300060	-1.706440				

Table S 17: Cartesian geometry of **1** in the quartet *S* = 3/2 state (PW6B95-D3(BJ)/def2-SVP).

Element	*x*	*y*	*z*	Element	*x*	*y*	*z*
Co	0.000000	0.000012	0.000016	H	0.010843	3.051398	3.036673
Si	2.638604	-0.000031	-0.000373	H	3.469754	1.799011	2.307466
Si	-2.638605	0.000058	0.000374	H	-2.736185	3.323065	-0.452451
N	1.389111	0.989967	0.726279	H	0.208147	1.290436	-2.861780
N	-1.389071	0.990043	-0.726225	H	-2.444957	2.603961	-3.508949
N	1.388863	-0.990007	-0.726628	H	-0.738347	2.371818	1.567262
N	-1.388905	-0.989973	0.726622	H	4.463011	-0.304209	1.694785
C	1.764999	3.407825	0.954478	H	2.274763	0.853604	3.223012
C	-1.764888	3.407913	-0.954424	H	-0.208060	1.290389	2.861856
C	3.731641	0.948938	-1.193037	H	-3.469664	1.799169	-2.307458
C	-0.035751	2.216694	-2.325473	H	2.273735	-0.853688	-3.223658
C	1.407985	2.095938	1.661319	H	-2.274689	0.853710	-3.222972
C	2.461429	1.820645	2.740738	H	3.468994	-1.799111	-2.308477
C	0.035855	2.216650	2.325560	H	4.294737	-1.719879	0.647236
C	-1.407899	2.096017	-1.661264	H	-3.123232	1.447721	1.957214
C	-2.461328	1.820761	-2.740706	H	-4.294599	1.719845	0.648521
C	3.731954	-0.949047	1.191967	H	3.123802	-1.447718	1.956307
C	2.460530	-1.820727	-2.741429	H	-0.208987	-1.290425	-2.861708
C	-3.731608	0.949033	1.193065	H	2.443948	-2.603942	-3.509653
C	1.407423	-2.095980	-1.661669	H	-4.462476	0.304160	1.696114
C	0.035070	-2.216671	-2.325453	H	0.208909	-1.290542	2.861700
C	-3.731991	-0.948880	-1.191994	H	-4.463016	-0.303989	-1.694791
C	1.764636	-3.407862	-0.954917	H	-0.738891	-2.371783	-1.566895
C	-0.035170	-2.216761	2.325407	H	0.009798	-3.051447	-3.036523
C	-1.407508	-2.095995	1.661605	H	2.736096	-3.323005	-0.453264
C	-2.460624	-1.820763	2.741362	H	-2.273797	-0.853761	3.223652
C	-1.764751	-3.407828	0.954780	H	0.738799	-2.371870	1.566856
H	1.820701	4.238028	1.670150	H	-3.123869	-1.447560	-1.956351
H	2.736290	3.322956	0.452498	H	1.016541	-3.660099	-0.197653
H	1.016656	3.660098	0.197473	H	1.820076	-4.238082	-1.670589
H	-1.820566	4.238118	-1.670096	H	-4.294813	-1.719697	-0.647281
H	4.294670	1.719705	-0.648466	H	-3.469079	-1.799078	2.308392
H	-0.010697	3.051454	-3.036572	H	-0.009938	-3.051565	3.036446
H	3.123290	1.447680	-1.957170	H	-2.444088	-2.604023	3.509540
H	-1.016545	3.660169	-0.197413	H	-1.016664	-3.660038	0.197501
H	0.738439	2.371826	-1.567155	H	-2.736211	-3.322922	0.453134
H	2.445107	2.603848	3.508979	H	-1.820208	-4.238087	1.670407
H	4.462478	0.304045	-1.696106				

Table S 18: Cartesian geometry of **1** in the quartet *S* = 3/2 state (wB97X-D2/def2-SVP).

Element	*x*	*y*	*z*	Element	*x*	*y*	*z*
Co	0.000000	0.000049	0.000000	H	-0.009038	3.118172	2.997399
Si	2.606846	0.000029	-0.000360	H	3.445454	1.812655	2.320445
Si	-2.606844	0.000030	0.000358	H	-2.739041	3.330841	-0.420976
N	1.363048	1.006016	0.708715	H	0.247856	1.352287	-2.852511
N	-1.363047	1.006035	-0.708690	H	-2.408650	2.645321	-3.498894
N	1.362821	-1.005971	-0.709021	H	-0.769708	2.420891	1.536877
N	-1.362822	-1.005988	0.708998	H	4.438949	-0.292525	1.697672
C	1.763044	3.430100	0.919824	H	2.222275	0.888916	3.233099
C	-1.763054	3.430126	-0.919729	H	-0.247839	1.352195	2.852563
C	3.707114	0.944263	-1.196685	H	-3.445446	1.812711	-2.320409
C	-0.006568	2.268633	-2.297578	H	2.221440	-0.889168	-3.233636
C	1.385168	2.124071	1.638688	H	-2.222254	0.889011	-3.233086
C	2.426896	1.849866	2.737746	H	3.444710	-1.813166	-2.321367
C	0.006574	2.268558	2.297652	H	4.275216	-1.715367	0.648836
C	-1.385167	2.124119	-1.638629	H	-3.102410	1.447899	1.965688
C	-2.426886	1.849943	-2.737702	H	-4.275038	1.715415	0.650061
C	3.707458	-0.944189	1.195655	H	3.102977	-1.447859	1.964813
C	2.426007	-1.850152	-2.738326	H	-0.248750	-1.352142	-2.852291
C	-3.707115	0.944233	1.196705	H	2.407339	-2.645520	-3.499518
C	1.384583	-2.124098	-1.638910	H	-4.438457	0.292566	1.698933
C	0.005756	-2.268490	-2.297401	H	0.248741	-1.352221	2.852267
C	-3.707455	-0.944157	-1.195684	H	-4.438942	-0.292479	-1.697688
C	1.762466	-3.430093	-0.919993	H	-0.770284	-2.420693	-1.536347
C	-0.005762	-2.268551	2.297345	H	-0.010176	-3.118155	-2.997078
C	-1.384586	-2.124141	1.638853	H	2.738611	-3.330918	-0.421528
C	-2.426014	-1.850230	2.738274	H	-2.221452	-0.889259	3.233611
C	-1.762464	-3.430116	0.919896	H	0.770282	-2.420730	1.536290
H	1.826661	4.267219	1.632659	H	-3.102973	-1.447810	-1.964852
H	2.739033	3.330837	0.421069	H	1.019450	-3.689727	-0.154804
H	1.019860	3.689954	0.154882	H	1.825746	-4.267313	-1.632739
H	-1.826679	4.267264	-1.632542	H	-4.275217	-1.715347	-0.648886
H	4.275036	1.715433	-0.650022	H	-3.444716	-1.813236	2.321314
H	0.009043	3.118266	-2.997302	H	0.010169	-3.118240	2.996994
H	3.102407	1.447947	-1.965655	H	-2.407344	-2.645618	3.499445
H	-1.019871	3.689965	-0.154781	H	-1.019448	-3.689723	0.154699
H	0.769706	2.420952	-1.536792	H	-2.738610	-3.330931	0.421437
H	2.408659	2.645219	3.498964	H	-1.825739	-4.267359	1.632618
H	4.438458	0.292610	-1.698929				

Table S 19: Cartesian geometry of **1** in the quartet *S* = 3/2 state (BLYP-D3(BJ)/def2-SVP).

Element	*x*	*y*	*z*	Element	*x*	*y*	*z*
Co	0	0.000126	0.000001	H	0.01003	3.154408	3.011911
Si	2.650609	0.000102	-0.00041	H	3.503199	1.839329	2.335473
Si	-2.65061	0.000192	0.000406	H	-2.780528	3.370436	-0.418397
N	1.402007	1.019302	0.720385	H	0.216558	1.372703	-2.883941
N	-1.401973	1.019369	-0.72036	H	-2.465201	2.693253	-3.517166
N	1.401765	-1.019143	-0.720717	H	-0.74859	2.427977	1.548723
N	-1.401801	-1.019117	0.720684	H	4.491753	-0.28565	1.718675
C	1.795981	3.466488	0.916157	H	2.275541	0.921819	3.269467
C	-1.795858	3.466574	-0.916072	H	-0.216509	1.372641	2.883975
C	3.757168	0.946918	-1.217131	H	-3.503134	1.839511	-2.335428
C	-0.031625	2.291274	-2.31706	H	2.274306	-0.922391	-3.270224
C	1.423993	2.148919	1.651778	H	-2.275509	0.921981	-3.269445
C	2.480815	1.883072	2.760154	H	3.502336	-1.839625	-2.33645
C	0.031705	2.291217	2.317116	H	4.329326	-1.728904	0.673646
C	-1.423918	2.149009	-1.651725	H	-3.143836	1.44572	1.992749
C	-2.480748	1.883228	-2.760108	H	-4.329015	1.729266	0.675071
C	3.757584	-0.946696	1.215945	H	3.144579	-1.445452	1.991734
C	2.47978	-1.883489	-2.760702	H	-0.217652	-1.373101	-2.883517
C	-3.757131	0.947015	1.217157	H	2.463952	-2.693724	-3.517531
C	1.423406	-2.149	-1.651816	H	-4.491164	0.285997	1.720119
C	0.030835	-2.291514	-2.316514	H	0.217601	-1.373201	2.883474
C	-3.757622	-0.946531	-1.215972	H	-4.49177	-0.285445	-1.71868
C	1.795673	-3.466338	-0.91593	H	-0.749125	-2.42809	-1.547736
C	-0.030918	-2.291587	2.316442	H	0.008879	-3.154898	-3.01106
C	-1.423483	-2.149002	1.651748	H	2.780547	-3.370033	-0.418689
C	-2.479849	-1.883488	2.760641	H	-2.27434	-0.922414	3.270193
C	-1.795797	-3.466304	0.91582	H	0.749038	-2.428167	1.54766
H	1.848879	4.316827	1.626297	H	-3.144638	-1.445287	-1.991778
H	2.780647	3.370328	0.41848	H	1.046914	-3.709914	-0.141003
H	1.046911	3.710335	0.141626	H	1.848261	-4.316908	-1.625815
H	-1.848726	4.316932	-1.62619	H	-4.329389	-1.728734	-0.673693
H	4.329081	1.729131	-0.675021	H	-3.502403	-1.839574	2.33639
H	-0.009919	3.154481	-3.011834	H	-0.008994	-3.154993	3.010961
H	3.143894	1.445671	-1.99271	H	-2.464051	-2.693747	3.517444
H	-1.046779	3.710374	-0.141534	H	-1.047046	-3.709884	0.140887
H	0.748676	2.427989	-1.548664	H	-2.780667	-3.369948	0.418582
H	2.465296	2.693078	3.517233	H	-1.848416	-4.316894	1.625679
H	4.491176	0.285887	-1.720111				

Table S 20: Cartesian geometry of **1** in the quartet *S* = 3/2 state (PBE-D3(BJ)/def2-SVP).

Element	*x*	*y*	*z*	Element	*x*	*y*	*z*
Co	0	0.00017	0.000003	H	0.009263	3.148045	3.008133
Si	2.642056	0.000153	-0.000422	H	3.490178	1.820928	2.328927
Si	-2.642059	0.000242	0.000416	H	-2.774798	3.355323	-0.428815
N	1.390693	1.012731	0.726552	H	0.217103	1.36432	-2.887008
N	-1.390659	1.012798	-0.726526	H	-2.456215	2.679318	-3.512186
N	1.390445	-1.012501	-0.726858	H	-0.748673	2.414318	1.544571
N	-1.390483	-1.012475	0.726825	H	4.481886	-0.290749	1.712619
C	1.788777	3.449383	0.926085	H	2.258965	0.907576	3.265544
C	-1.788654	3.449468	-0.926	H	-0.217053	1.364259	2.887045
C	3.74831	0.950581	-1.206964	H	-3.490113	1.821108	-2.328884
C	-0.032053	2.281768	-2.316488	H	2.257649	-0.908311	-3.26633
C	1.416064	2.136945	1.653484	H	-2.258931	0.907736	-3.265523
C	2.467277	1.868629	2.754893	H	3.489275	-1.821282	-2.329887
C	0.032134	2.281711	2.316546	H	4.322842	-1.725264	0.655155
C	-1.415988	2.137035	-1.65343	H	-3.137088	1.458417	1.979102
C	-2.46721	1.868783	-2.754847	H	-4.3225	1.725754	0.656638
C	3.74876	-0.95024	1.205732	H	3.137882	-1.458046	1.97805
C	2.466188	-1.869155	-2.75538	H	-0.218283	-1.364873	-2.886464
C	-3.748275	0.950675	1.206988	H	2.454892	-2.679978	-3.512407
C	1.415459	-2.137034	-1.653398	H	-4.481266	0.291222	1.714117
C	0.031221	-2.282102	-2.315747	H	0.218233	-1.364971	2.88642
C	-3.748798	-0.950077	-1.205763	H	-4.481904	-0.290545	-1.712625
C	1.788481	-3.44916	-0.925606	H	-0.749213	-2.414466	-1.543337
C	-0.031304	-2.282174	2.315674	H	0.008056	-3.148694	-3.006999
C	-1.415537	-2.137037	1.65333	H	2.774846	-3.354787	-0.428899
C	-2.466256	-1.869154	2.755321	H	-2.257682	-0.908334	3.2663
C	-1.788606	-3.449127	0.925499	H	0.749125	-2.414542	1.543261
H	1.841407	4.298234	1.638693	H	-3.137943	-1.457881	-1.978099
H	2.774917	3.355214	0.428896	H	1.040395	-3.697063	-0.149095
H	1.04035	3.697639	0.150025	H	1.84078	-4.298321	-1.637869
H	-1.841252	4.298339	-1.638586	H	-4.322904	-1.725097	-0.655205
H	4.322565	1.72562	-0.656588	H	-3.489341	-1.821231	2.329829
H	-0.009151	3.148117	-3.008054	H	-0.008169	-3.148788	3.0069
H	3.137145	1.458372	-1.979064	H	-2.454988	-2.68	3.512323
H	-1.04022	3.697676	-0.149932	H	-1.040529	-3.697034	0.148979
H	0.748758	2.414329	-1.544509	H	-2.774967	-3.354704	0.428795
H	2.456312	2.679145	3.512252	H	-1.840934	-4.298308	1.637735
H	4.481276	0.291115	-1.714112				

Table S 21: Cartesian geometry of **1** in the quartet *S* = 3/2 state (OLYP-D3(BJ)/def2-SVP).

Element	*x*	*y*	*z*	Element	*x*	*y*	*z*
Co	-0.000001	0.000098	-0.000001	H	0.002541	3.128909	2.957483
Si	2.648746	0.000077	-0.000405	H	3.47579	1.824089	2.298601
Si	-2.648746	0.000168	0.000398	H	-2.763745	3.330465	-0.416388
N	1.399732	1.008042	0.711699	H	0.191974	1.34926	-2.864243
N	-1.399698	1.008109	-0.711677	H	-2.43942	2.662274	-3.482135
N	1.399499	-1.007929	-0.71204	H	-0.734193	2.359314	1.512883
N	-1.399536	-1.007901	0.712006	H	4.474429	-0.27596	1.702546
C	1.784644	3.429596	0.908007	H	2.260889	0.898424	3.222076
C	-1.784521	3.429682	-0.907918	H	-0.191931	1.349192	2.864272
C	3.746192	0.936854	-1.206564	H	-3.475728	1.824275	-2.298553
C	-0.036466	2.256792	-2.285646	H	2.259674	-0.898878	-3.222819
C	1.417011	2.123627	1.63123	H	-2.260862	0.898592	-3.222056
C	2.462781	1.859682	2.727157	H	3.474968	-1.82428	-2.299601
C	0.036543	2.256732	2.2857	H	4.312977	-1.712135	0.661731
C	-1.416937	2.12372	-1.631177	H	-3.13351	1.431496	1.975643
C	-2.462718	1.859843	-2.72711	H	-4.31268	1.712442	0.663134
C	3.746602	-0.936682	1.205393	H	3.134239	-1.431274	1.974648
C	2.461789	-1.860006	-2.727743	H	-0.193045	-1.349646	-2.863871
C	-3.746158	0.93695	1.206585	H	2.43824	-2.662605	-3.482582
C	1.416464	-2.123711	-1.631331	H	-4.473847	0.276254	1.703969
C	0.035728	-2.257041	-2.285191	H	0.192991	-1.349734	2.863831
C	-3.746637	-0.936518	-1.205425	H	-4.474443	-0.275758	-1.702556
C	1.784423	-3.429478	-0.907916	H	-0.734678	-2.359501	-1.512017
C	-0.035811	-2.257104	2.285124	H	0.001493	-3.129373	-2.95676
C	-1.41654	-2.123709	1.631264	H	2.76385	-3.330084	-0.416818
C	-2.461859	-1.860002	2.72768	H	-2.259714	-0.898895	3.222785
C	-1.78454	-3.429443	0.90781	H	0.734594	-2.359567	1.511949
H	1.835299	4.268947	1.620703	H	-3.134293	-1.431108	-1.974696
H	2.763866	3.330356	0.416476	H	1.039893	-3.670616	-0.139342
H	1.039789	3.67093	0.139816	H	1.834798	-4.26902	-1.620407
H	-1.835146	4.269055	-1.620591	H	-4.313036	-1.711968	-0.661785
H	4.312742	1.712309	-0.663089	H	-3.475036	-1.82423	2.299537
H	-0.002433	3.128987	-2.957405	H	-0.001606	-3.129457	2.956669
H	3.133563	1.431446	-1.975607	H	-2.43834	-2.662623	3.482496
H	-1.039656	3.670967	-0.139721	H	-1.040014	-3.670585	0.139234
H	0.734276	2.359325	-1.512827	H	-2.76396	-3.330003	0.416711
H	2.439511	2.662093	3.482205	H	-1.834946	-4.269003	1.620278
H	4.473857	0.276145	-1.703967				

Table S 22: Cartesian geometry of **1** in the quartet *S* = 3/2 state (OPBE-D3(BJ)/def2-SVP).

Element	*x*	*y*	*z*	Element	*x*	*y*	*z*
Co	-0.000001	0.000025	-0.000001	H	-0.047505	3.109016	2.913765
Si	2.654118	-0.000006	-0.000394	H	3.447142	1.815346	2.296012
Si	-2.654119	0.000087	0.000389	H	-2.752208	3.320296	-0.422607
N	1.393748	1.005195	0.697707	H	0.211728	1.325038	-2.82972
N	-1.393713	1.005262	-0.697686	H	-2.399077	2.636532	-3.480618
N	1.393518	-1.005202	-0.698089	H	-0.743524	2.312499	1.46019
N	-1.393555	-1.005174	0.698055	H	4.477098	-0.275621	1.699064
C	1.76852	3.415998	0.904991	H	2.226762	0.87557	3.19788
C	-1.768399	3.416084	-0.904903	H	-0.211683	1.324971	2.829749
C	3.746166	0.933072	-1.203826	H	-3.447079	1.815529	-2.295967
C	-0.013672	2.232539	-2.250143	H	2.225763	-0.875779	-3.198544
C	1.398689	2.112694	1.617747	H	-2.226733	0.875736	-3.197862
C	2.430343	1.843166	2.716476	H	3.446411	-1.815495	-2.296977
C	0.01375	2.232479	2.250197	H	4.310056	-1.70787	0.656155
C	-1.398615	2.112787	-1.617695	H	-3.131417	1.427146	1.970672
C	-2.43028	1.843325	-2.716431	H	-4.309799	1.707985	0.657512
C	3.746538	-0.933089	1.202694	H	3.132087	-1.427108	1.969701
C	2.429482	-1.843337	-2.717122	H	-0.212588	-1.325126	-2.829604
C	-3.746131	0.93317	1.203848	H	2.398061	-2.636586	-3.481257
C	1.398165	-2.112765	-1.618051	H	-4.476553	0.275712	1.700434
C	0.013025	-2.23259	-2.250053	H	0.212536	-1.325215	2.829565
C	-3.746573	-0.932925	-1.202725	H	-4.477111	-0.275417	-1.699075
C	1.768203	-3.416014	-0.905303	H	-0.743997	-2.312547	-1.459797
C	-0.013106	-2.232654	2.249986	H	-0.048446	-3.109179	-2.913533
C	-1.398241	-2.112764	1.617985	H	2.752163	-3.320176	-0.423324
C	-2.429551	-1.843333	2.717062	H	-2.225801	-0.875797	3.198512
C	-1.76832	-3.41598	0.905199	H	0.743915	-2.312612	1.459728
H	1.809547	4.255083	1.618108	H	-3.132139	-1.426942	-1.969747
H	2.752325	3.320188	0.422692	H	1.031009	-3.655743	-0.129243
H	1.031083	3.655804	0.129188	H	1.808992	-4.25516	-1.618362
H	-1.809396	4.255191	-1.617996	H	-4.310116	-1.707702	-0.656208
H	4.309861	1.707851	-0.657467	H	-3.446478	-1.815445	2.296914
H	0.047614	3.109092	-2.913687	H	0.048335	-3.109265	2.913439
H	3.131471	1.427094	-1.970636	H	-2.398159	-2.636606	3.481173
H	-1.030953	3.655842	-0.129093	H	-1.031132	-3.655714	0.129136
H	0.743605	2.31251	-1.460135	H	-2.752275	-3.320095	0.423218
H	2.399167	2.636352	3.480685	H	-1.809143	-4.255145	1.618234
H	4.476564	0.275602	-1.700431				

Table S 23: Cartesian geometry of **1** in the sextet *S* = 5/2 state (APFD/def2-SVP).

Element	*x*	*y*	*z*	Element	*x*	*y*	*z*
Co	0.000000	-0.000002	0.000000	H	-0.112254	2.888221	3.093178
Si	2.689988	-0.000006	-0.000373	H	3.404924	1.523346	2.717519
Si	-2.689988	-0.000006	0.000373	H	-2.915735	3.175876	-0.855484
N	1.452786	0.885740	0.873981	H	0.358099	1.136372	-2.822970
N	-1.452786	0.885771	-0.873950	H	-2.284547	2.336007	-3.836946
N	1.452542	-0.885741	-0.874391	H	-0.674756	2.273714	1.506667
N	-1.452542	-0.885772	0.874360	H	4.452254	-0.471319	1.713406
C	1.886041	3.251373	1.236085	H	2.082004	0.600393	3.472977
C	-1.886037	3.251418	-1.235964	H	-0.358102	1.136271	2.823007
C	3.789084	1.079823	-1.078975	H	-3.404926	1.523451	-2.717461
C	0.011375	2.072039	-2.360582	H	2.080949	-0.600358	-3.473578
C	1.430076	1.923439	1.865729	H	-2.082009	0.600522	-3.472955
C	2.354021	1.576156	3.042441	H	3.404122	-1.523292	-2.718543
C	-0.011375	2.071955	2.360655	H	4.432091	-1.728533	0.460710
C	-1.430076	1.923507	-1.865659	H	-3.179414	1.720675	1.733347
C	-2.354024	1.576270	-3.042382	H	-4.431971	1.728500	0.461962
C	3.789361	-1.079840	1.077944	H	3.179858	-1.720749	1.732413
C	2.353120	-1.576120	-3.043136	H	-0.358946	-1.136299	-2.822860
C	-3.789084	1.079787	1.079012	H	2.283411	-2.335821	-3.837714
C	1.429548	-1.923433	-1.866141	H	-4.451816	0.471245	1.714623
C	-0.012054	-2.071977	-2.360620	H	0.358943	-1.136401	2.822822
C	-3.789361	-1.079803	-1.077981	H	-4.452253	-0.471261	-1.713424
C	1.885735	-3.251363	-1.236649	H	-0.675164	-2.273754	-1.506425
C	0.012053	-2.072062	2.360546	H	-0.113142	-2.888242	-3.093115
C	-1.429548	-1.923501	1.866071	H	2.915554	-3.175817	-0.856493
C	-2.353123	-1.576233	3.043078	H	-2.080953	-0.600486	3.473556
C	-1.885733	-3.251407	1.236529	H	0.675165	-2.273806	1.506345
H	1.852920	4.067577	1.975429	H	-3.179857	-1.720691	-1.732470
H	2.915740	3.175842	0.855603	H	1.231537	-3.522709	-0.394722
H	1.231571	3.522702	0.394364	H	1.852394	-4.067563	-1.975987
H	-1.852915	4.067650	-1.975278	H	-4.432092	-1.728516	-0.460768
H	4.431970	1.728516	-0.461903	H	-3.404125	-1.523395	2.718485
H	0.112254	2.888332	-3.093074	H	0.113141	-2.888354	3.093010
H	3.179415	1.720732	-1.733290	H	-2.283413	-2.335964	3.837627
H	-1.231565	3.522715	-0.394235	H	-1.231533	-3.522722	0.394594
H	0.674758	2.273764	-1.506587	H	-2.915551	-3.175848	0.856374
H	2.284545	2.335864	3.837033	H	-1.852393	-4.067635	1.975837
H	4.451818	0.471302	-1.714605				

Table S 24: Cartesian geometry of **1** in the sextet *S* = 5/2 state (B97-D3(BJ)/def2-SVP).

Element	*x*	*y*	*z*	Element	*x*	*y*	*z*
Co	-0.000214	0.000040	0.000234	H	-0.073615	-2.985688	3.202211
Si	-2.679290	-0.000243	-0.000514	H	-3.478001	-1.455389	2.678125
Si	2.670797	-0.000510	-0.000560	H	2.674638	-3.251037	-0.610647
N	-1.449503	-0.966038	0.827162	H	-0.018569	-1.058524	-3.233131
N	1.444016	-0.823306	-0.974021	H	2.677893	-2.496507	-3.692409
N	-1.449295	0.966123	-0.827068	H	0.600446	-2.512665	1.608880
N	1.445036	0.822760	0.973541	H	-4.517194	0.407615	1.685566
C	-2.023843	-3.302964	1.257556	H	-2.118465	-0.565840	3.424891
C	1.726647	-3.261801	-1.173472	H	0.323108	-1.269618	2.847321
C	-3.794832	-1.034598	-1.130753	H	3.637437	-1.707214	-2.409863
C	0.164302	-1.995886	-2.680833	H	-2.121837	0.570299	-3.424668
C	-1.500708	-1.977502	1.868066	H	2.545449	-0.727240	-3.433780
C	-2.433418	-1.544281	3.023191	H	-3.479393	1.460272	-2.674748
C	-0.078240	-2.204154	2.421478	H	-4.382160	1.751512	0.524151
C	1.506147	-1.922329	-1.921175	H	3.176042	-1.852405	1.613267
C	2.660327	-1.700660	-2.925691	H	4.422898	-1.778722	0.327869
C	-3.796554	1.033468	1.128638	H	-3.198761	1.607482	1.857947
C	-2.435164	1.548505	-3.021110	H	0.321305	1.270050	-2.848920
C	3.782569	-1.169610	0.993067	H	-2.412096	2.289616	-3.840562
C	-1.500453	1.978986	-1.866607	H	4.450340	-0.598074	1.663745
C	-0.078357	2.204548	-2.421409	H	-0.016565	1.058974	3.233426
C	3.782277	1.167905	-0.995366	H	4.448983	0.595811	-1.666633
C	-2.021112	3.304319	-1.253672	H	0.601657	2.511151	-1.609194
C	0.166562	1.996099	2.680813	H	-0.073652	2.987088	-3.201131
C	1.508135	1.921665	1.920792	H	-3.051342	3.185311	-0.876627
C	2.662406	1.698989	2.924989	H	2.546936	0.725569	3.432949
C	1.729539	3.261029	1.173175	H	-0.661501	2.133771	1.964393
H	-2.022567	-4.111230	2.012015	H	3.175458	1.850862	-1.615098
H	-3.054466	-3.183165	0.881827	H	-1.380829	3.609462	-0.408216
H	-1.385128	-3.609985	0.411578	H	-2.019874	4.113530	-2.007120
H	1.756225	-4.106565	-1.885912	H	4.423693	1.776828	-0.331045
H	-4.380195	-1.753389	-0.526931	H	3.639381	1.704874	2.408887
H	0.156626	-2.835964	-3.398389	H	0.159531	2.836350	3.398172
H	-3.195896	-1.607886	-1.859701	H	2.680807	2.494699	3.691834
H	0.903818	-3.436613	-0.460314	H	0.906821	3.436495	0.460057
H	-0.663550	-2.133350	-1.964145	H	2.677485	3.249581	0.610281
H	-2.410007	-2.284246	3.843669	H	1.759793	4.105743	1.885647
H	-4.515568	-0.409303	-1.688160				

Table S 25: Cartesian geometry of **1** in the sextet *S* = 5/2 state (PW6B95-D3(BJ)/def2-SVP).

Element	*x*	*y*	*z*	Element	*x*	*y*	*z*
Co	0.000014	-0.017632	0.000065	H	-0.228951	-2.918108	3.237068
Si	-2.704052	0.019410	-0.047125	H	-3.650801	-1.570497	2.438995
Si	2.704004	0.019626	0.047043	H	2.935696	-3.174707	-0.646323
N	-1.500998	-0.890267	0.849775	H	-0.066469	-1.174648	-3.021787
N	1.500980	-0.890196	-0.849774	H	2.663537	-2.344237	-3.693239
N	-1.446308	0.878428	-0.917759	H	0.538556	-2.306264	1.747421
N	1.446247	0.878433	0.917852	H	-4.455480	0.548941	1.657453
C	-1.962991	-3.254932	1.145496	H	-2.448826	-0.613681	3.331848
C	1.963465	-3.254744	-1.145438	H	0.066519	-1.174259	3.021699
C	-3.826379	-1.049270	-1.109612	H	3.650924	-1.569936	-2.438955
C	0.224838	-2.098671	-2.508206	H	-2.168810	0.583307	-3.476251
C	-1.590135	-1.936548	1.838600	H	2.448722	-0.613499	-3.331915
C	-2.650508	-1.597540	2.889961	H	-3.454688	1.520683	-2.686265
C	-0.224601	-2.098400	2.508213	H	-4.433707	1.765192	0.374962
C	1.590330	-1.936470	-1.838597	H	3.237357	-1.716129	1.750479
C	2.650647	-1.597270	-2.889943	H	4.480707	-1.668668	0.480465
C	-3.793767	1.132239	1.004661	H	-3.181734	1.787191	1.637217
C	-2.421232	1.563056	-3.052321	H	0.306634	1.143922	-2.927892
C	3.826666	-1.048940	1.109264	H	-2.377283	2.310327	-3.854344
C	-1.455626	1.917693	-1.917708	H	4.476146	-0.441720	1.752666
C	-0.038628	2.078989	-2.470642	H	-0.306510	1.143517	2.928203
C	3.793364	1.132685	-1.004881	H	4.455149	0.549525	-1.657728
C	-1.889535	3.241288	-1.272228	H	0.652823	2.314773	-1.651873
C	0.038473	2.078660	2.470891	H	0.013423	2.879093	-3.219092
C	1.455449	1.917693	1.917796	H	-2.903488	3.166526	-0.863508
C	2.421235	1.563175	3.052297	H	2.168937	0.583411	3.476271
C	1.889025	3.241398	1.272322	H	-0.653143	2.314269	1.652210
H	-2.015509	-4.074527	1.873460	H	3.181107	1.787457	-1.637402
H	-2.935321	-3.175178	0.646535	H	-1.209359	3.504515	-0.453308
H	-1.211730	-3.510958	0.389467	H	-1.874938	4.054825	-2.008632
H	2.016341	-4.074323	-1.873397	H	4.433231	1.765821	-0.375294
H	-4.480439	-1.669208	-0.481045	H	3.454647	1.520868	2.686098
H	0.229366	-2.918447	-3.236984	H	-0.013672	2.878755	3.219343
H	-3.236855	-1.716246	-1.750849	H	2.377329	2.310453	3.854316
H	1.212158	-3.510999	-0.389535	H	1.208723	3.504512	0.453469
H	-0.538261	-2.306641	-1.747387	H	2.902956	3.166864	0.863510
H	-2.663183	-2.344447	3.693317	H	1.874317	4.054905	2.008756
H	-4.475855	-0.442063	-1.753033				

Table S 26: Cartesian geometry of **1** in the sextet *S* = 5/2 state (wB97X-D2/def2-SVP).

Element	*x*	*y*	*z*	Element	*x*	*y*	*z*
Co	0.000000	0.000014	0.000000	H	0.161595	2.938214	3.244183
Si	2.704535	-0.000009	-0.000378	H	3.618287	1.663623	2.445367
Si	-2.704534	-0.000009	0.000377	H	-2.849458	3.226887	-0.611086
N	1.471451	0.894009	0.873743	H	0.097806	1.180315	-3.043303
N	-1.471451	0.894040	-0.873711	H	-2.619147	2.476128	-3.672310
N	1.471193	-0.893992	-0.874163	H	-0.609371	2.298065	1.762581
N	-1.471193	-0.894023	0.874132	H	4.491338	-0.480492	1.692463
C	1.867913	3.284491	1.103927	H	2.431943	0.723501	3.388225
C	-1.867907	3.284531	-1.103805	H	-0.097812	1.180207	3.043340
C	3.814530	1.083391	-1.067600	H	-3.618289	1.663717	-2.445300
C	-0.168324	2.110204	-2.518681	H	2.430941	-0.723502	-3.388926
C	1.537748	1.971416	1.839127	H	-2.431951	0.723629	-3.388198
C	2.615075	1.692385	2.899088	H	3.617558	-1.663639	-2.446426
C	0.168322	2.110115	2.518754	H	4.444264	-1.735063	0.438227
C	-1.537748	1.971483	-1.839055	H	-3.213161	1.723902	1.730801
C	-2.615079	1.692495	-2.899023	H	-4.444192	1.734985	0.439486
C	3.814778	-1.083437	1.066559	H	3.213559	-1.723993	1.729852
C	2.614210	-1.692388	-2.899844	H	-0.098716	-1.180199	-3.043292
C	-3.814530	1.083354	1.067636	H	2.618034	-2.475991	-3.673162
C	1.537198	-1.971407	-1.839559	H	-4.490917	0.480369	1.693690
C	0.167569	-2.110105	-2.518778	H	0.098711	-1.180307	3.043254
C	-3.814777	-1.083400	-1.066597	H	-4.491337	-0.480433	-1.692481
C	1.867577	-3.284479	-1.104450	H	-0.609901	-2.298045	-1.762373
C	-0.167571	-2.110194	2.518705	H	0.160622	-2.938209	-3.244199
C	-1.537199	-1.971473	1.839487	H	2.849279	-3.226854	-0.612030
C	-2.614214	-1.692495	2.899780	H	-2.430948	-0.723627	3.388899
C	-1.867573	-3.284519	1.104329	H	0.609901	-2.298105	1.762295
H	1.885014	4.131760	1.807367	H	-3.213557	-1.723932	-1.729913
H	2.849466	3.226863	0.611210	H	1.110923	-3.488954	-0.332555
H	1.111026	3.488976	0.332263	H	1.884459	-4.131753	-1.807889
H	-1.885008	4.131827	-1.807214	H	-4.444262	-1.735048	-0.438288
H	4.444193	1.734999	-0.439427	H	-3.617561	-1.663730	2.446360
H	-0.161596	2.938330	-3.244079	H	-0.160625	-2.938325	3.244095
H	3.213161	1.723963	-1.730741	H	-2.618039	-2.476128	3.673068
H	-1.111017	3.488986	-0.332136	H	-1.110916	-3.488964	0.332429
H	0.609372	2.298123	-1.762503	H	-2.849274	-3.226878	0.611909
H	2.619142	2.475988	3.672405	H	-1.884456	-4.131819	1.807737
H	4.490916	0.480428	-1.693675				

Table S 27: Cartesian geometry of [Co(nor)$_4$] in the doublet S = 1/2 state (B97-D3(BJ)/def2-SVP).

Element	*x*	*y*	*z*	Element	*x*	*y*	*z*
Co	0.026972	0.015848	-0.078538	H	1.382057	3.998888	-1.885640
C	-1.532393	-0.586134	-1.122001	H	-0.227848	2.303252	-1.602866
C	-2.287609	-1.818910	-0.560794	H	0.422429	1.368524	-2.958050
C	-3.089693	-2.080885	-1.856885	H	-1.940428	-0.309813	2.526067
C	-3.770544	-0.712296	-2.090871	H	-2.995757	0.452782	1.295101
C	-2.711908	0.312623	-1.547855	H	-3.153025	1.833920	3.562680
C	1.445324	0.946699	-1.047484	H	-0.879579	1.257336	4.413531
C	2.331350	1.939720	-0.258658	H	-0.696484	2.955809	3.911457
C	2.986080	2.553078	-1.518585	H	0.808238	2.238825	2.164910
C	1.741695	3.036193	-2.292301	H	0.617923	0.580611	2.751357
C	0.699932	1.894395	-2.031181	H	-2.147385	3.975349	2.230155
C	-0.879416	1.156864	1.222814	H	-3.420802	3.049912	1.397311
C	-2.130525	0.615565	1.957217	H	-1.889691	2.445638	-0.272560
C	-2.303178	1.859327	2.858477	H	-0.550030	3.239128	0.575443
C	-0.922808	1.938641	3.544621	H	-2.924397	-1.583789	0.308478
C	0.063163	1.469260	2.419437	H	1.755292	2.682529	0.317002
C	-2.396281	2.985908	1.806831	H	-0.808839	-0.359284	-3.192964
C	-1.383677	2.527952	0.700547	H	0.085318	-1.588398	-2.291397
C	1.094916	-1.398701	0.768210	H	-1.506058	-3.258617	-2.804235
C	-0.916947	-1.152526	-2.436382	H	-2.286692	-2.110784	-3.921322
C	-1.946456	-2.247457	-2.879219	H	2.980533	-0.614119	-1.291090
C	2.503550	0.184849	-1.877137	H	2.065246	-0.282556	-2.774810
C	3.553637	1.299311	-2.223360	H	3.670148	1.453948	-3.311023
C	0.303791	-2.048452	1.939772	H	4.549152	1.040664	-1.819759
C	2.437393	-1.054854	1.460237	H	0.122241	-1.366900	2.786776
C	1.346802	-2.637463	-0.133056	H	-0.656566	-2.495671	1.635515
C	1.389396	-3.102591	2.259191	H	3.268331	-1.012074	0.737517
C	2.620825	-2.205414	2.507747	H	2.398340	-0.075962	1.963168
C	1.557607	-3.803390	0.893827	H	0.478203	-2.857915	-0.772125
H	-1.632109	-2.666100	-0.299803	H	2.212756	-2.496905	-0.799205
H	-3.792451	-2.932131	-1.829078	H	1.161555	-3.791548	3.091292
H	-4.715728	-0.641842	-1.522997	H	2.608839	-1.806474	3.538307
H	-4.015029	-0.549650	-3.155877	H	3.569951	-2.755873	2.378840
H	-2.410712	1.056652	-2.304841	H	2.546035	-4.286635	0.794244
H	-3.132770	0.864612	-0.693416	H	0.791970	-4.588601	0.759242
H	3.054457	1.448897	0.412411	H	3.736135	3.342977	-1.338304
H	1.944827	3.185609	-3.367870				

Table S 28: Cartesian geometry of [Co(nor)$_4$] in the doublet S = 1/2 state (PW6B95-D3(BJ)/def2-SVP).

Element	*x*	*y*	*z*	Element	*x*	*y*	*z*
Co	0.032636	0.034812	-0.080737	H	1.151881	3.898420	-1.860600
C	-1.447372	-0.709987	-1.141299	H	-0.260794	2.023831	-1.677211
C	-2.120074	-1.978440	-0.594935	H	0.614921	1.163129	-2.936204
C	-2.740867	-2.381677	-1.935727	H	-2.115706	-0.311854	2.385303
C	-3.503667	-1.111050	-2.334083	H	-3.151076	0.318344	1.080668
C	-2.656362	0.031398	-1.705673	H	-3.504505	1.780877	3.246608
C	1.532850	0.934061	-0.964166	H	-1.262560	1.371618	4.217994
C	2.269558	2.039333	-0.197132	H	-1.166016	3.049600	3.673577
C	2.890402	2.667220	-1.449186	H	0.434170	2.401697	2.024293
C	1.632663	2.993398	-2.252829	H	0.366203	0.770430	2.670372
C	0.745445	1.743659	-2.018249	H	-2.553461	3.899520	1.879535
C	-1.100841	1.149235	1.070708	H	-3.732271	2.883878	1.044430
C	-2.335521	0.567396	1.767300	H	-2.119795	2.275092	-0.520739
C	-2.628277	1.825643	2.590025	H	-0.871616	3.189807	0.319076
C	-1.306020	2.016877	3.331980	H	-2.857311	-1.771050	0.189360
C	-0.247932	1.585705	2.280488	H	1.604963	2.737116	0.324823
C	-2.731236	2.888655	1.493245	H	-0.648809	-0.492628	-3.161225
C	-1.662104	2.441678	0.458724	H	0.377795	-1.494291	-2.150158
C	1.137567	-1.265703	0.896645	H	-0.942039	-3.418372	-2.559306
C	-0.670375	-1.246167	-2.367847	H	-1.699514	-2.503854	-3.871334
C	-1.483126	-2.492787	-2.796209	H	3.236136	-0.425160	-1.049225
C	2.690190	0.263277	-1.700835	H	2.348007	-0.311806	-2.569554
C	3.595275	1.465777	-2.091885	H	3.695684	1.586312	-3.177627
C	0.310400	-1.900835	2.033674	H	4.607148	1.344736	-1.687008
C	2.393626	-0.820207	1.656883	H	0.043212	-1.201352	2.833634
C	1.510274	-2.513806	0.078732	H	-0.600640	-2.407369	1.696133
C	1.416689	-2.871681	2.463721	H	3.263435	-0.747936	0.995361
C	2.569319	-1.907940	2.748870	H	2.261181	0.166591	2.112737
C	1.715396	-3.610020	1.157842	H	0.701835	-2.809790	-0.597219
H	-1.420187	-2.736901	-0.228588	H	2.400098	-2.349857	-0.537958
H	-3.357308	-3.287880	-1.930415	H	1.171655	-3.533027	3.302559
H	-4.520649	-1.118319	-1.924259	H	2.473616	-1.476114	3.752671
H	-3.597249	-1.019298	-3.423297	H	3.544515	-2.407565	2.702218
H	-2.364862	0.798717	-2.433871	H	2.727214	-4.033121	1.149050
H	-3.229987	0.529937	-0.918413	H	1.015081	-4.440923	1.009147
H	3.013888	1.656442	0.509560	H	3.546676	3.528918	-1.281850
H	1.840600	3.165466	-3.315817				

Table S 29: Cartesian geometry of [Co(nor)$_4$] in the doublet S = 1/2 state (wB97X-D2/def2-SVP).

Element	*x*	*y*	*z*	Element	*x*	*y*	*z*
Co	0.029732	0.026794	-0.075490	H	1.152375	3.959218	-1.938470
C	-1.435251	-0.728922	-1.133326	H	-0.302593	2.133139	-1.682523
C	-2.102433	-1.999608	-0.564826	H	0.502889	1.230078	-2.965076
C	-2.780781	-2.394186	-1.886229	H	-2.068141	-0.341030	2.431769
C	-3.561251	-1.115211	-2.240495	H	-3.134071	0.270600	1.137163
C	-2.662101	0.024245	-1.669843	H	-3.475238	1.742382	3.309745
C	1.464554	0.966275	-1.001989	H	-1.209912	1.380049	4.260280
C	2.219940	2.062508	-0.226091	H	-1.137509	3.056308	3.688755
C	2.860470	2.685802	-1.477032	H	0.467811	2.392795	2.034846
C	1.612825	3.027911	-2.303100	H	0.400417	0.761754	2.695369
C	0.682054	1.806612	-2.047846	H	-2.589548	3.878828	1.913700
C	-1.084353	1.131402	1.092223	H	-3.752175	2.824591	1.089925
C	-2.308878	0.536417	1.812375	H	-2.124941	2.269728	-0.492772
C	-2.607277	1.798063	2.637195	H	-0.885579	3.193405	0.359868
C	-1.271048	2.013780	3.362254	H	-2.810822	-1.790042	0.250164
C	-0.217505	1.577094	2.300952	H	1.559462	2.768290	0.298554
C	-2.744690	2.858025	1.532907	H	-0.640057	-0.524825	-3.172217
C	-1.667770	2.430390	0.491527	H	0.344634	-1.588469	-2.175300
C	1.176252	-1.260099	0.860816	H	-1.029947	-3.450752	-2.636707
C	-0.693120	-1.285749	-2.381817	H	-1.833822	-2.470483	-3.877904
C	-1.561613	-2.502183	-2.812075	H	3.141530	-0.439388	-1.118816
C	2.617364	0.283486	-1.755762	H	2.262621	-0.262581	-2.642507
C	3.561422	1.472045	-2.113365	H	3.696948	1.598703	-3.198372
C	0.365372	-1.908030	2.013976	H	4.561214	1.326385	-1.676880
C	2.440245	-0.793585	1.613211	H	0.085004	-1.207093	2.813336
C	1.562900	-2.517329	0.047089	H	-0.540849	-2.435636	1.683518
C	1.493842	-2.860055	2.444530	H	3.308018	-0.719319	0.942751
C	2.637748	-1.872213	2.717591	H	2.302151	0.199754	2.061843
C	1.792572	-3.606849	1.135640	H	0.752999	-2.830253	-0.625207
H	-1.391455	-2.765216	-0.222516	H	2.449740	-2.347893	-0.578748
H	-3.400768	-3.301898	-1.859188	H	1.264280	-3.521475	3.292355
H	-4.551403	-1.114561	-1.760130	H	2.537804	-1.432023	3.721716
H	-3.727829	-1.024432	-3.324805	H	3.624465	-2.357576	2.671781
H	-2.386284	0.773112	-2.427767	H	2.813017	-4.018894	1.119441
H	-3.194705	0.553220	-0.869337	H	1.098848	-4.450632	0.999998
H	2.955615	1.666357	0.487795	H	3.526827	3.542596	-1.301416
H	1.839263	3.169546	-3.370714				

Table S 30: Cartesian geometry of [Co(nor)$_4$] in the sextet S = 5/2 state (B97-D3(BJ)/def2-SVP).

Element	*x*	*y*	*z*	Element	*x*	*y*	*z*
Co	0.035893	-0.007869	-0.047784	H	4.683346	0.718605	-1.370717
C	-1.080046	1.351601	-1.175697	H	2.512698	1.566937	-1.031483
C	-2.556551	1.513616	-0.747061	H	2.006402	1.058885	-2.655047
C	-2.895716	2.517751	-1.878607	H	-1.400424	1.007941	2.776187
C	-1.838264	3.621605	-1.643619	H	-0.917867	2.500807	1.906186
C	-0.586973	2.814235	-1.139805	H	-0.153822	2.676940	4.460659
C	1.755588	-0.504267	-1.121083	H	0.105391	0.228766	4.869129
C	2.872936	-1.171638	-0.288197	H	1.791540	0.791668	4.766394
C	3.843962	-1.306591	-1.489625	H	2.048367	-0.498020	2.724177
C	3.942150	0.159609	-1.970341	H	0.363832	-1.024123	2.904517
C	2.490667	0.711917	-1.726845	H	2.418137	2.819202	3.588425
C	0.588641	0.859076	1.777516	H	1.229075	3.826303	2.726086
C	-0.543018	1.639438	2.485668	H	1.561125	2.568462	0.766482
C	0.340709	2.064382	3.686421	H	2.696208	1.481528	1.588902
C	0.848639	0.697059	4.198653	H	-2.675776	1.937926	0.265329
C	1.017170	-0.137432	2.877529	H	3.270054	-0.527303	0.515055
C	1.504675	2.783808	2.968255	H	-0.314357	1.204957	-3.235984
C	1.693216	1.935079	1.659365	H	-1.349839	-0.156922	-2.772818
C	-1.099503	-1.730051	0.311927	H	-3.298890	1.006532	-3.416743
C	-1.213249	0.931334	-2.658579	H	-2.299631	2.359146	-4.003716
C	-2.493830	1.708426	-3.132379	H	0.857337	-2.351639	-1.924353
C	1.532912	-1.541410	-2.243800	H	1.088230	-1.081313	-3.142004
C	2.980100	-2.100526	-2.496869	H	3.317241	-1.952941	-3.538540
C	-2.145578	-1.590926	1.442476	H	3.028154	-3.183994	-2.284455
C	-0.256931	-2.933417	0.797326	H	-1.708658	-1.363619	2.429831
C	-1.980167	-2.211977	-0.862734	H	-2.932395	-0.847990	1.223385
C	-2.635372	-3.057298	1.331496	H	0.228909	-3.454538	-0.044926
C	-1.314280	-3.833946	1.531809	H	0.543928	-2.626783	1.490554
C	-3.035622	-3.139999	-0.159356	H	-2.488354	-1.372657	-1.364209
H	-3.146505	0.583269	-0.812602	H	-1.390496	-2.740173	-1.630982
H	-3.934774	2.890763	-1.901632	H	-3.438608	-3.355192	2.028130
H	-2.182048	4.338142	-0.875813	H	-1.075579	-3.925293	2.607073
H	-1.630802	4.197958	-2.562942	H	-1.365138	-4.856388	1.116623
H	0.304775	2.962697	-1.771576	H	-3.009893	-4.177368	-0.538277
H	-0.320411	3.129109	-0.117635	H	-4.061289	-2.758268	-0.312413
H	2.579921	-2.143509	0.145194	H	4.821573	-1.772668	-1.274096
H	4.252626	0.229862	-3.028231				

Table S 31: Cartesian geometry of [Co(nor)$_4$] in the sextet S = 5/2 state (PW6B95-D3(BJ)/def2-SVP).

Element	*x*	*y*	*z*	Element	*x*	*y*	*z*
Co	0.025056	-0.002354	-0.041567	H	4.616491	0.121691	-1.640434
C	-0.886755	1.689970	-0.840859	H	2.690979	1.248184	-0.946584
C	-2.268361	2.035666	-0.280647	H	2.003815	1.162429	-2.568937
C	-2.466870	3.238416	-1.215919	H	-1.016475	0.729721	3.009728
C	-1.218337	4.076856	-0.918637	H	-0.327345	2.242955	2.362298
C	-0.132722	3.001485	-0.622667	H	0.607641	1.828053	4.807168
C	1.556613	-0.582538	-1.329931	H	0.442484	-0.642184	4.780855
C	2.558291	-1.568989	-0.727704	H	2.178263	-0.360627	4.617085
C	3.405992	-1.652012	-2.006429	H	2.070301	-1.284357	2.394630
C	3.749036	-0.176852	-2.241686	H	0.344442	-1.541727	2.640157
C	2.463730	0.565256	-1.772956	H	3.079925	1.679350	3.743103
C	0.833946	0.432981	1.832382	H	2.058722	3.006289	3.180677
C	-0.083679	1.246050	2.750190	H	2.018549	2.097759	1.034854
C	0.927787	1.292884	3.905931	H	2.984555	0.714815	1.542812
C	1.207550	-0.195451	4.134130	H	-2.252452	2.307586	0.782567
C	1.139407	-0.791312	2.698466	H	3.114220	-1.163645	0.126784
C	2.146666	1.913477	3.216877	H	-0.262656	1.751644	-2.932757
C	2.092575	1.303988	1.786684	H	-1.489115	0.532188	-2.609747
C	-1.369996	-1.541560	0.135750	H	-3.221474	2.083154	-2.897279
C	-1.162084	1.543669	-2.341285	H	-2.039876	3.310281	-3.366724
C	-2.294010	2.581041	-2.588027	H	0.274960	-2.038574	-2.342259
C	1.077231	-1.329328	-2.575564	H	0.690831	-0.643002	-3.337304
C	2.358591	-2.081469	-3.038715	H	2.657406	-1.819811	-4.061029
C	-2.294688	-1.426124	1.351536	H	2.208625	-3.167714	-3.010993
C	-0.732849	-2.920326	0.339345	H	-1.771558	-1.447910	2.314997
C	-2.381863	-1.655991	-1.007142	H	-2.944829	-0.542093	1.323127
C	-3.036180	-2.737812	1.054041	H	-0.405590	-3.356949	-0.612032
C	-1.875506	-3.735995	1.009092	H	0.148924	-2.880221	0.989056
C	-3.527678	-2.498728	-0.376671	H	-2.760169	-0.675850	-1.315182
H	-3.023370	1.255346	-0.434047	H	-1.947340	-2.127636	-1.896293
H	-3.408273	3.788206	-1.103413	H	-3.830701	-3.013975	1.756630
H	-1.377955	4.724757	-0.048224	H	-1.592855	-4.046615	2.022577
H	-0.952851	4.725985	-1.761881	H	-2.133615	-4.643652	0.450272
H	0.745460	3.084820	-1.273482	H	-3.705796	-3.439662	-0.911277
H	0.216684	3.096802	0.411093	H	-4.471933	-1.940725	-0.377959
H	2.113533	-2.530402	-0.442563	H	4.282067	-2.309226	-1.964099
H	3.995808	0.024609	-3.291164				

Table S 32: Cartesian geometry of [Co(nor)$_4$] in the sextet S = 5/2 state (wB97X-D2/def2-SVP).

Element	*x*	*y*	*z*	Element	*x*	*y*	*z*
Co	0.029999	-0.006349	-0.044939	H	4.668932	0.341669	-1.451267
C	-0.950766	1.549678	-1.001301	H	2.626982	1.378025	-0.945737
C	-2.381741	1.830484	-0.513607	H	2.026158	1.100642	-2.586437
C	-2.631125	2.951348	-1.542067	H	-1.206384	0.867029	2.895561
C	-1.457084	3.905388	-1.255741	H	-0.601793	2.376158	2.150945
C	-0.301106	2.934834	-0.855401	H	0.256690	2.225141	4.665573
C	1.646270	-0.571003	-1.213513	H	0.270687	-0.252204	4.835342
C	2.695297	-1.431307	-0.492412	H	1.994081	0.142683	4.714592
C	3.606711	-1.548672	-1.730274	H	2.048259	-0.962891	2.565037
C	3.855073	-0.067833	-2.069117	H	0.329540	-1.325264	2.762907
C	2.491929	0.608864	-1.719692	H	2.787795	2.192095	3.710619
C	0.717474	0.618151	1.812956	H	1.689360	3.386689	2.997563
C	-0.299635	1.432498	2.630885	H	1.826830	2.303042	0.926784
C	0.656616	1.646074	3.820747	H	2.863854	1.037478	1.594562
C	1.032969	0.198251	4.181310	H	-2.427791	2.176182	0.530982
C	1.070026	-0.514029	2.792747	H	3.180159	-0.918459	0.352507
C	1.860812	2.307167	3.128761	H	-0.253020	1.508698	-3.081223
C	1.918850	1.579159	1.749493	H	-1.407654	0.226274	-2.707653
C	-1.262316	-1.613494	0.194169	H	-3.235396	1.629764	-3.172011
C	-1.160147	1.278386	-2.503124	H	-2.119896	2.908763	-3.685052
C	-2.354978	2.214779	-2.863915	H	0.524710	-2.210705	-2.149424
C	1.278510	-1.455805	-2.415394	H	0.865153	-0.865065	-3.245783
C	2.637622	-2.137177	-2.771482	H	2.961663	-1.927014	-3.802071
C	-2.250947	-1.469007	1.364190	H	2.570736	-3.230937	-2.666764
C	-0.543562	-2.932294	0.537319	H	-1.768265	-1.376957	2.348966
C	-2.218402	-1.887702	-0.979666	H	-2.957435	-0.633363	1.239466
C	-2.892401	-2.852534	1.143539	H	-0.143489	-3.421248	-0.363535
C	-1.662716	-3.772973	1.227352	H	0.306448	-2.778148	1.218104
C	-3.337577	-2.758047	-0.326385	H	-2.645339	-0.958506	-1.381957
H	-3.071653	0.981300	-0.633126	H	-1.713533	-2.396983	-1.813811
H	-3.617588	3.435347	-1.501291	H	-3.700625	-3.127722	1.836390
H	-1.701484	4.590842	-0.429953	H	-1.406939	-3.983009	2.277420
H	-1.208090	4.525094	-2.130531	H	-1.834452	-4.741129	0.733060
H	0.590643	3.040162	-1.490755	H	-3.431946	-3.750239	-0.793219
H	0.013295	3.127616	0.180253	H	-4.318546	-2.265331	-0.407167
H	2.307305	-2.401067	-0.144667	H	4.527900	-2.134746	-1.599883
H	4.142238	0.072611	-3.122236				

Table S 33: Cartesian geometry of R = CH_3 analogue of **1** in the doublet *S* = 1/2 state.*

Element	*x*	*y*	*z*
Co	0.009157	0.000226	-0.00004
Si	2.661522	0.000011	-0.000398
Si	-2.643179	0.000023	0.000372
N	1.393302	0.839355	0.828745
N	-1.372347	0.837524	-0.829781
N	1.392915	-0.839096	-0.829212
N	-1.371923	-0.837231	0.830156
C	3.757946	1.071634	-1.07908
C	1.21788	1.860537	1.808936
C	-1.256415	1.861861	-1.811906
C	3.757987	-1.071803	1.078067
C	-3.723964	1.078091	1.086028
C	1.217038	-1.860543	-1.809049
C	-3.724014	-1.078249	-1.085039
C	-1.255483	-1.861721	1.812064
H	4.408375	1.709422	-0.463913
H	3.152946	1.716588	-1.72715
H	4.408837	0.453854	-1.713803
H	4.408988	-0.454108	1.712761
H	4.408308	-1.709614	0.46281
H	-3.104136	1.714863	1.728489
H	-4.372559	1.724335	0.478378
H	3.153005	-1.716739	1.72617
H	-4.373497	0.465894	1.727061
H	-4.37367	-0.466144	-1.726035
H	-3.1042	-1.714993	-1.727541
H	-4.372484	-1.724519	-0.477285
H	-0.203279	2.0866	-2.034543
H	-1.722876	2.8067	-1.483079
H	-1.732252	1.578175	-2.766535
H	2.183912	-2.232298	-2.185353
H	0.649063	-1.505795	-2.68442
H	0.672191	-2.732188	-1.410636
H	2.184923	2.232	2.185094
H	0.650078	1.505626	2.684355
H	0.673081	2.732399	1.410938
H	-0.202233	-2.086192	2.034428
H	-1.721747	-2.806637	1.483173
H	-1.731191	-1.57833	2.766845

* This geometry had 3 small negative frequencies and was not a minimum on the potential energy surface. It is included for completeness.

Table S 34: Cartesian geometry of R = CH_3 analogue of **1** in the quartet *S* = 3/2 state.

Element	*x*	*y*	*z*
Co	-0.0001200865	0.0000096662	-0.0002176169
Si	2.6306697348	0.0000008114	-0.0003670218
Si	-2.6300308482	0.0001109279	0.0003923306
N	1.3796951778	1.1248531236	0.4622029570
N	-1.3792849016	1.1240363565	-0.4653228344
N	1.3796258736	-1.1247950301	-0.4629216122
N	-1.3791622766	-1.1240018380	0.4653432183
C	3.7080473809	0.6340526963	-1.3957140630
C	1.4061583298	2.4626551471	0.9515044351
C	-1.4063039632	2.4611944915	-0.9558958976
C	3.7080123595	-0.6341899471	1.3949485716
C	-3.7075009083	0.6404398834	1.3929625292
C	1.4060808967	-2.4626789056	-0.9520067282
C	-3.7085573135	-0.6401607033	-1.3913745678
C	-1.4061478437	-2.4612439599	0.9556903616
H	4.4092890137	1.3964837971	-1.0297488373
H	3.0865002436	1.0860786275	-2.1779880441
H	4.3064892981	-0.1672888881	-1.8503499338
H	4.3065774742	0.1670537634	1.8495918945
H	4.4091401277	-1.3967043240	1.0289350841
H	-3.0858971813	1.0940578928	2.1743098600
H	-4.4053242474	1.4038411526	1.0223460223
H	3.0864322201	-1.0861646667	2.1772263432
H	-4.3096945982	-0.1566984363	1.8499919868
H	-4.3110779633	0.1569693286	-1.8479819901
H	-3.0875678712	-1.0938578883	-2.1731677091
H	-4.4061137338	-1.4035138588	-1.0201512323
H	-0.4052332790	2.7969328452	-1.2578534779
H	-1.7798982624	3.1786449867	-0.2031889714
H	-2.0646912640	2.5672449224	-1.8346204305
H	-0.4050802918	-2.7970011251	1.2576388847
H	-1.7796499618	-3.1785810496	0.2028271501
H	-2.0645982962	-2.5674861312	1.8343433468
H	1.7757509856	-3.1795006602	-0.1967339904
H	2.0680601365	-2.5711570675	-1.8276461112
H	0.4056693056	-2.7974534823	-1.2570795852
H	1.7758954189	3.1795765348	0.1963605963
H	2.0680777156	2.5709836657	1.8272075940
H	0.4057333994	2.7974073413	1.2565574891

Table S 35: Cartesian geometry of R = CH_2CH_3 analogue of **1** in the doublet *S* = 1/2 state.

Element	*x*	*y*	*z*	Element	*x*	*y*	*z*
Co	0.0000681559	-0.0468459652	0.0001931889	H	-0.7061910846	2.5443190720	1.2727432875
Si	2.6562101247	-0.0384564460	0.0176918823	H	4.3807153685	-0.5140563032	1.7508138105
Si	-2.6560650195	-0.0383179680	-0.0173256435	H	-0.6915583413	1.3422045711	2.5731687316
N	1.3764209359	0.7915985835	0.8535572684	H	4.3964338025	-1.7487546830	0.4777770753
N	-1.3762287667	0.7917959031	-0.8530320790	H	-3.1371772370	1.6895487183	1.6944251621
N	1.3958114954	-0.8703404405	-0.8353392430	H	-4.3983595929	1.6798486057	0.4357166400
N	-1.3957214926	-0.8704803186	0.8355258994	H	3.1251415419	-1.7786472409	1.7227648074
C	3.7475516469	1.0445028526	-1.0519125590	H	-4.3956932068	0.4297479446	1.6923352868
C	0.1635632718	2.2138047855	-2.1662072189	H	-4.3805557177	-0.5135339294	-1.7506001328
C	1.2709708484	1.8495499144	1.8137202051	H	2.5630530329	-3.3959476130	-0.9536806267
C	-0.1629968862	2.2136741749	2.1671498264	H	-3.1250897844	-1.7782262149	-1.7226251179
C	-1.2704931795	1.8505269872	-1.8122962574	H	0.8750178079	-3.5252363843	-0.4581838195
C	3.7396301146	-1.1210387955	1.0965106543	H	1.3495170164	-4.0453491565	-2.0870919454
C	-3.7472476782	1.0446365362	1.0524178475	H	-4.3964661132	-1.7484025578	-0.4777309037
C	1.2504941094	-1.8912016055	-1.8251954859	H	-0.8749797561	-3.5252791014	0.4573083074
C	-3.7395677438	-1.1206510988	-1.0963247362	H	-2.5630537829	-3.3961242941	0.9526938127
C	1.5279249411	-3.2954618953	-1.3055181181	H	-1.3496512773	-4.0460120894	2.0859659180
C	-1.2505253224	-1.8917700551	1.8249570943	H	0.2280767378	-1.8589451129	-2.2345198950
C	-1.5279579772	-3.2958102612	1.3046710615	H	1.9139174530	-1.6835556879	-2.6836508632
H	4.3987998565	1.6795619081	-0.4352007811	H	1.7824728887	2.7540360236	1.4358437561
H	0.1989085499	3.0191147635	-2.9092487474	H	1.8034818654	1.5680773463	2.7400836504
H	3.1375949556	1.6895759055	-1.6938654277	H	-1.8038067269	1.5703115896	-2.7385723546
H	0.7073814621	2.5433505023	-1.2717864761	H	-1.7810097515	2.7551215746	-1.4333234321
H	4.3958601289	0.4295588735	-1.6919200519	H	-1.9140118890	-1.6844721463	2.6834453195
H	-0.1981082322	3.0184442306	2.9107845133	H	-0.2281386725	-1.8597277263	2.2343752055
H	0.6913371204	1.3422537246	-2.5730782959				

Table S 36: Cartesian geometry of R = CH_2CH_3 analogue of **1** in the quartet *S* = 3/2 state.

Element	x	y	z	Element	x	y	z
Co	0.000125150	-0.081906606	0.000062529	H	-0.555171352	2.276462546	1.857358596
Si	2.649017111	-0.071592529	0.051778330	H	4.309004696	0.441975589	1.821879088
Si	-2.648743124	-0.071230813	-0.051440581	H	0.768448490	1.918523680	2.977696481
N	1.384595108	1.054632586	0.440665965	H	4.539256704	-1.209294702	1.234031558
N	-1.384212746	1.054909557	-0.440189959	H	-3.051255238	0.630212362	2.289685989
N	1.405782096	-1.237948625	-0.318019367	H	-4.331576455	1.257503852	1.227239038
N	-1.405644077	-1.237888830	0.317851945	H	3.198527078	-0.842236357	2.346736637
C	3.696116628	0.386660414	-1.435248374	H	-4.359002658	-0.437993506	1.729453759
C	-0.463349731	2.564464188	-2.143554789	H	-4.309057050	0.442907642	-1.821098130
C	1.405804144	2.387317023	0.963373717	H	3.273660339	-3.221473417	0.057864729
C	0.463496222	2.563303600	2.144600983	H	-3.198672765	-0.841144952	-2.346545908
C	-1.405316852	2.387899684	-0.962151922	H	1.779960628	-3.678577668	0.888842534
C	3.777439692	-0.463163537	1.496308176	H	2.264395830	-4.524282988	-0.600348910
C	-3.695541219	0.386800941	1.435862902	H	-4.539195146	-1.208539005	-1.233703493
C	1.460697212	-2.530707401	-0.923094723	H	-1.779905783	-3.678093028	-0.889726812
C	-3.777426543	-0.462326848	-1.495896095	H	-3.273616766	-3.221375798	-0.058559954
C	2.237761759	-3.548639667	-0.099487709	H	-2.264244846	-4.524331963	0.599189761
C	-1.460672044	-2.530804529	0.922586836	H	0.440311139	-2.897825165	-1.101922767
C	-2.237691582	-3.548518045	0.098657528	H	1.926430853	-2.458035638	-1.923333908
H	4.332070384	1.257365264	-1.226381951	H	1.140355770	3.115847277	0.177943250
H	-0.452718205	3.604248192	-2.493526158	H	2.429246175	2.658222524	1.274507308
H	3.052035111	0.630160034	-2.289200069	H	-2.428812095	2.659144229	-1.272821486
H	0.555366981	2.277266983	-1.856810534	H	-1.139539945	3.115963870	-0.176406153
H	4.359674911	-0.438071704	-1.728796914	H	-1.926498570	-2.458383324	1.922800901
H	0.452937094	3.602862997	2.495250926	H	-0.440318413	-2.898008982	1.101422906

Table S 37: Cartesian geometry of R = $CH(CH_3)_2$ analogue of **1** in the doublet *S* = 1/2 state.

Element	x	y	z	Element	x	y	z
Co	-0.039278067	-0.049707536	-0.017289285	H	-0.147246029	-3.126708746	-2.858711516
Si	-2.719529110	-0.091423509	-0.015234995	H	-0.653363685	-1.426675038	-2.579741845
Si	2.641066142	-0.010293983	-0.004773227	H	-3.158159519	-1.785158373	-1.767547204
N	-1.422411186	-0.920316076	0.806528935	H	-1.492493384	-0.783741024	3.521522576
N	1.373479261	-0.830181584	-0.879484101	H	-0.645511196	-2.565280245	-1.227017734
N	-1.449880518	0.773840957	-0.841879534	H	2.083300254	-2.334236957	-3.831201302
N	1.341893215	0.774909867	0.855848216	H	1.601551109	-0.629049448	-3.572278380
C	0.117346880	-2.415940101	1.965130335	H	-4.546520962	-0.662264542	-1.620394227
C	-1.327877339	-1.999317716	1.739938924	H	-3.058872499	-1.346486288	2.886780135
C	-2.010420843	-1.636719989	3.055623364	H	-4.557153166	0.422848634	1.597121884
C	-0.116802232	-2.285044717	-2.149089413	H	3.120490661	-1.241697277	-2.871633319
C	-3.783598401	-1.224542843	-1.057347616	H	3.127321692	-1.792391281	1.646644011
C	2.077010928	-1.505310292	-3.104134389	H	-4.373050278	1.705684886	0.375386486
C	1.318420934	-1.890085001	-1.837517434	H	-3.210502740	1.597676228	1.729549557
C	-3.817727812	1.008731183	1.026733080	H	4.297788746	-1.822018491	0.295358309
C	3.736477357	-1.163722960	0.980883156	H	-0.762413978	1.350177498	2.469212785
C	-1.392017819	1.876968045	-1.749571269	H	1.419816066	0.515857938	3.559744144
C	-0.200071849	2.212866382	2.082949569	H	4.470744636	-0.608047626	1.587043451
C	1.932631165	1.392089221	3.132881382	H	-0.681599855	2.516638679	1.142825515
C	1.245859138	1.811236255	1.836356417	H	-3.189711292	1.277533543	-2.820219349
C	-2.145229187	1.551293897	-3.035783173	H	0.570057478	2.519480072	-1.099573116
C	3.707776553	1.175626063	-0.983453642	H	2.982250739	1.115287397	2.949179909
C	0.044216991	2.286753345	-2.036417318	H	-0.259818934	3.039517616	2.808165813
H	1.778756501	2.709135783	1.449484386	H	4.470968731	0.643329382	-1.574554396
H	1.821688070	-2.784397284	-1.404754593	H	1.913733419	2.207392873	3.875035751
H	-1.897713643	2.749781879	-1.277532755	H	-1.667853645	0.697440429	-3.541815916
H	-1.864711110	-2.877145565	1.314436250	H	0.581980797	1.451294631	-2.508602029
H	0.176210024	-3.272906060	2.654333556	H	3.083750578	1.773654984	-1.663747706
H	0.595540955	-2.681100789	1.011739968	H	-2.148316845	2.412867759	-3.723866566
H	0.683253887	-1.572005960	2.386143070	H	4.233408998	1.861385668	-0.296588951
H	-1.994315720	-2.485385563	3.759448089	H	0.077008761	3.163438359	-2.702174516
H	-4.309657824	-1.946146585	-0.408578210				

Table S 38: Cartesian geometry of R = $CH(CH_3)_2$ analogue of **1** in the quartet *S* = 3/2 state.

Element	x	y	z	Element	x	y	z
Co	0.002147185	0.000474666	-0.000820643	H	3.488285802	2.701543573	1.088140446
Si	2.627986473	-0.000985770	0.000889728	H	-2.837780303	3.231109315	0.201799820
Si	-2.645855835	0.000094622	-0.000191762	H	0.044825709	1.791865799	-2.672024054
N	1.378398797	1.051255112	0.614395001	H	-0.674135552	2.105111057	2.039437369
N	-1.394878022	1.109535610	-0.513459444	H	4.347348326	-0.100502835	1.823256339
N	1.377893862	-1.050436494	-0.616326727	H	3.057529210	1.893585429	2.613559108
N	-1.394361503	-1.108507351	0.513273508	H	0.554390049	1.485334590	3.163235827
C	-1.957768452	3.498795418	-0.395843394	H	3.051508404	-1.887956368	-2.621766279
C	3.711685530	0.812846274	-1.292928294	H	3.490452103	-2.694003356	-1.097779360
C	-0.246741635	2.644411852	-2.047084340	H	4.360372227	-1.568368834	0.833299571
C	1.360079785	2.300742580	1.329108915	H	-3.128367669	1.082928215	2.181117888
C	2.737464132	2.648931336	1.885246147	H	-4.491813316	1.307152681	1.056447144
C	0.325036200	2.287356258	2.449562034	H	3.070972223	-1.325799868	2.035871649
C	-1.517851606	2.326636394	-1.272140655	H	0.546337820	-1.485438487	-3.161990213
C	3.705076375	-0.818938500	1.297646393	H	2.721179273	-3.615440687	-2.401108983
C	2.736490915	-2.644161202	-1.892162439	H	-4.269577006	-0.244737415	1.871323494
C	-3.732710066	0.594496163	1.406934331	H	0.050119373	-1.788325137	2.668640797
C	1.360042456	-2.299885789	-1.331144478	H	-4.269518696	0.243263500	-1.872147896
C	0.321456713	-2.288729263	-2.448305603	H	-0.677062155	-2.109812304	-2.035181253
C	-3.731821930	-0.595340256	-1.407586339	H	0.309224630	-3.242996337	-2.989842055
C	-0.243662749	-2.642124325	2.046403852	H	0.582128237	-2.852469870	1.358158706
C	-1.515947197	-2.325075617	1.273046839	H	-3.126921155	-1.083233747	-2.181672017
C	-1.956567100	-3.498289869	0.398481291	H	-4.490236193	-1.308772136	-1.057159779
H	-2.210809111	4.370453254	-1.011995749	H	-0.386636021	-3.514159174	2.695616996
H	4.367783345	1.560629917	-0.827014381	H	-1.156619890	-3.787011968	-0.293036759
H	-0.390102123	3.518194270	-2.693864837	H	-2.837228994	-3.231547329	-0.198610933
H	3.081740948	1.320842273	-2.033861640	H	-2.208716287	-4.369315674	1.015879176
H	-1.157173247	3.786878536	0.295196573	H	1.073688589	3.112256482	0.634162447
H	0.580689011	2.851991084	-1.359939959	H	-2.317923968	-2.169925229	2.019740656
H	2.720899488	3.620004464	2.394568510	H	1.077955543	-3.112361330	-0.635472146
H	4.353416088	0.091811626	-1.815609631	H	-2.320851068	2.171924887	-2.017839382
H	0.311434024	3.242035285	2.990360869				

13. References

[1] a) V. Passarelli, F. Benetollo, P. Zanella, G. Carta, G. Rossetto, *Dalton Trans.* **2003**, 1411; b) A. D. Horton, J. de With, *Organometallics* **1997**, *16*, 5424.

[2] L. Sacconi, I. Bertini, F. Mani, *Inorg. Chem.* **1967**, *6*, 262.

[3] K. Väyrynen, T. Hatanpää, M. Mattinen, M. Heikkilä, K. Mizohata, K. Meinander, J. Räisänen, M. Ritala, M. Leskelä, *Chem. Mater.* **2018**, *30*, 3499.

[4] T. J. Knisley, M. J. Saly, M. J. Heeg, J. L. Roberts, C. H. Winter, *Organometallics* **2011**, *30*, 5010.

[5] J. Zemann, *Acta Cryst* **1965**, *18*, 139.

[6] G. V. Kunte, S. A. Shivashankar, A. M. Umarji, *Meas. Sci. Technol.* **2008**, *19*, 25704.

[7] C. Colominas, K. H. Lau, D. L. Hildenbrand, S. Crouch-Baker, A. Sanjurjo, *J. Chem. Eng. Data* **2001**, *46*, 446.

[8] Bruker AXS Inc., *APEX 3*, Madison, Wisconsin, USA, **2018**.

[9] G. M. Sheldrick, *Acta crystallographica. Section A, Foundations and advances* **2015**, *71*, 3.

[10] G. M. Sheldrick, *Acta crystallographica. Section C, Structural chemistry* **2015**, *71*, 3.

[11] C. B. Hübschle, G. M. Sheldrick, B. Dittrich, *Journal of applied crystallography* **2011**, *44*, 1281.

[12] O. V. Dolomanov, L. J. Bourhis, R. J. Gildea, J. A. K. Howard, H. Puschmann, *J Appl Crystallogr* **2009**, *42*, 339.

[13] Crystal Impact GbR (Klaus Brandenburg), *Diamond 3.2k*, Bonn, Germany, **2014**.

[14] G. Sauerbrey, *Z. Physik (Zeitschrift für Physik)* **1959**, *155*, 206.

[15] a) M. B. E. Griffiths, Z. S. Dubrawski, G. Bačić, A. Japahuge, J. D. Masuda, T. Zeng, S. T. Barry, *Eur. J. Inorg. Chem.* **2019**, *2019*, 4927; b) M. A. Land, K. N. Robertson, S. T. Barry, *Organometallics* **2019**.

[16] 1. Gaussian 16, Revision C.01, Frisch, M. J., G. W. Trucks, H. B. Schlegel, G. E. Scuseria, M. A. Robb, J. R. Cheeseman, G. Scalmani, V. Barone, G. A. Petersson, H. Nakatsuji et al.

[17] E. K. Byrne, D. S. Richeson, K. H. Theopold, *J. Chem. Soc., Chem. Commun.* **1986**, 1491.

[18] Perdew, Burke, Ernzerhof, *Physical review letters* **1996**, *77*, 3865.

[19] F. Weigend, R. Ahlrichs, *Physical chemistry chemical physics : PCCP* **2005**, *7*, 3297.

[20] A. Austin, G. A. Petersson, M. J. Frisch, F. J. Dobek, G. Scalmani, K. Throssell, *Journal of chemical theory and computation* **2012**, *8*, 4989.

[21] A. D. Becke, *The Journal of Chemical Physics* **1997**, *107*, 8554.

[22] Y. Zhao, D. G. Truhlar, *J. Phys. Chem. A* **2005**, *109*, 5656.

[23] J.-D. Chai, M. Head-Gordon, *Physical chemistry chemical physics : PCCP* **2008**, *10*, 6615.

[24] F. Neese, *WIREs Comput Mol Sci* **2018**, *8*, e1327.

[25] Becke, *Physical review. A, General physics* **1988**, *38*, 3098.

[26] Lee, Yang, Parr, *Physical review. B, Condensed matter* **1988**, *37*, 785.

[27] M. Swart, A. R. Groenhof, A. W. Ehlers, K. Lammertsma, *J. Phys. Chem. A* **2004**, *108*, 5479.

[28] F. Neese, F. Wennmohs, A. Hansen, U. Becker, *Chemical Physics* **2009**, *356*, 98.

[29] M. Swart, M. Gruden, *Accounts of chemical research* **2016**, *49*, 2690.

[30] C. Riplinger, B. Sandhoefer, A. Hansen, F. Neese, *The Journal of Chemical Physics* **2013**, *139*, 134101.

[31] F. Neese, *Journal of the American Chemical Society* **2006**, *128*, 10213.

[32] T. Lu, F. Chen, *Journal of computational chemistry* **2012**, *33*, 580.

[33] I. Mayer, *Journal of computational chemistry* **2007**, *28*, 204.

[34] a) R. F. W. Bader, *The journal of physical chemistry. A* **2007**, *111*, 7966; b) R. F. W. Bader, P. M. Beddall, *The Journal of Chemical Physics* **1972**, *56*, 3320.
[35] E. R. Johnson, S. Keinan, P. Mori-Sánchez, J. Contreras-García, A. J. Cohen, W. Yang, *Journal of the American Chemical Society* **2010**, *132*, 6498.
[36] L. Zhang, Y. Liu, L. Deng, *Journal of the American Chemical Society* **2014**, *136*, 15525.
[37] T. R. Cundari, *Chemical reviews* **2000**, *100*, 807.
[38] a) D. J. Liptrot, J.-D. Guo, S. Nagase, P. P. Power, *Angew. Chem.* **2016**, *128*, 14986; b) H. Li, Y. Hu, Di Wan, Z. Zhang, Q. Fan, R. B. King, H. F. Schaefer, *The journal of physical chemistry. A* **2019**, *123*, 9514.
